# Domination of Early Adopters: A Bibliometric Review of Team-Based Learning Research in Health Professions Education 2005–2024

**DOI:** 10.5334/pme.1874

**Published:** 2025-12-04

**Authors:** Anita Pienkowska, Simon Collingwood Kitto, Jennifer Anne Cleland

**Affiliations:** 1 Lee Kong Chian School of Medicine, Nanyang Technological University, Singapore

## Abstract

**Background:**

As team-based learning (TBL) has gained traction in health profession education (HPE), so too has TBL research expanded. However, to date, the literature lacks an overview of TBL research that could inform research and education practice by identifying patterns of scholarship productivity over time. To address this gap, the current study aimed to explore the temporal, geographical and intellectual influences within TBL in HPE research.

**Methods:**

Using a bibliometric approach, we investigated the geographical distribution of TBL research, the main influencers in TBL research over the last two decades, and the characteristics of the most cited and co-cited articles. Rogers’ Diffusion of Innovation Theory was employed as a heuristic framework to map and interpret the global trajectory of TBL research.

**Results:**

TBL has been widely adopted in the USA, followed by the Western Pacific and the Eastern Mediterranean. The number of outputs suggests less traction in other global regions. Generally, TBL research is characterised by many one-off publications in lower-impact journals. Only 5% of papers were cited 50 times or more. The scholars who first brought TBL into HPE continue to collaborate and have a significant impact, as evidenced by citations and productivity.

**Conclusions:**

Our findings indicate that TBL in HPE research has been driven by a group of early adopters. However, while TBL seems to be widely adopted, there is a relative lack of cumulative research on this topic. More qualitative work and collaboration sare needed to explicitly examine what drives the adoption and normalisation of TBL and new pedagogies in HPE globally, as well as associated research.

## Introduction

In the last 20 years or so, team-based learning (TBL) has gained popularity as a teaching method in various health profession education (HPE) fields, including medicine [1], nursing [2], physiotherapy [3], pharmacy [4], dentistry [5] and midwifery [6]. TBL follows a highly structured sequence that includes pre-class preparation, small teams working together over time, testing “readiness” at the individual and group levels with immediate feedback on performance, application exercises with class discussion, and peer evaluation of teamwork [7].

TBL was originally conceived in an American business school in the 1970s. It was introduced to the academic community in the early 2000s via a book by Michaelsen [7] and several academic papers reporting on the implementation of TBL in a consortium of 10 medical schools in the USA [7,8,9]. A guidebook introducing TBL specifically to HPE published in 2008 [7]. As TBL gained traction in HPE so too did TBL research. Reviews of the TBL literature indicate that many studies have focused on the mechanisms and outcomes of TBL, usually on one of four topics: effectiveness [10], TBL structure and the quality of TBL reporting [2,11,12], types of outcomes and topics [13,14,15,16], and specific teaching and learning activities such as improving problem-solving abilities and critical thinking [17] or blending TBL with technology [18].

However, the literature lacks an overview of TBL research over time that identifies patterns in scholarship productivity. This is problematic: after 25 years of TBL in health professions education and the increasing number of TBL papers published in HPE (see Results section), it is appropriate to map where TBL research has been and consider where it might go in the future to inform research and educational practice. By mapping how TBL research has spread selectively across regions and institutions, we hope to prompt reflection among decision-makers—both those who have already implemented TBL and those considering it—on the rationale for adopting instructional methods into medical school curricula, particularly those perceived as innovative or trending. Our aim is to support more deliberate and evidence-informed decisions by providing a clearer overview of the global research landscape. A detailed overview of TBL research will also help those who wish to conduct TBL research, those who are considering publishing such work, and those who provide funding for these efforts.

Our analysis was guided by the following research questions:

- What were the main influencers in HPE TBL research in the last two decades, including the most represented countries, journals, authors and their affiliations?
- What are the geographical trends in TBL research?
- What are the characteristics of the most cited and co-cited articles?

We use bibliometric analysis [19], a quantitative narrative that presents evidence on the impact, attention, dissemination, and influence of scientific findings, to map the academic landscape of TBL. Bibliometric studies are becoming increasingly evident in HPE; for example, mapping the landscape of knowledge syntheses in HPE [20], examined e-learning in health [21], reporting on the nations contributing most prolifically to medical education scholarship [22], evaluating where (what journals) different types of HPE research are published [23,24], and analysing the evolution of Problem-Based Learning (PBL) research [25]. The bibliometric analysis reported in this paper aims to map the landscape of TBL research over 20 years in HPE. We examine the origins of studies (authors, affiliations, countries), the venues (journals) in which they are published, the citation rates they receive, and the attention they garner.

Our research questions are well suited for a bibliometric review, which examines the entire body of literature within a field without delving into detailed synthesis of individual findings [26,27].

## Methods

### Study design

Among the full spectrum of bibliometric techniques [27], we used selected performance analysis techniques that “examine the contributions of research constituents to a given field” [27] and science-mapping techniques that “examine the relationships between research constituents” [27]. We specifically employed publication-related metrics (total publications, productivity per active year of publication), citation-related metrics (total citations), citation-and-publication-related metrics (number of cited publications), citation analysis (relationships among publications and the most influential publications), and co-citation analysis (the most influential publications). We followed the guideline for reporting bibliometric reviews of the biomedical literature (BIBLIO) [28] (see supplementary materials).

### Search strategy and data collection procedure

We used Web of Science Core Collection (WoS) to collect relevant data due to its extensive coverage of the scientific field of study and to reduce complications associated with merging data from different databases. The search string included the term “team-based learning” and health professions following WHO classification [29]: (“team-based learning” OR “team based learning”) AND (medic* OR nurs* OR dentist* OR pharmac* OR therap* OR optometr* OR “occupational health” OR paramed* OR midwife* OR “occupational med*” OR dietic* OR nutrition* OR audiologist*). After conducting pilot searches and refining the search strategy (e.g., excluding the “TBL” acronym is important: a search including the acronym yields 25% more records, 91% of which are irrelevant, i.e. referring to other topics such as technology-/task-based learning, trachea/bronchus/lung, triple bottom line, etc.), the search string was used on abstract, title or author keywords on the 15^th^ August 2024. We limited the search to the articles only, no other selection criteria were used. The search and filtering yielded 668 results.

**Figure 1:**
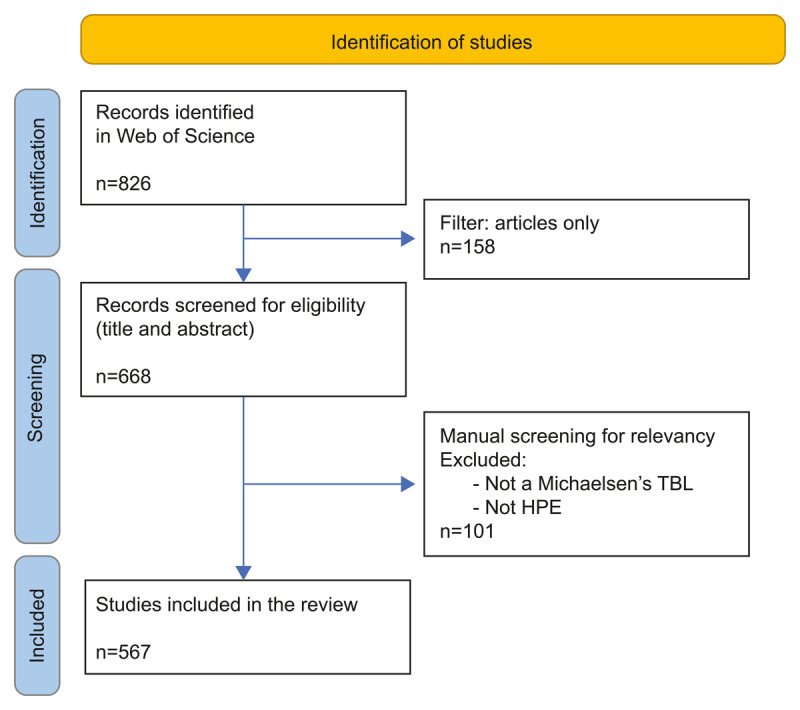
PRISMA Flow chart for bibliometric review on TBL research in health professions education.

We downloaded three datasets: a dataset with full records to analyse global trends, affiliations, journals, and collaborations; a citation report; and a separate dataset with all cited references. Unlike typical bibliometric analyses, which almost never include title and abstract screening for relevance, we manually reviewed titles and abstracts to ensure an accurate account of the spread of TBL. One author (AP) independently screened all titles/abstract. Approximately 15% of abstracts were also independently screened by either JC or SK. Any discrepancies were considered by the team and resolved through discussion. The exclusion criteria included records that either did not refer to TBL as originally described by Michaelsen [7] or did not specifically pertain to HPE. 101 records were excluded, leaving a final dataset of 567 records (see Figure 1). We cleaned the data manually and assigned our own labels in MS Excel (Microsoft) for higher precision than using WoS bibliometric tools for parsing data on authors, institutions, and countries.

### Data analysis and synthesis

The extracted data was tabulated in an MS Excel (Microsoft) spreadsheet. We assigned each paper to its focal health profession (e.g., medicine, nursing). For global trends, we created a line chart in MS Excel of the temporal frequency of publications and calculated the annual growth rate [30]. To analyse temporal and spatial distribution of countries and regions, we manually assigned countries to each record. As some papers reported collaborations across countries, we used whole-counting for countries (hence 631 country attributions for 567 papers). We followed the WHO region classification to assign each country to a global region, but added an additional distinction between the north, central, and south parts of the Americas to show the expansion of TBL in more detail. We used MS Excel to create a map and a line chart to demonstrate the results for the countries. Finally, to better understand the type of journals which publish most TBL papers, we looked at the Journal Impact Factor (from Journal Citation Reports [Clarivate], JCR JIF) of the most popular journals. We used the 2023 IF for journals with the last publication in 2024; otherwise, we used the IF corresponding to the last year of publication.

To analyse affiliations, we cleaned and parsed the data manually in two ways: 1) by assigning the accurate affiliation and number of affiliations to each study manually (as the WoS data was inconsistent in correctness), and 2) by deleting details of departments and schools to focus instead on university name only. We manually analysed author affiliations and collaboration status into sole authorship, collaboration within the institution, collaboration within the country and international collaboration with specific country combinations. Of note, due to the high number of articles found in the USA, we created within-state and across-state divisions. We used Adobe Illustrator (Adobe) to manually build a map of the earliest collaborations, including timestamps.

We parsed, grouped and cleaned the data in MS Excel to identify the most published authors (i.e., those with the highest number of publications). For citation and co-citation analysis, we used VOSviewer (version 1.6.20), a software tool for constructing and visualising scientific landscapes. Similar to Zhang et al. [25], we put a 50 citations minimum threshold for citations (unit of analysis: documents, full counting – preset method, verification of selected documents: manual removal of studies identified for exclusion) and cited reference (co-citation analysis: unit of analysis: cited references, method: full counting) visualisation to focus on the core impactful papers and avoid clutter in the visualisation. The tables of the most cited and co-cited papers (above 50) are included in the appendix. The links and relatedness of citations is determined if they cite each other; whereas the links and relatedness in co-citation is determined based on the number of times the items are cited together. We used Adobe Illustrator (Adobe) to build a map that included the most published authors, and the most cited and co-cited papers to visualise the concentration of influence in the field.

We drew upon Rogers’ Diffusion of innovations (DOI) theory as a heuristic framework to map and interpret the global trajectory of TBL research. According to the DOI theory, innovation typically spreads through a system in stages: beginning with a small group of innovators, followed by early adopters who validate the innovation’s value and drive its broader acceptance. As evidence of effectiveness accumulates, the early majority is influenced to adopt the innovation, eventually leading to uptake by the late majority and, finally, the conservatives and laggards—those most resistant to change [31].

### Ethical approval

This study reports a secondary analysis of publicly available data, so it did not require ethical approval.

## Results

### Global trends

The first paper on TBL in HPE was published in 2005. The median number of annual publications since this time was 27.5. There was a big difference in the number of publications by year. From a slow start, TBL research really began to gain momentum in 2012. The period between 2019 and 2022 was the most productive, with 58–62 publications per year. The number of publications has declined since this time (Supplementary materials: Overview).

The majority of TBL publications were from medicine (57.3% [325/567]), followed by pharmacy (21.9% [124/567]), nursing (15.2% [86/567]), interprofessional education (2.8% [16/567]), dentistry (1.8% [10/567]), occupational medicine (0.7% [4/567]) and midwifery (0.4% [2/567]) (Supplementary materials: Overview).

### Distribution of countries and regions

**Figure 2:**
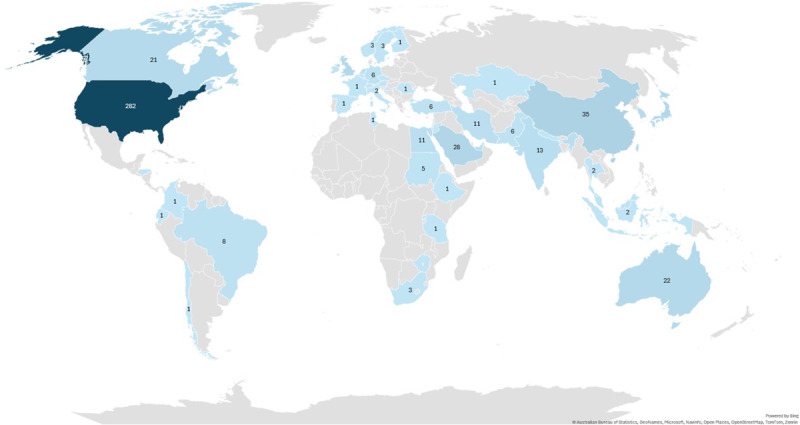
Number of TBL research papers published per country.

Fifty-six countries are represented in the published literature (631 records, see Figure 2 and Supplementary materials: Country distribution). Of these, 23 (41%) countries have only one record (i.e., one publication).

In terms of output, the United States contributed the most TBL publications (282/631 [44.6%]), followed by China (35/631 [5.5%]), Saudi Arabia (28/631 [4.4%]), Japan (24/631 [3.8%]), UK (23/631 [3.6%]), Australia (22/631 [3.4%]) and Canada (21/631[3.3%]). The regions with the highest number of publications were: North America (USA and Canada together, with 48% [303/631] of all the publications), Western Pacific (129/631 [20.4%]), Eastern Mediterranean (88/631 [13.9%]) and Europe (69/631 [10.9%]).

The first published TBL papers in HPE came from North America. These were generated by a small team of colleagues working with the architect of TBL, Larry Michaelsen, to “test” the use of TBL within medical education, first in a single institution (Baylor College of Medicine), then in a collaboration of 10 US institutions that jointly applied for a grant to implement TBL [7]. The outputs of this collaboration included a report on initial experiences [8], a two-year implementation review [9], and the publication of a guide to using TBL in HPE [7]. The colleagues involved in this early 10-centre Team-Based Learning Collaborative and their later collaborators also have some of the most cited papers in the field (see later). Two of the earliest studies in our dataset, published in 2005, were co-authored by educators affiliated with this collaborative [32,33].

Researchers from Europe and the Western Pacific Region were the first to publish on this topic outside the US in 2008. In Europe, Germany first published on TBL but the UK (23) and the Netherlands (11) lead on quantity of publications. In the Eastern Mediterranean region, the United Arab Emirates (UAE) and Lebanon were the first to publish, but Saudi Arabia leads the region in terms of the number of publications (28). In the Western Pacific, South Korea was the first country to publish, but China has been the most productive country (35), followed by Japan (24) and Australia (22).

### Distribution of journals

The identified studies were published in 189 journals (Supplementary materials: Journals). Of these, 44 (44/567 [7.7%]) of papers were published in BMC Medical Education, 43 (43/567 [7.5%]) in Currents in Pharmacy Teaching and Learning, 37 (37/567 [6.5%]) in American Journal of Pharmaceutical Education and 36 (36/567 [6.3%]) in Medical Science Educator. The median impact factor of the eight most common journals in which the identified studies were published was 3.45, and the average was 3.27, ranging from 1.3 to 5.2.

### Distribution of affiliations

A total of 667 affiliated institutions were represented in the 567 papers (Supplementary materials: Affiliations). Of these 667 affiliated institutions, 284 institutions appear only once in the literature. Among them (all USA unless otherwise stated), the University of Sydney, Australia, contributed the most (59 authors in various publications) followed by University of Michigan (45), Wright State University (43), University of Bisha, Saudi Arabia (37), Sun Yat Sen University, China (34), Rowan University (34), Nanyang Technological University, Singapore (33), American University of Beirut, Lebanon (30) and Cedarville University (30). Of the top 20 affiliations by publications, eight were not included in the THE world ranking. The relatively high number of affiliations can be at least partially explained by papers with a high number of co-authors. For example, three of seven publications from the University of Bisha included more than nine co-authors.

### Analysis of authors

In terms of publication volume, the most published authors were based in the USA and Australia, (Supplementary materials: Authors). Annette Burgess from the University of Sydney published the most studies on TBL: 11/567 (1.9%). The following four most published authors were from institutions in the original USA Team-based Learning Collaborative. They were Kathryn Behling (9/567), Osvaldo Lopez (9) and Gonzalo Carrasco (8), all from Rowan University, and Dean Parmelee (8) from Wright State University [7,8,9].

Forty-four studies (7.8%) were authored by a single individual. The most common number of authors per paper was three (113/567 [19.9%]), followed by four (103/567 [18.1%]). At the other end of the spectrum, there were single studies featuring 14, 18, and even 26 co-authors. In total, 2,103 authors were represented across the 567 publications, with 1,875 appearing only once (i.e., 89% of authors contributed to a single TBL publication only).

### Collaborations

Of the 567 studies, 296 (52.2%) were conducted within a single institution, although mostly involving multiple departments (252/296 [85.1%]; Supplementary materials: Collaborations). A further 271 of 567 (47.8%) studies represented collaboration across institutions. The most common type of inter-institutional collaboration was between institutions within the same country (213/271 [78.6%]), rather than international collaborations (58/271 [21.4%]). Of these, the largest number (118/213) were collaborations within the USA: 39.9% (47/118) involved within-state collaboration and 60.2% (71/118) involved collaboration across multiple states.

There were 40 different combinations of international collaborations. The US was the most active country in respect to international collaborations: 27 of the 58 international collaborations were from the USA. The other countries which were most often represented in international collaborations were Saudi Arabia (7/40 combinations, five with Egypt and two with Sudan), UAE (6/40), the UK (5/40), Canada (4/40), China (4/40) and the Netherlands (4/40).

Five publications reported collaborations across three countries. Eastern Mediterranean countries were heavily represented in these three-country collaborations (e.g., Bahrain, Saudi Arabia and Sudan; Egypt, Sudan and Saudi Arabia; Bahrain, Ireland and UAE [with this last representing a collaboration across three of the Royal College of Surgeons of Ireland campuses]; UAE, USA and Jordan, and Netherlands, Norway and Sweden). Notably, all but one of the three-way collaborations included at least one of the Gulf countries. 31 collaborations were observed only once.

### Citations and co-citations

At the time of this analysis, 28 of the included studies had more than 50 citations, with 9 cited more than 100 times (Supplementary materials: Citations). Koles et al.’s 2010 publication on the impact of TBL on academic performance [34] was the only paper cited more than 200 times. There were 11,528 cited references, 68 of which met our minimum of 20 citations of a cited reference threshold (Supplementary materials: Co-citations). Only two papers were *co*-cited more than 100 times: Parmelee et al.’s guide on the fundamentals of TBL [35] and Koles et al.’s research on TBL impact on academic performance, co-authored by Parmelee [34]. In short, the output from Koles, one of the original US TBL Collaboration, was both the most cited and the second most frequently co-cited work. Six of the nine (indicated by over 100 citations per paper) and 12 out of 17 (indicated by over 50 co-citations per paper) most cited publications are from members of the original US TBL Collaboration. It is relevant here to acknowledge the effect of time – older publications have had more opportunity to accumulate citations.

### Groups of adopters

**Figure 3:**
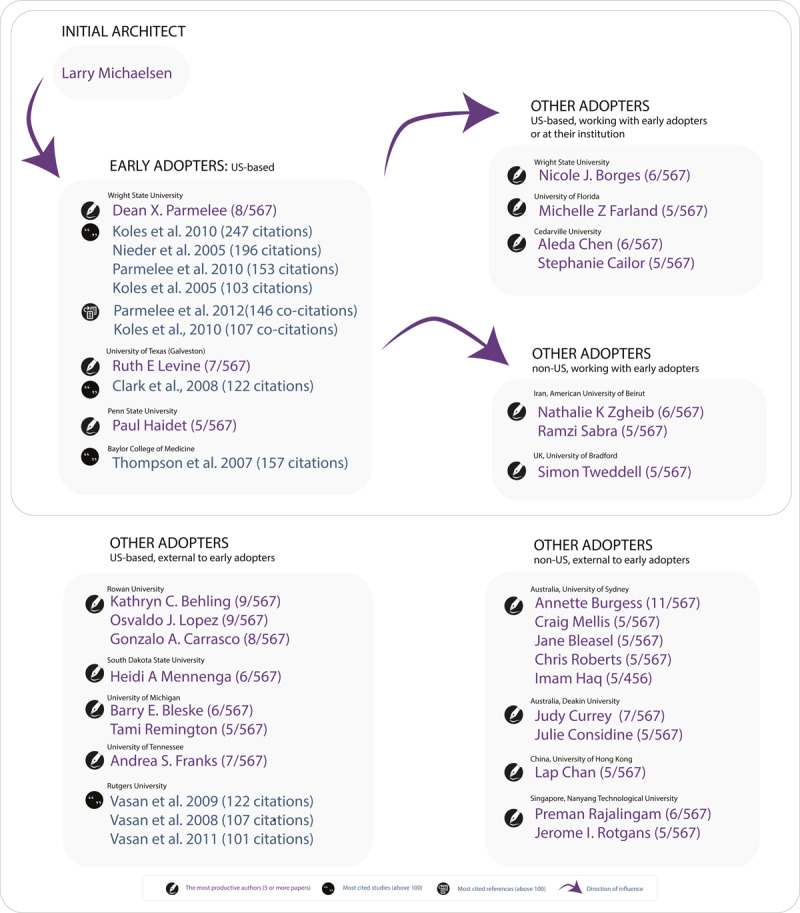
Mapping of TBL influencers: most productive authors, most cited studies and most co-cited references.

Figure 3 maps the most productive authors, top-cited papers, and co-citation together to visualise groupings. It reveals distinct patterns of scholarly connection and influence. Two notable categories of adopters emerge: early and other. The early adopters—comprising members of the original TBL collaborative and/or authors of the TBL guide for HPE (i.e., the first 10 institutions that implemented TBL in medical education) — account for the majority of highly cited publications [9,32,33,34,36,37] and co-citations [34,35]. These individuals and institutions played a foundational role in shaping the field and influencing subsequent adopters, both within and beyond the US.

The second category, “Other,” includes authors who were not part of the original TBL collaborative. In this group, highly cited papers are less common [38,39,40], even though the number of research-productive authors is notably higher. This suggests a broader diffusion of research activity.

## Discussion

In this bibliometric analysis, we set out to identify the main influencers in HPE TBL research over the last two decades, the geographical trends in the spread of TBL research, and the characteristics of the most cited and co-cited articles. We identified that after a slow start between 2002 and 2010, TBL publications rapidly increased and stayed high from 2011 to 2022, declining slightly recently. Medicine dominated the field in terms of the number of publications, followed by pharmacy and nursing. TBL research was conducted across 56 countries, with North America leading in the number of publications and collaborations, followed by the Western Pacific, the Eastern Mediterranean, and Europe. A very large proportion of the institutions and authors represented in the TBL literature have published only one paper on this topic, and most publications have appeared in lower-impact journals.

In the discussion that follows, we draw on Rogers’ terminology to further analyse the characteristics of TBL research production.

### Concentration of Influence

DOI indicates that ideas are more effectively transferred between individuals who are similar, or homophilous [31]. Viewed in this interpretative light, the original, early adopters of TBL within HPE were similar in that they were all based in the same country and system (the USA). They were highly interconnected, as evidenced by the formal 10-institution collaborative. These similarities and connections are likely to underpin their high-impact publishing on the topic – seven of the 10 most-cited papers (those with over 100 citations) come from this initial group. These early adopters have continued to work together over time, positioning them as an “invisible college”, a set of interacting scholars or scientists who share similar research interests and work together to grow knowledge and influence on a topic [41,42]. A similar country concentration was identified in the diffusion of the use of simulation in medical education by Ba et al. [43]. However, unlike our analysis of TBL, Ba and colleagues’ study does not trace the diffusion back to specific early adopters linked to the primary architect of the innovation.

Interestingly, the patterns of TBL publication and collaboration observed in other countries differ from those in the USA. Specifically, outside the USA, TBL research seems to be spread across a wider, more diverse (or heterophilous to use Rogers’ language) range of authors, contrasting with the more concentrated (homophilous) influence of key figures in the US. This diversity of voices suggests a different dynamic to the diffusion of TBL, but the list of the most cited studies suggests otherwise.

In terms of DOI, early adopters tend to interact primarily within their own circles [40], spreading innovation within a limited network initially, then more widely with the help of other adopters (this proposal is supported by the repetition of names in Supplementary tables 8 and 9). At the outset, the adoption rate is low, but it then increases gradually (as more people use it, others observe its use, and if the innovation is better than what went previously, others start to adopt and use it) and decreases to the point of saturation [41]. Overall, TBL’s productivity overtime seems to align with the diffusion curve (see Supplementary materials: Supplementary figure 1), but the homophilous concentration of influence persists and may even have served as an implicit barrier to broader diffusion [31]. Further examination of the literature is required to determine whether there are differences in the nature of TBL research produced by scholars within and outside the core USA collaborative group.

### The dominance of “one-off” publications

DOI theory suggests several possible reasons which could explain the pattern of “one-off” publishing we observed. The dominance and concentration of influence among early adopters may have inadvertently discouraged broader engagement from new researchers. Alternatively, the plethora of “one-off” studies may thus be due to the need to confirm [31] the acceptability and effectiveness of TBL locally. Once these are confirmed, TBL may have shifted into the routinisation phase of innovation adoption [44] when no further confirmation in the specific implementation environment is desired.

Interestingly, the DOI theory differentiates between horizontal and vertical diffusion, with the latter associated with improvements in quality. The nature of our review did not enable analysis of the quality of the papers or of whether the studies were knowledge-building [45]. However, the fact that most TBL papers are published in lower-impact journals suggests that the quality of research on this topic may be limited. This becomes evident when compared with PBL research, which is largely driven by a small group of research-intensive, relatively high-ranking universities (consistently placed within the global top 200) and published in high-impact journals [25]. The question of research characteristics requires further investigation.

### Future Directions

The findings of this bibliometric analysis leave some questions unanswered, such as: What drove the adoption of, and enthusiasm for, TBL in HPE in the first place? As per Kuhn [46], textbooks (in this case, the first TBL textbook) tend to follow rather than produce revolution. Given this, it would seem prudent to identify and examine the political, economic, cultural and social dynamics and trends in US higher and health professions education in the late 1990s and early 2000s, which provided the context for the development and adoption of TBL. Also, why have some global regions (notably Asia, the Middle East and the USA) seemed to embrace TBL – or to be more accurate, publish on TBL – more than others (Europe, Africa, Central and South America)? Drawing on DOI theory again, TBL adoption is likely related to its relative advantage (how much better TBL is perceived to be compared to its predecessor), compatibility (how well TBL aligns with the existing values, past experiences, and needs of the potential adopters) and its complexity (the perceived difficulty of understanding, implementing and using TBL). For example, cultural and/or resource barriers may explain the low adoption of TBL in Africa and its relatively high adoption in Gulf States.

To explore the relative advantage, compatibility, and complexity of TBL in different places requires a socio-historical gaze and appropriate qualitative approaches, such as discourse analysis and/or comparative case studies, to shed light on why TBL’s popularity varies across countries and regions. The insights from such qualitative work could also provide useful knowledge to help future potential adopters of TBL make informed decisions about TBL’s suitability for their local contexts. This in turn will help in terms of targeting investment in change.

As mentioned earlier, TBL research continues to be dominated by outputs from the original collaboration group from the USA. Echoing Oluwadele et al.’s evaluation of trends in e-learning [47] and He et al.’s analysis of the inverted classroom in medical education [48], it would be good to see more collaborations represented in TBL research, particularly collaborations across different cultures and contexts. Widening the nature of TBL collaborations may support more programmatic and context-sensitive research, and potentially more decentralised diffusion of this instructional method.

#### Strengths and limitations

Bibliometric analysis in isolation examines only high-level productivity and science mapping as it usually deals with a high volume of data. Supplementing this approach with a more narrative synthesis allowed us to better capture the complexities of real-world research influences/rs.

Bibliometric data can vary depending on the database used. This means that the data, and consequently, the conclusions, drawn from this analysis could differ if other sources, such as Scopus or OpenAlex (an alternative to proprietary bibliometric tools like WoS or Scopus), were used or if searches from multiple databases were combined. These differences may affect results and interpretations. However, Lopez-Illescas et al. found that oncological journals indexed in Scopus but not in WoS are largely nationally oriented and currently play a peripheral role in international scholarly communication [49]. Additionally, the merging of search results from multiple databases, while promising for broader retrieval and reducing the underrepresentation of research in languages other than English, introduces substantial challenges related to data noise and complexity [50,51]. Lastly, because of the search date, the dataset does not reflect a complete picture of research production in 2024.

We used bibliometric tools in a focused way. We chose not to assess authors’ h-indices as the h-index reflects the overall citation count across an author’s entire body of work. Rather than conducting co-citation analyses of institutions, journals, and authors, we focused on identifying the most frequently co-cited papers to highlight influential researchers and papers. Journal Impact Factor (JCR JIF) has been widely criticised for issues such as asymmetry between the numerator and denominator, journal self-citations, differences across disciplines, and so on [52]. However, alternative measures such as Eigenfactor Metrics or Source Normalised Impact per Paper would not have allowed us to compare our data with existing studies, such as Zhang et al.’s work on PBL [25], so we took the pragmatic decision to use JCR JIF for this study.

Finally, we did not engage with Rogers’ framework of early adopters, early majority, late majority and laggards in our analysis. Our rationale was that the global context of health professions education, with its diverse professional groups and cultures, is highly heterogeneous and does not lend itself to simple categorisations originally developed for change within discrete industries. As per Bordenave [53] and Cook, Satizábal, and Curnow [54], we have used a broader set of questions to understand the adoption of TBL and have called for more research examining the socio-political factors driving this and other changes in HPE. As such, we deemed using Rogers’ DOI as a heuristic device as opposed to a rigidly categorised explanatory framework as more fit-for-purpose.

## Conclusion

Despite its popularity in practice, TBL research seems to be characterised by a concentration of influence and one-off outputs clustered in journals with modest JIFs, compared to research on other commonly used pedagogies. Perhaps related to this, the diffusion —or reports of TBL diffusion —appears to be slowing down. Further research explicitly examining what drives the adoption and normalisation of TBL and other new pedagogies in HPE, as well as what drives associated research, is critical to ensure future curricular change is fit-for-purpose and educationally robust.

## Data Accessibility Statement

Data are available on request. Researchers who wish to obtain further details may contact the corresponding author.

## Additional File

The additional file for this article can be found as follows:

10.5334/pme.1874.s1 Supplementary Materials. Supplementary materials 1, 2 and 3.
