# Supplementary material for: Domination of Early Adopters: A Bibliometric Review of Team-Based Learning Research in Health Professions Education 2005–2024
Source: Perspect Med Educ. 2025 Dec 4;14(1):915–26. doi: 10.5334/pme.1874 (PMC12680001; doi:10.5334/pme.1874)
Supplement: Supplementary Materials. — Supplementary materials 1, 2 and 3. [file pme-14-1-1874-s1.pdf]

Supplementary materials

Supplementary materials 1 – BIBLIO report
Supplementary materials 2 – Additional tables and figures

- Overview
- Country distribution
- Journals
- Affiliations
- Authors
- Collaborations
- Citations
- Co-Citations

Supplementary materials 3 – Evidence table

Supplementary materials 1 – BIBLIO

Reporting guideline for reporting bibliometric reviews of the biomedical literature (BIBLIO)

Section/topic	Item no	Checklist item	Reported on page no
**Title**			
Identification	1	Identify the report as a bibliometric review in the title	Title page
Issues/topics	2	Indicate the key issues/topics under investigation and coverage of time period	Title page
**Abstract**			
Structured summary	3	Structured summary including (as applicable): background, methods, results (key findings), and conclusions	Abstract
**Introduction/background**			
Justification/rationale/explanation	4	Present review of existing knowledge and epidemiological information	Introduction
Objectives	5	Statement of the objective(s) or question(s)	Introduction
**Methods**			
Search engines (data sources)	6	Describe all information sources (such as electronic databases, contact with study authors, trial registers, or other gray literature sources)	Methods>"Search strategy and data collection procedure"
Search strategy	7	Keywords and systematization criteria (date of search, language, type of document) for the search	Methods>"Search strategy and data collection procedure"
Time period	8	The period that the review covers and the justification	Methods>"Search strategy and data collection procedure"
Eligibility criteria	9	Describe all inclusion and exclusion criteria, languages, study design, type of publication, and time period	Methods>"Search strategy and data collection procedure"
Data refinement (data selection procedure)	10	Remove the irrelevant articles; inspection to eliminate duplicate and unrelated articles (after evaluation of the title, abstract, and content)	Methods>"Search strategy and data collection procedure" & "Data analysis and synthesis"
Quality assessment (optional)	11	Assessment of papers by three authors and the use of assessing checklists	N/A
Data synthesis	12	Describe the methods used for summarizing, handling, synthesis, tabulations, or schematic displays. Describe how the data were analyzed	Methods> "Data analysis and synthesis"
**Results**			
Descriptive findings (statistics)	13	- Provide details of the search and selection process in a flow diagram<br>- Number of citations retrieved (number of publication, year of publication, type of documents, country of publication, articles with the highest impact, most impactful authors, most impactful articles, authors with the highest production, top journals, top institutions, …)	Results
Schematic map and trend	14	Summarize and/or present the schematic maps and trends using an appropriate software to present citations, journals, authors, top journals, time trends, emerging literature, and any relevant indicators (as applicable) [64,65,66,67,68]	Results
Tabulation and summarizing the findings	15	General recommendation: Studies under consideration could be summarized and organized by different subtitles and different scenarios. Regardless, results need to be presented in separate tables covering each subtitle. The followings are some options that could help to summarize the findings<br>Option 1:<br>- Start the presentation with a historical view [when and who first published on the topic]<br>- Report on review papers. The result should be listed in a separate table. Also, specify the review type (scoping review, narrative review, systematic review, and meta-analysis)<br>- Summarize the findings according to the study designs and main study types<br>Option 2:<br>- Start the presentation with a historical view [when and who first published on the topic]<br>- Report on review papers. The result should be listed in a separate table. Also, indicate the review type (scoping review, narrative review, systematic review, and meta-analysis) should be specified<br>- Summarize the findings according to outcome measures or populations. For example, see [63]<br>Option 3:<br>- Start the presentation with a historical view [when and who first published on the topic]<br>- Report on review papers. The result should be listed in a separate table. Also, specify the review type (scoping review, narrative review, systematic review, and meta-analysis)<br>- Summarize the findings according to concept [28]<br>Option 4:<br>- Start the presentation with a historical view [when and who first published on the topic]<br>- Report on review papers. The result should be listed in a	Results<br>Supplementary materials

		separate table, and also specify the review type (scoping review, narrative review, systematic review, and meta-analysis)<br>- Summarize the findings according to different subtitles relevant to the main topic [26]	
Synthesis of findings	16	Synthesize the findings as much as possible, find the gap, and propose a model, hypothesis, etc. (if applicable)	Results
**Discussion**			
Summary of evidence	17	Summarize the main findings. The findings should be presented in more “general” or “accessible” terms	Discussion
Interpretation	18	Include interpretation consistent with results. Explanations for observed outcomes, similarities, and differences reported would be essential	Discussion
Strengths and limitations	19	Discuss the strengths and limitations	Discussion>Strengths and limitations
Conclusion(s)	20	Provide a general interpretation of the results with respect to the review questions and objectives, as well as potential implications	Discussion>Conclusion

Supplementary materials 2 – Additional tables and figures

Overview

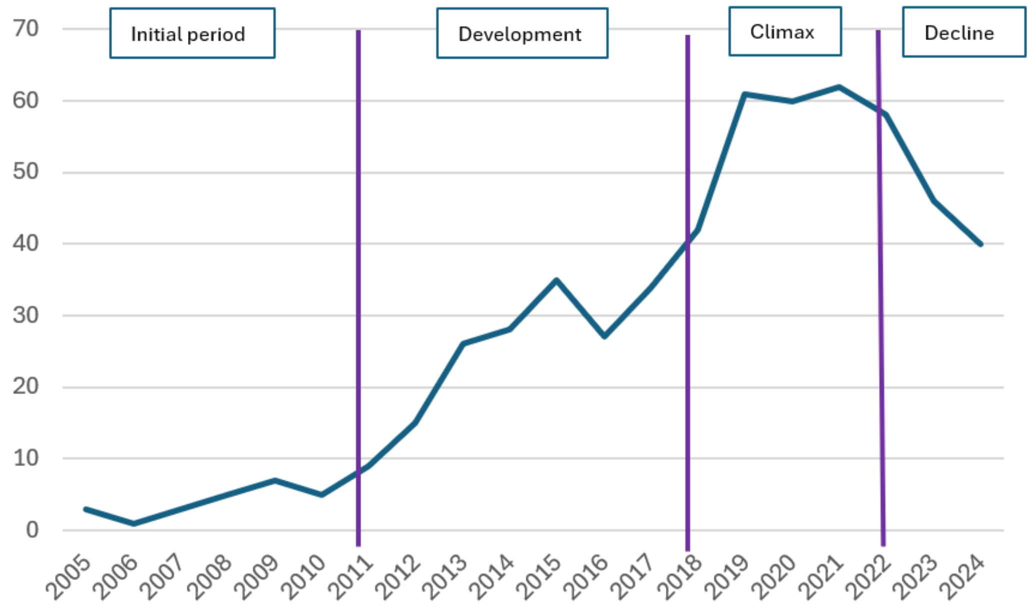

Figure 1 Frequency of TBL research papers over time.

Year	no of publications	% of publications	Cumulative publications	% of cumulative publications	Annual growth rate	Productivity phase
2005	3	0.53	3	0.53	0	Initial period
2006	1	0.18	4	0.71	25.00	
2007	3	0.53	7	1.23	42.86	
2008	5	0.88	12	2.12	41.67	
2009	7	1.23	19	3.35	36.84	
2010	5	0.88	24	4.23	20.83	
2011	9	1.59	33	5.82	27.27	
2012	15	2.65	48	8.47	31.25	Development
2013	26	4.59	74	13.05	35.14	
2014	28	4.94	102	17.99	27.45	
2015	35	6.17	137	24.16	25.55	
2016	27	4.76	164	28.92	16.46	
2017	34	6.00	198	34.92	17.17	
2018	42	7.41	240	42.33	17.50	
2019	61	10.76	301	53.09	20.27	Climax
2020	60	10.58	361	63.67	16.62	
2021	62	10.93	423	74.60	14.66	
2022	58	10.23	481	84.83	12.06	
2023	46	8.11	527	92.95	8.73	Decline
2024	40	7.05	567	100.00	7.05	
Total	567<br>28.35 (mean)<br>27.5 (median)				22.22 (mean)	

Table 1 Yearly distribution and growth of publications in HPE TBL

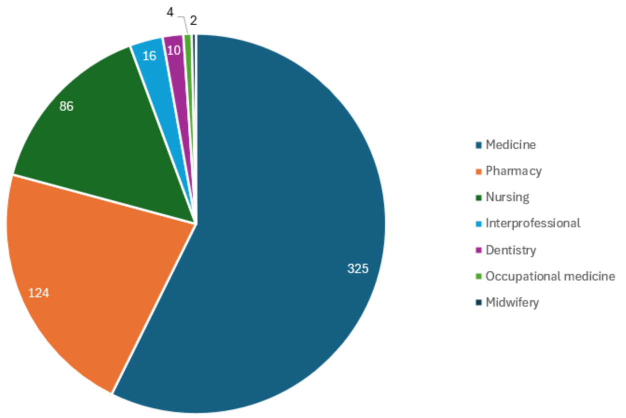

Figure 2 Health professions education.

Country distribution

Country	Record Count	% of 631
Australia	22	3.5
Austria	1	0.2
Bahrain	3	0.5
Barbados	1	0.2
Belgium	1	0.2
Brazil	8	1.3
Canada	21	3.3
Chile	1	0.2
China	35	5.5
Colombia	1	0.2
Croatia	1	0.2
Czech Republic	1	0.2
Dominica	1	0.2
Ecuador	1	0.2
Egypt	11	1.7
Ethiopia	1	0.2
Finland	1	0.2
France	1	0.2
Germany	6	1.0
Grenada	1	0.2
Honduras	1	0.2
India	13	2.1
Indonesia	2	0.3
Iran	11	1.7
Ireland	3	0.5
Israel	1	0.2
Italy	2	0.3
Japan	24	3.8
Jordan	3	0.5
Kazakhstan	1	0.2
Lebanon	7	1.1
Malaysia	4	0.6
Nepal	1	0.2
Netherlands	11	1.7
Norway	3	0.5
Pakistan	6	1.0
Romania	1	0.2
Rwanda	2	0.3
Saudi Arabia	28	4.4
Singapore	14	2.2
South Africa	3	0.5
South Korea	19	3.0
Spain	1	0.2
Sudan	5	0.8
Sweden	3	0.5
Switzerland	3	0.5
Taiwan	11	1.7
Tanzania	1	0.2
Thailand	2	0.3
Trinidad Tobago	1	0.2
Tunisia	1	0.2
Turkey	6	1.0
UAE	12	1.9
UK	23	3.6

USA	282	44.7
Zimbabwe	1	0.2
Total	631	

Table 2 Number of publications per country

Countries	Region	2005	2006	2007	2008	2009	2010	2011	2012	2013	2014	2015	2016	2017	2018	2019	2020	2021	2022	2023	2024	Total
ETHIOPIA	African Region *Sum of papers: 8 Percentage of 1s: 60%*																			1		1
RWANDA																	1			1		2
SOUTH AFRICA															1	1		1				3
TANZANIA											1											1
ZIMBABWE											1											1
BAHRAIN	Eastern Mediterranean Region *Sum of papers: 88 Percentage of 1s: 18%*																1	1		1		3
EGYPT																1			3	4	3	11
IRAN													1	1	4	3	2					11
ISRAEL												1										1
JORDAN										1							1		1			3
LEBANON							1	1	1			1	1				1		1			7
PAKISTAN											1				1	1		1	2			6
SAUDI ARABIA												1	1	1	2	3	1	8	4	3	4	28
SUDAN																1		2			2	5
TUNISIA																	1					1
UAE							1	1		1					2	1	1	1	2	2		12
AUSTRIA	European Region *Sum of papers: 69 Percentage of 1s: 50%*					1																1
BELGIUM															1							1
CROATIA																		1				1
CZECH REPUBLIC																1						1
FINLAND																			1			1
FRANCE																					1	1
GERMANY					1				1					1	1			2				6
IRELAND											1						1			1		3
ITALY															1		1					2
KAZAKHSTAN															1							1
NETHERLANDS												1			1	1	3		2		3	11
NORWAY																	1				2	3
ROMANIA														1								1
SPAIN																					1	1
SWEDEN																		1			2	3
SWITZERLAND															1			1	1			3
TURKEY											1			1	1	1					2	6
UK										1	1	1	5	1	2	3	2	1	3	1	2	23
BARBADOS	Region of Americas (central) *Sum of papers: 5 Percentage of 1s: 100%*															1						1
DOMINICA									1													1
GRENADA														1								1
HONDURAS																1						1

Countries	Region	2005	2006	2007	2008	2009	2010	2011	2012	2013	2014	2015	2016	2017	2018	2019	2020	2021	2022	2023	2024	Total
TRINIDAD TOBAGO																1						1
CANADA	Region of Americas (north) *Sum of papers: 303 Percentage of 1s: 0%*							2	1	2	2	1	1			2	2	3	3	2		21
USA		3	1	3	3	5	3	4	10	21	14	26	18	21	18	36	21	27	19	15	14	282
BRAZIL	Region of Americas (south) *Sum of papers: 11 Percentage of 1s: 75%*														1		3		1	3		8
CHILE																	1					1
COLOMBIA											1											1
ECUADOR																				1		1
INDIA	South-East Asian Region *Sum of papers: 18 Percentage of 1s: 25%*										1					1	2		3	2	4	13
INDONESIA																			1		1	2
NEPAL																			1			1
THAILAND																			1	1		2
AUSTRALIA	Western Pacific Region *Sum of papers: 129 Percentage of 1s: 0%*								1	1	1	2	1	2	1	2	4	3	1	2	1	22
CHINA							1				2		1	3	2	1	4	5	8	3	5	35
JAPAN									1	2	1	2		2		1	2	4	2	5	2	24
MALAYSIA													1					1		2		4
SINGAPORE								1						1	2	2	4		3	1		14
SOUTH KOREA					1	1					1	1	1		2		4	4	2	2		19
TAIWAN											2					2	1	2	1	2	1	11
Total		3	1	3	5	7	6	9	16	29	31	37	31	36	45	67	65	69	66	55	50	631

Table 3 Number of publications per country, region and year

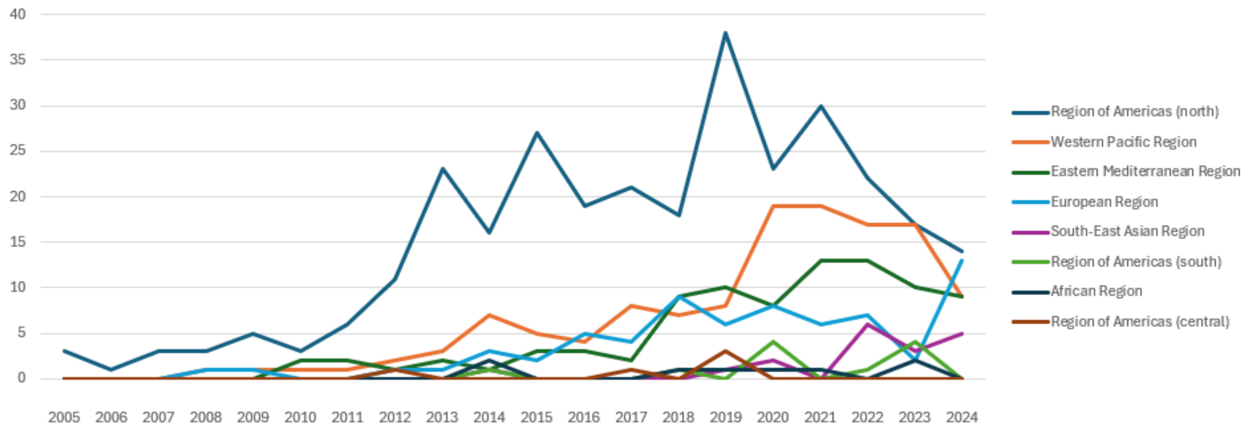

Figure 3 Number of publications per region over time.

Journals

Journal	Total	Publication years	Impact factor from 2023 or the last publication year
BMC Medical Education	44	2011, 2013-2014, 2016--2024	2.7
Currents in Pharmacy Teaching and Learning	43	2011, 2013-2024	1.3
American Journal of Pharmaceutical Education	37	2009, 2011-2019, 2021-2024	3.8
Medical Science Educator	36	2019-2024	1.9
Medical Teacher	25	2009-2012, 2014-2020, 2022	3.3
Nurse Education Today	17	2011, 2013-2014, 2016-2018, 2020-2021, 2023	3.6
Anatomical Sciences Education	14	2008-2009, 2011, 2012, 2014-2016, 2018, 2020-2021, 2024	5.2
Journal of Educational Evaluation for Health Professions	11	2008, 2014-2017, 2019, 2022	4.4
		Median	3.45
		Average	3.27

Table 4 Journals with more than 10 publications

Affiliations

Affiliations	Country	Record Count (authors' affiliation count)	THE ranking 2024
University of Sydney	Australia	59	60
University of Michigan	USA	45	23
Wright State University Dayton	USA	43	N/A
University of Bisha	Saudi Arabia	37	N/A
Sun Yat Sen University	China	34	201-250
Rowan University	USA	34	1001-1200
Nanyang Technological University	Singapore	33	32
American University of Beirut	Lebanon	30	501-600
Cedarville University	USA	30	N/A
University of Florida	USA	29	132
Deakin University	Australia	28	251-300
University of Texas at Tyler	USA	25	N/A
Central South University	China	24	N/A
Tokushima University	Japan	22	1201-1500
University of North Carolina Chapel Hill	USA	21	72
University of Alabama Birmingham	USA	21	N/A
Ohio State University	USA	21	99
California Northstate University	USA	21	N/A
University of Sharjah	USA	20	351-400
St. Luke's International Hospital	Japan	20	N/A

Table 5 Affiliations with more 20 or more records

Authors

Authors	Affiliation	Country	Record Count	First publication on TBL
Annette Burgess	University of Sydney	Australia	11	2012
Osvaldo J. Lopez	Seton Hall University	USA	9	2016
Kathryn C. Behling	Rowan University	USA	9	2016
Dean X. Parmelee	Wright State University	USA	8	2005
Gonzalo A. Carrasco	Rowan University	USA	8	2018
Ruth E. Levine	The University of Texas	USA	7	2007
Judy Currey	Deakin University	Australia	7	2013
Andrea S. Franks	University of Tennessee	USA	7	2013
Nathalie K Zgheib	American University of Beirut	Lebanon	6	2010
Preman Rajalingam	Nanyang Technological University	Singapore	6	2018
Heidi A. Mennenga	South Dakota State University	USA	6	2012
Aleda MH Chen	Cedarville University	USA	6	2015
Barry E. Bleske	University of Michigan	USA	6	2014
Nicole J. Borges	Wright State University	USA	6	2010
Simon Tweddell	University of Bradford	England	5	2016
Ramzi Sabra	American University of Beirut	Lebanon	5	2010
Jerome I Rotgans	Nanyang Technological University, Erasmus Medical School, Karolinska Institute	Singapore, Netherlands, Sweden	5	2018
Tami L Remington	University of Michigan	USA	5	2014
Chris Roberts	University of Sydney	Australia	5	2017
Craig Mellis	University of Sydney	Australia	5	2012
Paul Haidet	Penn State University	USA	5	2006
Michelle Z Farland	University of Florida	USA	5	2015
Inam Haq	University of Sydney	Australia	5	2017
Stephanie Cailor	Cedarville University	USA	5	2015
Jullie Considine	Deakin University	Australia	5	2013
Lap K Chan	University of Hong Kong	China	5	2017
Jane Bleasel	University of Sydney	Australia	5	2017

Table 6 Most prolific authors with 5 and more publications

Collaborations

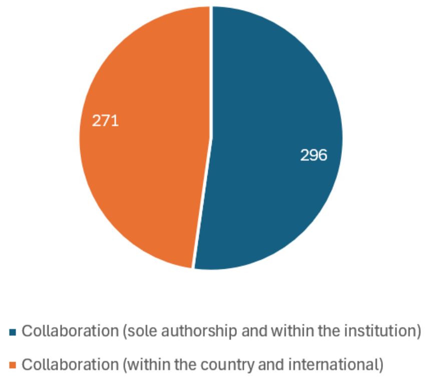

Figure 4 Number of publications versus type of collaboration - overview

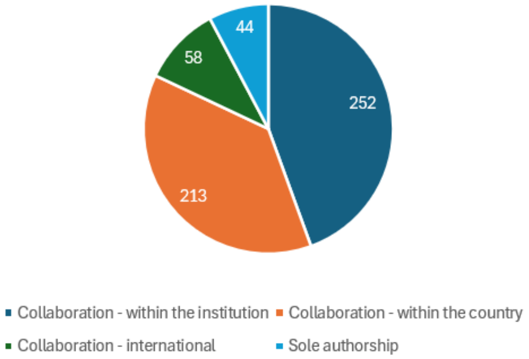

Figure 5 Number of publications versus type of collaboration.

Countries	No. of publications
Egypt+Saudi Arabia	5
Netherlands+Singapore	4
South Korea+USA	4
Iran+USA	3
Japan+USA	3
Saudi Arabia+USA	2
Saudi Arabia+Sudan	2
UK+USA	2
Canada+USA	2
Canada+Rwanda	1
Egypt+Saudi Arabia+Sudan	1
Saudi Arabia+UAE	1
Egypt+USA	1
Malaysia+UAE	1
Germany+Switzerland	1
Norway+Sweden	1
Grenada+USA	1
Canada+Saudi Arabia	1
Honduras+USA	1
UK+Israel	1
Indonesia+Japan	1
Netherlands+Norway+Sweden	1
Bahrain+Saudi Arabia+Sudan	1
Netherlands+USA	1
Italy+Switzerland	1
China+UAE	1
Japan+Netherlands	1
Bahrain+Ireland+UAE	1
USA+Zimbabwe	1
Tanzania+USA	1
USA+China	1
China+Switzerland	1
UK+Canada	1
UK+Jordan	1
Australia+USA	1
UK+UAE	1
Jordan+USA	1
China+Nepal	1
Lebanon+USA	1
Jordan+UAE+USA	1

Table 7 Collaborations between countries

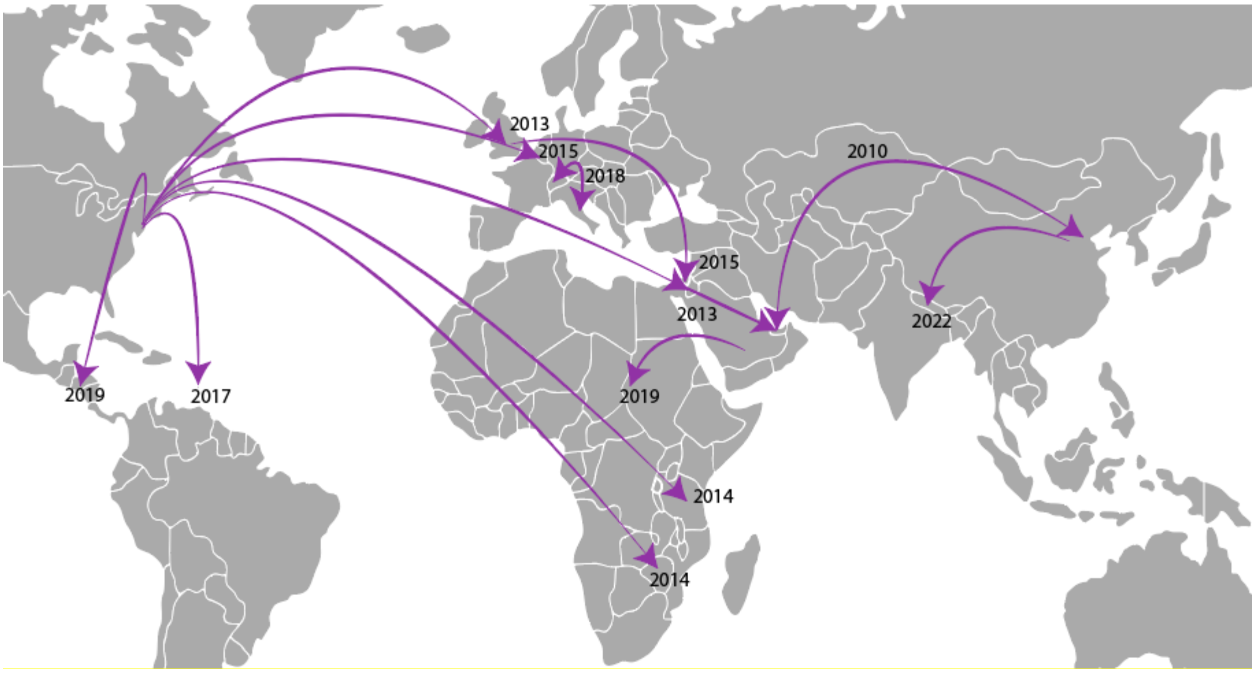

Figure 6 Collaborations that introduced TBL to new countries, i.e. countries where the first paper published on TBL was in collaboration with another country.

Citations

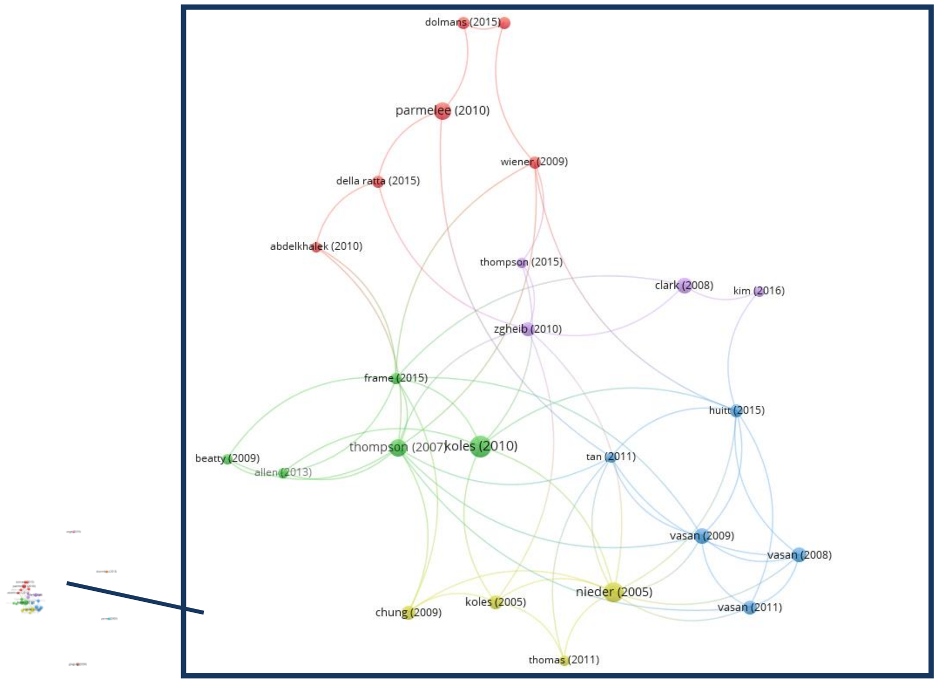

Figure 7 Top research cited more than 50 times

Study (Studies with more than 50 citations)	Publication Year	Total Citations
Koles, P. G., Stolfi, A., Borges, N. J., Nelson, S., & Parmelee, D. X. (2010). The impact of team-based learning on medical students' academic performance. *Academic Medicine*, *85*(11), 1739-1745.	2010	247
Nieder, G. L., Parmelee, D. X., Stolfi, A., & Hudes, P. D. (2005). Team-based learning in a medical gross anatomy and embryology course. *Clinical Anatomy: The Official Journal of the American Association of Clinical Anatomists and the British Association of Clinical Anatomists*, *18*(1), 56-63.	2005	196
Thompson, B. M., Schneider, V. F., Haidet, P., Levine, R. E., McMahon, K. K., Perkowski, L. C., & Richards, B. F. (2007). Team-based learning at ten medical schools: two years later. *Medical education*, *41*(3), 250-257.	2007	157
Parmelee, D. X., & Michaelsen, L. K. (2010). Twelve tips for doing effective team-based learning (TBL). *Medical teacher*, *32*(2), 118-122.	2010	153
Vasan, N. S., DeFouw, D. O., & Compton, S. (2009). A survey of student perceptions of team-based learning in anatomy curriculum: Favorable views unrelated to grades. *Anatomical sciences education*, *2*(4), 150-155.	2009	122
Clark, M. C., Nguyen, H. T., Bray, C., & Levine, R. E. (2008). Team-based learning in an undergraduate nursing course. *Journal of Nursing Education*, *47*(3), 111-117.	2008	122
Vasan, N. S., DeFouw, D. O., & Holland, B. K. (2008). Modified use of team-based learning for effective delivery of medical gross anatomy and embryology. *Anatomical sciences education*, *1*(1), 3-9.	2008	107
Koles, P., Nelson, S., Stolfi, A., Parmelee, D., & DeStephen, D. (2005). Active learning in a year 2 pathology curriculum. *Medical education*, *39*(10), 1045-1055.	2005	103
Vasan, N. S., DeFouw, D. O., & Compton, S. (2011). Team-based learning in anatomy: An efficient, effective, and economical strategy. *Anatomical sciences education*, *4*(6), 333-339.	2011	101
Gregory, J. K., Lachman, N., Camp, C. L., Chen, L. P., & Pawlina, W. (2009). Restructuring a basic science course for core competencies: An example from anatomy teaching. *Medical teacher*, *31*(9), 855-861.	2009	97
Zgheib, N. K., Simaan, J. A., & Sabra, R. (2010). Using team-based learning to teach pharmacology to second year medical students improves student performance. *Medical teacher*, *32*(2), 130-135.	2010	95
Chung, E. K., Rhee, J. A., & Baik, Y. H. (2009). The effect of team-based learning in medical ethics education. *Medical teacher*, *31*(11), 1013-1017.	2009	94
Huitt, T. W., Killins, A., & Brooks, W. S. (2015). Team-based learning in the gross anatomy laboratory improves academic performance and students' attitudes toward teamwork. *Anatomical sciences education*, *8*(2), 95-103.	2015	88
Dolmans, D., Michaelsen, L., Van Merrienboer, J., & Van der Vleuten, C. (2015). Should we choose between problem-based learning and team-based learning? No, combine the best of both worlds. *Medical teacher*, *37*(4), 354-359.	2015	81
Burgess, A., Bleasel, J., Haq, I., Roberts, C., Garsia, R., Robertson, T., & Mellis, C. (2017). Team-based learning (TBL) in the medical curriculum: better than PBL?. *BMC medical education*, *17*, 1-11.	2017	80
Della Ratta, C. B. (2015). Flipping the classroom with team-based learning in undergraduate nursing education. *Nurse Educator*, *40*(2), 71-74.	2015	80
The positive impact of team-based virtual microscopy on student learning in physiology and histology	2007	67

Goldberg, H. R., & Dintzis, R. (2007). The positive impact of team-based virtual microscopy on student learning in physiology and histology. *Advances in physiology education*, *31*(3), 261-265.	2019	64
Frame, T. R., Cailor, S. M., Gryka, R. J., Chen, A. M., Kiersma, M. E., & Sheppard, L. (2015). Student perceptions of team-based learning vs traditional lecture-based learning. *American journal of pharmaceutical education*, *79*(4), 51.	2015	62
Poirier, T. I., Butler, L. M., Devraj, R., Gupchup, G. V., Santanello, C., & Lynch, J. C. (2009). A cultural competency course for pharmacy students. *American Journal of Pharmaceutical Education*, *73*(5).	2009	62
Abdelkhalek, N., Hussein, A., Gibbs, T., & Hamdy, H. (2010). Using team-based learning to prepare medical students for future problem-based learning. *Medical teacher*, *32*(2), 123-129.	2010	60
Thompson, B. M., Haidet, P., Borges, N. J., Carchedi, L. R., Roman, B. J., Townsend, M. H., ... & Levine, R. E. (2015). Team cohesiveness, team size and team performance in team-based learning teams. *Medical education*, *49*(4), 379-385.	2015	59
Tan, N. C., Kandiah, N., Chan, Y. H., Umapathi, T., Lee, S. H., & Tan, K. (2011). A controlled study of team-based learning for undergraduate clinical neurology education. *BMC medical education*, *11*, 1-8.	2011	58
Beatty, S. J., Kelley, K. A., Metzger, A. H., Bellebaum, K. L., & McAuley, J. W. (2009). Team-based learning in therapeutics workshop sessions. *American journal of pharmaceutical education*, *73*(6).	2009	56
Kim, H. R., Song, Y., Lindquist, R., & Kang, H. Y. (2016). Effects of team-based learning on problem-solving, knowledge and clinical performance of Korean nursing students. *Nurse education today*, *38*, 115-118.	2016	54
Doorenbos, A. Z., Gordon, D. B., Tauben, D., Palisoc, J., Drangsholt, M., Lindhorst, T., ... & Loeser, J. D. (2013). A blueprint of pain curriculum across prelicensure health sciences programs: one NIH Pain Consortium Center of Excellence in Pain Education (CoEPE) experience. *The journal of pain*, *14*(12), 1533-1538.	2013	53
Allen, R. E., Copeland, J., Franks, A. S., Karimi, R., McCollum, M., Riese II, D. J., & Lin, A. Y. (2013). Team-based learning in US colleges and schools of pharmacy. *American journal of pharmaceutical education*, *77*(6), 115.	2013	53
Thomas, P. A., & Bowen, C. W. (2011). A controlled trial of team-based learning in an ambulatory medicine clerkship for medical students. *Teaching and learning in medicine*, *23*(1), 31-36.	2011	53

Table 8 Studies with more than 50 citations

Co-Citations

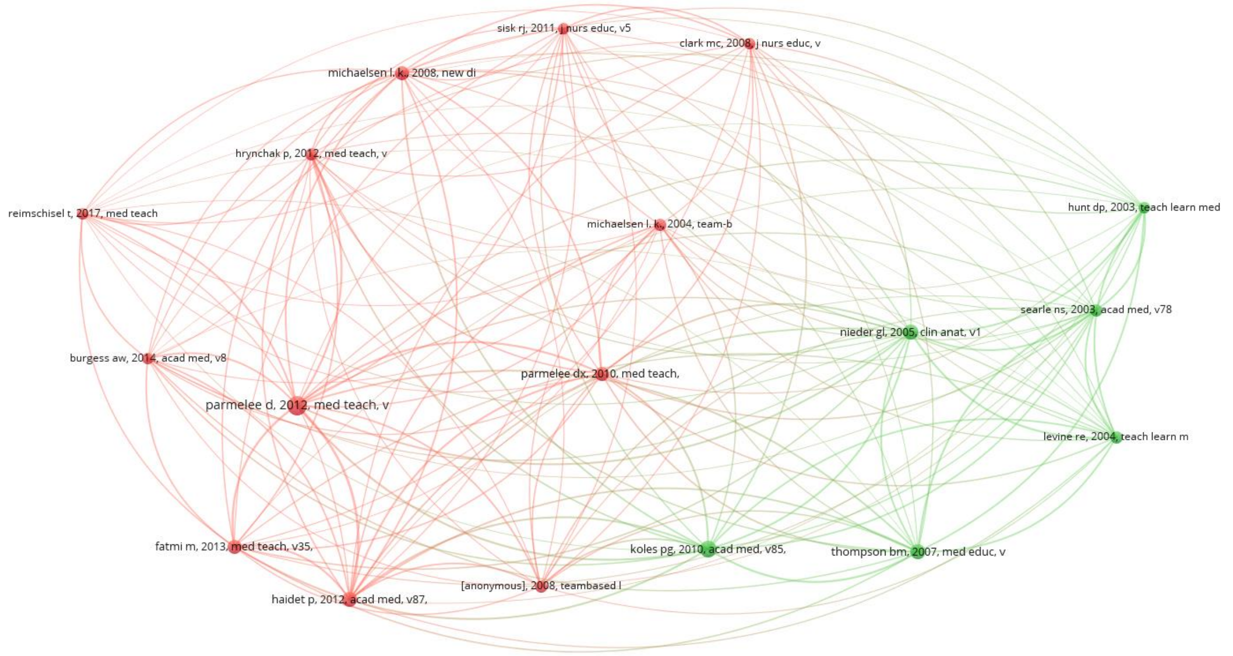

Figure 8 Co-citations.

Co-citation reference	Number of co-citations
Parmelee D, Michaelsen LK, Cook S, Hudes PD. Team-based learning: a practical guide: AMEE guide no. 65. Med Teach. 2012;34(5):e275-87. doi: 10.3109/0142159X.2012.651179. Epub 2012 Apr 4. PMID: 22471941.	146
Koles PG, Stolfi A, Borges NJ, Nelson S, Parmelee DX. The impact of team-based learning on medical students' academic performance. Acad Med. 2010 Nov;85(11):1739-45. doi: 10.1097/ACM.0b013e3181f52bed. PMID: 20881827.	107
Nieder GL, Parmelee DX, Stolfi A, Hudes PD. Team-based learning in a medical gross anatomy and embryology course. Clin Anat. 2005 Jan;18(1):56-63. doi: 10.1002/ca.20040. PMID: 15597377.	88
Haidet P, Levine RE, Parmelee DX, Crow S, Kennedy F, Kelly PA, Perkowski L, Michaelsen L, Richards BF. Perspective: Guidelines for reporting team-based learning activities in the medical and health sciences education literature. Acad Med. 2012 Mar;87(3):292-9. doi: 10.1097/ACM.0b013e318244759e. PMID: 22373620.	88
Thompson BM, Schneider VF, Haidet P, Levine RE, McMahon KK, Perkowski LC, Richards BF. Team-based learning at ten medical schools: two years later. Med Educ. 2007 Mar;41(3):250-7. doi: 10.1111/j.1365-2929.2006.02684.x. PMID: 17316209.	84
Parmelee DX, Michaelsen LK. Twelve tips for doing effective Team-Based Learning (TBL). Med Teach. 2010;32(2):118-22. doi: 10.3109/01421590903548562. PMID: 20163226.	82
Michaelsen, L. K., & Sweet, M. (2008). The essential elements of team-based learning. *New directions for teaching and learning*, *2008*(116), 7-27.	82
Fatmi, M., Hartling, L., Hillier, T., Campbell, S., & Oswald, A. E. (2013). The effectiveness of team-based learning on learning outcomes in health professions education: BEME Guide No. 30. *Medical teacher*, *35*(12), e1608-e1624.	78
Hrynchak, P., & Batty, H. (2012). The educational theory basis of team-based learning. *Medical teacher*, *34*(10), 796-801.	63
Michaelsen, L. K., Knight, A. B., & Fink, L. D. (Eds.). (2023). *Team-based learning: A transformative use of small groups in college teaching*. Taylor & Francis.	60
Searle, N. S., Haidet, P., Kelly, P. A., Schneider, V. F., Seidel, C. L., & Richards, B. F. (2003). Team learning in medical education: initial experiences at ten institutions. *Academic Medicine*, *78*(10), S55-S58.	58
Levine, R. E., O'Boyle, M., Haidet, P., Lynn, D. J., Stone, M. M., Wolf, D. V., & Paniagua, F. A. (2004). Transforming a clinical clerkship with team learning. *Teaching and learning in medicine*, *16*(3), 270-275.	57
Burgess, A. W., McGregor, D. M., & Mellis, C. M. (2014). Applying established guidelines to team-based learning programs in medical schools: a systematic review. *Academic medicine*, *89*(4), 678-688.	56
Clark, M. C., Nguyen, H. T., Bray, C., & Levine, R. E. (2008). Team-based learning in an undergraduate nursing course. *Journal of Nursing Education*, *47*(3), 111-117.	53
Hunt, D. P., Haidet, P., Coverdale, J. H., & Richards, B. (2003). The effect of using team learning in an evidence-based medicine course for medical students. *Teaching and learning in medicine*, *15*(2), 131-139.	51
Sisk, R. J. (2011). Team-based learning: systematic research review. *Journal of Nursing Education*, *50*(12), 665-669.	51
Reimschisel, T., Herring, A. L., Huang, J., & Minor, T. J. (2017). A systematic review of the published literature on team-based learning in health professions education. *Medical teacher*, *39*(12), 1227-1237.	51

Table 9 References cited more than 50 times.

Supplementary materials 3 – Evidence Summary

Author Full Names	Article Title	Source Title	Pub. Year	DOI	Affiliations	Country	Field	Solo pub.	Collab.: within the institution	Collab.: within the country	Collab.: international	10 initial instit.
Nieder, GL; Parmelee, DX; Stolfi, A; Hudes, PD	Team-based learning in a medical gross anatomy and embryology course	CLINICAL ANATOMY	2005	10.1002/ca.20040	Wright State University Dayton; Wright State University Dayton; Wright State University Dayton; Wright State University Dayton	USA	Medicine		one institution, many authors			yes
Touchet, BK; Coon, KA	A pilot use of team-based learning in psychiatry resident psychodynamic psychotherapy education	ACADEMIC PSYCHIATRY	2005	10.1176/appi.ap.29.3.293	University of Oklahoma - Tulsa; University of Oklahoma - Tulsa	USA	Medicine		one institution, many authors			yes
Koles, P; Nelson, S; Stolfi, A; Parmelee, D; DeStephen, D	Active learning in a Year 2 pathology curriculum	MEDICAL EDUCATION	2005	10.1111/j.1365-2929.2005.02248.x	Wright State University Dayton; Wright State University Dayton; Wright State University Dayton; Wright State University Dayton; Wright State University Dayton	USA	Medicine		one institution, many authors			yes
Ortega, Rafael A.; Stanley, Glynne; Snavely, Adam	Using a Media Centre to Facilitate Team-Based Learning	JOURNAL OF VISUAL COMMUNICATION IN MEDICINE	2006	10.1080/01405110600863357	Boston University; Boston University; Boston University	USA	Medicine		one institution, many authors			
Thompson, Britta M.; Schneider, Virginia F.; Haidet, Paul; Levine, Ruth E.; McMahon, Kathryn K.; Perkowski, Linda C.; Richards, Boyd F.	Team-based learning at ten medical schools: two years later	MEDICAL EDUCATION	2007	10.1111/j.1365-2929.2006.02684.x	Baylor College of Medicine; Houston Vet Affairs Med Ctr; University of Texas - Galveston; Texas Tech University Health Science Center; University of Minnesota Twin Cities	USA	Medicine			USA - multiple states		yes
Kramer, Paula; Ideishi, Roger I.; Kearney, Pamalyn J.; Cohen, Michelle E.; Ames, Joanne Oppermann; Shea, Geraldine Borelli; Schemm, Ruth; Blumberg, Phyllis	Achieving Curricular Themes Through Learner-Centered Teaching	OCCUPATIONAL THERAPY IN HEALTH CARE	2007	10.1300/J003v21n01_14	Univ Sci Philadelphia; Univ Sci Philadelphia; Univ Sci Philadelphia; Univ Sci Philadelphia; Univ Sci Philadelphia; Univ Sci Philadelphia; Univ Sci Philadelphia;	USA	Medicine		one institution, many authors			
Levine, Ruth E.; Kelly, P. Adam; Karakoc, Tayfun; Haidet, Paul	Peer evaluation in a clinical clerkship: Students' attitudes, experiences, and correlations with traditional assessments	ACADEMIC PSYCHIATRY	2007	10.1176/appi.ap.31.1.19	University of Texas Medical Branch Galveston; Baylor College of Medicine; University of Texas Medical Branch Galveston; Baylor College of Medicine	USA	Medicine			USA - one state		yes
Vasan, Nagaswami S.; DeFouw, David O.; Holland, Bart K.	Modified Use of Team-Based Learning for Effective Delivery of Medical Gross Anatomy and Embryology	ANATOMICAL SCIENCES EDUCATION	2008	10.1002/ase.5	Univ Med & Dent New Jersey; Univ Med & Dent New Jersey; Univ Med & Dent New Jersey	USA	Medicine		one institution, many authors			
Kuehne-Eversmann, Lisa; Eversmann, Thomas; Fischer, Martin R.	Team- and case-based learning to activate participants and enhance knowledge:: An evaluation of seminars in Germany	JOURNAL OF CONTINUING EDUCATION IN THE HEALTH PROFESSIONS	2008	10.1002/chp.175	University of Munich; University of Munich; University of Munich	Germany	Medicine		one institution, many authors			
Durosaro, Olayemi; Lachman, Nirusha; Pawlina, Wojciech	Use of Knowledge-sharing Web-based Portal in Gross and Microscopic Anatomy	ANNALS ACADEMY OF MEDICINE SINGAPORE	2008		Mayo Clinic; Mayo Clinic; Mayo Clinic	USA	Medicine		one institution, many authors			
Clark, Michele C.; Nguyen, Hoang Thanh; Bray, Chris; Levine, Ruth E.	Team-based learning in an undergraduate nursing course	JOURNAL OF NURSING EDUCATION	2008	10.3928/01484834-20080301-02	University of Nevada Las Vegas; University of Texas Medical Branch Galveston; University of Texas Medical Branch Galveston; University of Texas Medical Branch Galveston	USA	Nursing			USA - multiple states		yes
Kim, Soo Young	Students'Evaluation of a Team-based Course on Research and Publication Ethics: Attitude Change in Medical School Graduate Students	JOURNAL OF EDUCATIONAL EVALUATION FOR HEALTH PROFESSIONS	2008	10.3352/jeehp.2008.5.3	Hallym University	South Korea	Medicine	one author				
Beatty, Stuart J.; Kelley, Katherine A.; Metzger, Anne H.; Bellebaum, Katherine L.; McAuley, James W.	Team-based Learning in Therapeutics Workshop Sessions	AMERICAN JOURNAL OF PHARMACEUTICAL EDUCATION	2009	10.5688/aj7306100	Ohio State University; Ohio State University; Ohio State University; University of Cincinnati	USA	Pharmacy			USA - one state		
Chung, Eun-Kyung; Rhee, Jung-Ae; Baik, Young-Hong; A, Oh-Sun	The effect of team-based learning in medical ethics education	MEDICAL TEACHER	2009	10.3109/01421590802590553	Chonnam National University; Chonnam National University; Chonnam National University; Chonnam National University	South Korea	Medicine		one institution, many authors			
Wiener, Hubert; Plass, Herbert; Marz, Richard	Team-based Learning in Intensive Course Format for First-year Medical Students	CROATIAN MEDICAL JOURNAL	2009	10.3325/cmj.2009.50.69	Medical University of Vienna; Medical University of Vienna; Medical University of Vienna	Austria	Medicine		one institution, many authors			
Poirier, Therese I.; Butler, Lakesha M.; Devraj, Radhika; Gupchup, Gireesh V.; Santanello, Cathy; Lynch, J. Christopher	A Cultural Competency Course for Pharmacy Students	AMERICAN JOURNAL OF PHARMACEUTICAL EDUCATION	2009	10.5688/aj730581	Southern Illinois University Edwardsville; Southern Illinois University Edwardsville; Southern Illinois University Edwardsville; Southern Illinois University Edwardsville; Southern Illinois University Edwardsville; Southern Illinois University Edwardsville	USA	Pharmacy		one institution, many authors			

Gregory, Jeremy K.; Lachman, Nirusha; Camp, Christopher L.; Chen, Laura P.; Pawlina, Wojciech	Restructuring a basic science course for core competencies: An example from anatomy teaching	MEDICAL TEACHER	2009	10.1080/01421590903183795	Mayo Clinic; Mayo Clinic	USA	Medicine		one institution, many authors			
Shellenberger, Sylvia; Seale, J. Paul; Harris, Dona L.; Johnson, J. Aaron; Dodrill, Carrie L.; Velasquez, Mary M.	Applying Team-Based Learning in Primary Care Residency Programs to Increase Patient Alcohol Screenings and Brief Interventions	ACADEMIC MEDICINE	2009	10.1097/ACM.0b013e3181972855	Mercer University; Mercer University; Mercer University; Med Ctr Cent Georgia; Med Ctr Cent Georgia; University of Georgia; University of Texas Health Science Center Houston; University of Texas Austin	USA	Medicine			USA - multiple states		yes
Vasan, Nagaswami S.; DeFouw, David O.; Compton, Scott	A Survey of Student Perceptions of Team-Based Learning in Anatomy Curriculum: Favorable Views Unrelated to Grades	ANATOMICAL SCIENCES EDUCATION	2009	10.1002/ase.91	Univ Med & Dent New Jersey; Univ Med & Dent New Jersey; Univ Med & Dent New Jersey	USA	Medicine		one institution, many authors			
Koles, Paul G.; Stolfi, Adrienne; Borges, Nicole J.; Nelson, Stuart; Parmelee, Dean X.	The Impact of Team-Based Learning on Medical Students' Academic Performance	ACADEMIC MEDICINE	2010	10.1097/ACM.0b013e3181f52bed	Wright State University Dayton; Wright State University Dayton; Wright State University Dayton; Wright State University Dayton; Wright State University Dayton	USA	Medicine		one institution, many authors			yes
Abdelkhalek, Nahed; Hussein, Amal; Gibbs, Trevor; Hamdy, Hossam	Using team-based learning to prepare medical students for future problem-based learning	MEDICAL TEACHER	2010	10.3109/01421590903548539	University of Sharjah; Chinese University of Hong Kong	UAE+China	Medicine				China+UAE	
Ravindranath, Divy; Gay, Tamara L.; Riba, Michelle B.	Trainees as Teachers in Team-Based Learning	ACADEMIC PSYCHIATRY	2010	10.1176/appi.ap.34.4.294	University of Michigan; University of Michigan; University of Michigan	USA	Medicine		one institution, many authors			
Zgheib, Nathalie K.; Simaan, Joseph A.; Sabra, Ramzi	Using team-based learning to teach pharmacology to second year medical students improves student performance	MEDICAL TEACHER	2010	10.3109/01421590903548521	American University of Beirut; American University of Beirut; American University of Beirut	Lebanon	Medicine		one institution, many authors			
Parmelee, Dean X.; Michaelsen, Larry K.	Twelve tips for doing effective Team-Based Learning (TBL)	MEDICAL TEACHER	2010	10.3109/01421590903548562	Wright State University Dayton; University Central Missouri; University of Oklahoma - Norman	USA	Medicine			USA - multiple states		yes
Grady, Sarah E.	Team-based Learning in Pharmacotherapeutics	AMERICAN JOURNAL OF PHARMACEUTICAL EDUCATION	2011	10.5688/ajpe757136	Drake University; Broadlawns Med Ct	USA	Pharmacy	one author				
Zgheib, N. K.; Simaan, J. A.; Sabra, R.	Using Team-based Learning to Teach Clinical Pharmacology in Medical School: Student Satisfaction and Improved Performance	JOURNAL OF CLINICAL PHARMACOLOGY	2011	10.1177/0091270010375428	American University of Beirut; American University of Beirut; American University of Beirut	Lebanon	Pharmacy		one institution, many authors			
Hasan, Sanah	Teaching ethics to pharmacy students using a team-based learning approach	PHARMACY EDUCATION	2011		University of Sharjah	UAE	Pharmacy	one author				
Thomas, Patricia A.; Bowen, Craig W.	A Controlled Trial of Team-Based Learning in an Ambulatory Medicine Clerkship for Medical Students	TEACHING AND LEARNING IN MEDICINE	2011	10.1080/10401334.2011.536888	Johns Hopkins University; Johns Hopkins University	USA	Medicine		one institution, many authors			
Vasan, Nagaswami S.; DeFouw, David O.; Compton, Scott	Team-Based Learning in Anatomy: An Efficient, Effective, and Economical Strategy	ANATOMICAL SCIENCES EDUCATION	2011	10.1002/ase.257	Univ Med & Dent New Jersey; Univ Med & Dent New Jersey; Univ Med & Dent New Jersey	USA	Medicine			USA - one state		
Garrett, Bernard M.; MacPhee, Maura; Jackson, Cathryn	Implementing high-fidelity simulation in Canada: Reflections on 3 years of practice	NURSE EDUCATION TODAY	2011	10.1016/j.nedt.2010.10.028	University of British Columbia; University of British Columbia; University of British Columbia	Canada	Nursing		one institution, many authors			
Davidson, Lindsay K.	A 3-year experience implementing blended TBL: Active instructional methods can shift student attitudes to learning	MEDICAL TEACHER	2011	10.3109/0142159X.2011.558948	Queens University - Canada	Canada	Medicine	one author				
Gallegos, Patrick J.; Peeters, J. Michael	A measure of teamwork perceptions for team-based learning	CURRENTS IN PHARMACY TEACHING AND LEARNING	2011	10.1016/j.cptl.2010.10.004	Northeast Ohio Medical University (NEOMED); Akron General Medical Center; University of Toledo	USA	Pharmacy			USA - one state		
Tan, Nigel C. K.; Kandiah, Nagaendran; Chan, Yiong Huak; Umapathi, Thirugnanam; Lee, Sze Haur; Tan, Kevin	A controlled study of team-based learning for undergraduate clinical neurology education	BMC MEDICAL EDUCATION	2011	10.1186/1472-6920-11-91	National Neuroscience Institute (NNI); National Neuroscience Institute (NNI); National Neuroscience Institute (NNI); National Neuroscience Institute (NNI); National Neuroscience Institute (NNI); National University of Singapore; National University of Singapore; National University of Singapore; National University of Singapore; National University of Singapore; Natl Univ Hlth Syst	Singapore	Medicine			Singapore		
Akl, Imad Bou; Ghaddar, Fatima; Sabra, Ramzi; Parmelee, Dean; Simaan, Joseph A.; Kanafani, Zeina A.; Zgheib, Nathalie K.	Teaching Clinical Pharmacology Using Team-Based Learning: A Comparison Between Third- and Fourth-Year Medical Students	JOURNAL OF CLINICAL PHARMACOLOGY	2012	10.1177/0091270011428986	American University of Beirut; American University of Beirut; American University of Beirut; American University of Beirut; American University of Beirut; American University of Beirut; Wright State University Dayton	USA+Lebanon	Pharmacy				Lebanon+USA	yes
Persky, Adam M.	The Impact of Team-Based Learning on a Foundational Pharmacokinetics Course	AMERICAN JOURNAL OF PHARMACEUTICAL EDUCATION	2012	10.5688/ajpe76231	University of North Carolina Chapel Hill	USA	Pharmacy	one author				yes
McAndrew, Sarah; Jackman, Carina; Sisto, Paola Palma	Medical student-developed obesity education program uses modified team-	MEDICAL TEACHER	2012	10.3109/0142159X.2012.668238	Medical College of Wisconsin; Medical College of Wisconsin; Northwestern University	USA	Medicine			USA - one state		

	based learning to motivate adolescents											
Burgess, Annette W.; Ramsey-Stewart, George; May, James; Mellis, Craig	Team-based learning methods in teaching topographical anatomy by dissection	ANZ JOURNAL OF SURGERY	2012	10.1111/j.1445-2197.2012.06077.x	University of Sydney; University of Sydney; University of Sydney; University of Sydney	Australia	Medicine		one institution, many authors			
Kolluru, Srikanth; Roesch, Darren M.; de la Fuente, Ayesha Akhtar	A Multi-Instructor, Team-Based, Active-Learning Exercise to Integrate Basic and Clinical Sciences Content	AMERICAN JOURNAL OF PHARMACEUTICAL EDUCATION	2012	10.5688/ajpe76233	Texas A&M University College; Texas A&M University College; Texas A&M University College	USA	Pharmacy		one institution, many authors			
Borges, Nicole J.; Kirkham, Karen; Deardorff, Adam S.; Moore, Jeremy A.	Development of emotional intelligence in a team-based learning internal medicine clerkship	MEDICAL TEACHER	2012	10.3109/0142159X.2012.687121	Wright State University Dayton; Wright State University Dayton	USA	Medicine		one institution, many authors			yes
Okubo, Yumiko; Ishiguro, Naoko; Suganuma, Taiyo; Nishikawa, Toshio; Takubo, Toshio; Kojimahara, Noriko; Yago, Rie; Nunoda, Shinichi; Sugihara, Shigetaka; Yoshioka, Toshimasa	Team-Based Learning, a Learning Strategy for Clinical Reasoning, in Students with Problem-Based Learning Tutorial Experiences	TOHOKU JOURNAL OF EXPERIMENTAL MEDICINE	2012	10.1620/tjem.227.23	Tokyo Women's Medical University; Tokyo Women's Medical University; Tokyo Women's Medical University; Tokyo Women's Medical University; Tokyo Women's Medical University; Tokyo Women's Medical University; Tokyo Women's Medical University; Tokyo Women's Medical University; Tokyo Women's Medical University; Tokyo Women's Medical University	Japan	Medicine		one institution, many authors			
Mennenga, Heidi A.	Development and Psychometric Testing of the Team-Based Learning Student Assessment Instrument	NURSE EDUCATOR	2012	10.1097/NNE.0b013e31825a87cc	South Dakota State University	USA	Nursing	one author				
St Clair, Katherine; Chihara, Laura	Team-Based Learning in a Statistical Literacy Class	JOURNAL OF STATISTICS EDUCATION	2012	10.1080/10691898.2012.11889633	Carleton College; Carleton College	USA	Medicine		one institution, many authors			
Parmelee, Dean X.; Hudes, Patricia	Team-based learning: A relevant strategy in health professionals' education	MEDICAL TEACHER	2012	10.3109/0142159X.2012.643267	Wright State University Dayton; Wright State University Dayton	USA	Medicine		one institution, many authors			yes
Shah, Shilpa; Meisenberg, Gerhard	Opinions about Teaching Modalities: A Comparison between Faculty and Students	EDUCATION RESEARCH INTERNATIONAL	2012	10.1155/2012/604052	Ross Univ; Ross Univ	Dominica	Medicine		one institution, many authors			
Sigalet, Elaine; Donnon, Tyrone; Grant, Vincent	Undergraduate Students' Perceptions of and Attitudes Toward a Simulation-Based Interprofessional Curriculum The KidSIM ATTITUDES Questionnaire	SIMULATION IN HEALTHCARE-JOURNAL OF THE SOCIETY FOR SIMULATION IN HEALTHCARE	2012	10.1097/SIH.0b013e318264499e	University of Calgary; University of Calgary; University of Calgary	Canada	Interprofessional		one institution, many authors			
Scheffer, Christian; Tauschel, Diethard; Neumann, Melanie; Lutz, Gabriele; Cysarz, Dirk; Heusser, Peter; Edelhaeuser, Friedrich	Integrative medical education: Educational strategies and preliminary evaluation of the Integrated Curriculum for Anthroposophic Medicine (ICURAM)	PATIENT EDUCATION AND COUNSELING	2012	10.1016/j.pec.2012.04.006	Witten Herdecke University; Witten Herdecke University; Witten Herdecke University; Gemeinschaftskrankenhaus Herdecke; Gemeinschaftskrankenhaus Herdecke	Germany	Medicine			Germany		
Nieder, Gary L.; Borges, Nicole J.	An eight-year study of online lecture use in a medical gross anatomy and embryology course	ANATOMICAL SCIENCES EDUCATION	2012	10.1002/ase.1289	Wright State University Dayton; Wright State University Dayton;	USA	Medicine		one institution, many authors			yes
Karpa, Kelly	Development and implementation of an herbal and natural product elective in undergraduate medical education	BMC COMPLEMENTARY AND ALTERNATIVE MEDICINE	2012	10.1186/1472-6882-12-57	Pennsylvania State University	USA	Medicine	one author				
Allen, Rondall E.; Copeland, Jeffrey; Franks, Andrea S.; Karimi, Reza; McCollum, Marianne; Riese, David J., II; Lin, Anne Y. F.	Team-Based Learning in US Colleges and Schools of Pharmacy	AMERICAN JOURNAL OF PHARMACEUTICAL EDUCATION	2013	10.5688/ajpe776115	Xavier University of Louisiana; University Incarnate Word; University of Tennessee; Pacific University; Regis University; Auburn University; Notre Dame Maryland Univ	USA	Pharmacy			USA - multiple states		
Pogge, Elizabeth	A Team-Based Learning Course on Nutrition and Lifestyle Modification	AMERICAN JOURNAL OF PHARMACEUTICAL EDUCATION	2013	10.5688/ajpe775103	Midwestern University	USA	Pharmacy	one author				
Mody, Sheila K.; Kiley, Jessica; Gawron, Lori; Garcia, Patricia; Hammond, Cassing	Team-based learning: a novel approach to medical student education in family planning	CONTRACEPTION	2013	10.1016/j.contraception.2012.07.012	Northwestern University; Northwestern University; Northwestern University; Northwestern University; Northwestern University	USA	Medicine		one institution, many authors			
Wamsley, Maria A.; Julian, Katherine A.; O'Sullivan, Patricia; McCance-Katz, Elinore F.; Batki, Steven L.; Satre, Derek D.; Satterfield, Jason	Team-Based Learning Exercise Efficiently Teaches Brief Intervention Skills to Medicine Residents	SUBSTANCE ABUSE	2013	10.1080/08897077.2013.787958	University of California San Francisco; University of California San Francisco; University of California San Francisco; University of California San Francisco; University of California San Francisco; University of California San Francisco; University of California San Francisco; San Francisco VA Medical Center; Kaiser Permanente	USA	Medicine			USA - one state		

Considine, Julie; Payne, Roslyn; Williamson, Stacey; Currey, Judy	Expanding nurse initiated X-rays in emergency care using team-based learning and decision support	AUSTRALASIAN EMERGENCY NURSING JOURNAL	2013	10.1016/j.aenj.2012.11.001	Deakin University; Northern Hosp; Northern Hosp; Deakin University	Australia	Nursing			Australia		
Suno, Manabu; Yoshida, Toshiko; Koyama, Toshihiro; Zamami, Yoshito; Miyoshi, Tomoko; Mizushima, Takaaki; Tanimotoa, Mitsune	The Effectiveness of Team-based Learning (TBL) as a New Teaching Approach for Pharmaceutical Care Education	YAKUGAKU ZASSHI-JOURNAL OF THE PHARMACEUTICAL SOCIETY OF JAPAN	2013	10.1248/yakushi.12-00254	Okayama University; Okayama University; Okayama University; Okayama University; Okayama University; Okayama University; Grad Sch Med Dent & Pharmaceut Sci; Grad Sch Med Dent & Pharmaceut Sci	Japan	Pharmacy			Japan		
Hickman, Susan E.; Wocial, Lucia D.	Team-Based Learning and Ethics Education in Nursing	JOURNAL OF NURSING EDUCATION	2013	10.3928/01484834-20131121-01	Indiana University Indianapolis; Indiana University Indianapolis;	USA	Nursing		one institution, many authors			
Corbridge, Susan J.; Corbridge, Tom; Tiffen, Jennifer; Carlucci, Melissa	Implementing Team-Based Learning in a Nurse Practitioner Curriculum	NURSE EDUCATOR	2013	10.1097/NNE.0b013e3182a0e416	University of Illinois Chicago; University of Illinois Chicago; University of Illinois Chicago; Northwestern University	USA	Nursing			USA - one state		
Nelson, Michael; Allison, S. Dean; McCollum, Marianne; Luckey, Stephen W.; Clark, David R.; Paulsen, Susan M.; Malhotra, Jodie; Brunner, Lane J.	The Regis Model for pharmacy education: A highly integrated curriculum delivered by Team-Based Learning™ (TBL)	CURRENTS IN PHARMACY TEACHING AND LEARNING	2013	10.1016/j.cptl.2013.07.002	Regis University; Regis University; Regis University; Regis University; Regis University; Regis University; Regis University; Denver Health Medical Center; Calif Northstate Univ	USA	Pharmacy			USA - one state		
Lubeck, Paula; Tschetter, Lois; Mennenga, Heidi	Team-Based Learning: An Innovative Approach to Teaching Maternal-Newborn Nursing Care	JOURNAL OF NURSING EDUCATION	2013	10.3928/01484834-20130121-02	South Dakota State University; South Dakota State University; South Dakota State University	USA	Nursing		one institution, many authors			
McMullen, Isabel; Cartledge, Jonathan; Levine, Ruth; Iversen, Amy	Team-based learning for psychiatry residents: a mixed methods study	BMC MEDICAL EDUCATION	2013	10.1186/1472-6920-13-124	South London & Maudsley NHS Trust; UCL; University of Texas Medical Branch Galveston; Weston Educ Ctr	USA+UK	Medicine				UK+USA	yes
Warrier, Kavita S.; Schiller, Jocelyn H.; Frei, Nicole R.; Haftel, Hilary M.; Christner, Jennifer G.	Long-Term Gain After Team-Based Learning Experience in a Pediatric Clerkship	TEACHING AND LEARNING IN MEDICINE	2013	10.1080/10401334.2013.827975	University of Michigan System; University of Michigan; University of Michigan System; University of Michigan; University of Michigan System	USA	Medicine		one institution, many authors			
Gopalan, Chaya; Fox, Dainielle J.; Gaebelein, Claude J.	Effect of an individual readiness assurance test on a team readiness assurance test in the team-based learning of physiology	ADVANCES IN PHYSIOLOGY EDUCATION	2013	10.1152/advan.00095.2012	St Louis Coll Pharm; St Louis Coll Pharm; St Louis Coll Pharm	USA	Medicine		one institution, many authors			
Fujikura, Terumichi; Takeshita, Toshiyuki; Homma, Hiroshi; Adachi, Kouji; Miyake, Koichi; Kudo, Mitsuhiro; Takizawa, Takami; Nagayama, Hiroshi; Hirakawa, Keiko	Team-based Learning Using an Audience Response System: A Possible New Strategy for Interactive Medical Education	JOURNAL OF NIPPON MEDICAL SCHOOL	2013	10.1272/jnms.80.63	Nippon Medical School; Nippon Medical School; Nippon Medical School; Nippon Medical School; Nippon Medical School; Nippon Medical School; Nippon Medical School; Nippon Medical School; Nippon Medical School	Japan	Medicine		one institution, many authors			
Doucet, Shelley; Buchanan, Judy; Cole, Tricia; McCoy, Carolyn	A team approach to an undergraduate interprofessional communication course	JOURNAL OF INTERPROFESSIONAL CARE	2013	10.3109/13561820.2012.743978	University of New Brunswick; Dalhousie University; Dalhousie University; University of New Brunswick; New Brunswick Community Coll	Canada	Interprofessional		one institution, many authors			
Bow, Hansen C.; Dattilo, Jonathan R.; Jonas, Andrea M.; Lehmann, Christoph U.	A Crowdsourcing Model for Creating Preclinical Medical Education Study Tools	ACADEMIC MEDICINE	2013	10.1097/ACM.0b013e31828f86ef	Johns Hopkins University; Johns Hopkins University; Johns Hopkins University; Vanderbilt University	USA	Medicine			USA - multiple states		
Robinson, Michael A.; Robinson, Michelle Bachelor; McCaskill, Gina M.	Teaching Note-An Exploration of Team-Based Learning and Social Work Education: A Natural Fit	JOURNAL OF SOCIAL WORK EDUCATION	2013	10.1080/10437797.2013.812911	East Carolina University; East Carolina University; University of Alabama Tuscaloosa	USA	Medicine			USA - multiple states		
de Voest, Margaret; Raguckas, Sarah; Bambini, Deborah; Beel-Bates, Cindy	Interprofessional teaching: An inter-university experience involving pharmacy and nursing students	CURRENTS IN PHARMACY TEACHING AND LEARNING	2013	10.1016/j.cptl.2013.06.004	Ferris State University; Ferris State University; Grand Valley State University; Grand Valley State University	USA	Pharmacy			USA - one state		
Mennenga, Heidi A.	Student Engagement and Examination Performance in a Team-Based Learning Course	JOURNAL OF NURSING EDUCATION	2013	10.3928/01484834-20130718-04	South Dakota State University	USA	Nursing	one author				
Doorenbos, Ardith Z.; Gordon, Deborah B.; Tauben, David; Palisoc, Jenny; Drangsholt, Mark; Lindhorst, Taryn; Danielson, Jennifer; Spector, June; Ballweg, Ruth; Vorvick, Linda; Loeser, John D.	A Blueprint of Pain Curriculum Across Prelicensure Health Sciences Programs: One NIH Pain Consortium Center of Excellence in Pain Education (CoEPE) Experience	JOURNAL OF PAIN	2013	10.1016/j.jpain.2013.07.006	University of Washington Seattle; University of Washington Seattle; University of Washington Seattle; University of Washington Seattle; University of Washington Seattle; University of Washington Seattle; University of Washington Seattle; University of Washington Seattle; University of Washington Seattle; University of Washington Seattle; University of Washington Seattle	USA	Medicine		one institution, many authors			
Franks, Andrea S.	Enhancing Team-Based Active Learning Through Hands-On Experience With Nicotine Replacement Therapy	AMERICAN JOURNAL OF PHARMACEUTICAL EDUCATION	2013	10.5688/ajpe776128	University of Tennessee Health Science Center	USA	Pharmacy	one author				
Cox, Wendy C.; Kemp, Debra W.; Rodgers, Philip T.	Use of a team-based learning-influenced approach ambulatory care course	CURRENTS IN PHARMACY TEACHING AND LEARNING	2013	10.1016/j.cptl.2013.03.001	University of North Carolina Chapel Hill; University of North Carolina Chapel Hill; University of North Carolina Chapel Hill; Duke Area Hlth Educ Ctr	USA	Pharmacy			USA - multiple states		yes

Medina, Melissa S.; Conway, Susan E.; Davis-Maxwell, Tamra S.; Webb, Ryan	The Impact of Problem-Solving Feedback on Team-Based Learning Case Responses	AMERICAN JOURNAL OF PHARMACEUTICAL EDUCATION	2013	10.5688/ajpe779189	University of Oklahoma; University of Oklahoma; University of Oklahoma; University of Oklahoma;	USA	Pharmacy		one institution, many authors			yes
Clancy, Gerard P.; Duffy, F. Daniel	Going All In to Transform the Tulsa Community's Health and Health Care Workforce	ACADEMIC MEDICINE	2013	10.1097/ACM.0000000000000039	University of Oklahoma - Tulsa; University of Oklahoma - Tulsa	USA	Medicine		one institution, many authors			yes
Haj-Ali, Reem; Al Quran, Firas	Team-Based Learning in a Preclinical Removable Denture Prosthesis Module in a United Arab Emirates Dental School	JOURNAL OF DENTAL EDUCATION	2013		University of Sharjah; University of Sharjah; University of Missouri Kansas City; Jordan University of Science & Technology	USA+Jordan+UAE	dentistry				Jordan+UAE+USA	
Hagen, Brad; Awosoga, Olu; Kellett, Peter; Dei, Samuel Ofori	Evaluation of undergraduate nursing students' attitudes towards statistics courses, before and after a course in applied statistics	NURSE EDUCATION TODAY	2013	10.1016/j.nedt.2012.11.005	University of Lethbridge; University of Lethbridge; University of Lethbridge; University of Lethbridge	Canada	Nursing		one institution, many authors			
Roh, Young Sook; Lee, Suk Jeong; Mennenga, Heidi	Factors influencing learner satisfaction with team-based learning among nursing students	NURSING & HEALTH SCIENCES	2014	10.1111/nhs.12118	Chung Ang University; Chung Ang University; South Dakota State University	USA+South Korea	Nursing				South Korea+USA	
Johnson, June Felice; Bell, Edward; Bottenberg, Michelle; Eastman, Darla; Grady, Sarah; Koenigsfeld, Carrie; Maki, Erik; Meyer, Kristin; Phillips, Chuck; Schirmer, Lori	A Multiyear Analysis of Team-Based Learning in a Pharmacotherapeutics Course	AMERICAN JOURNAL OF PHARMACEUTICAL EDUCATION	2014	10.5688/ajpe787142	Drake University; Drake University; Drake University; Drake University; Drake University; Drake University; Drake University; Drake University; Drake University; Ft Sanders Reg Med Ctr	USA	Pharmacy			USA - multiple states		
Bleske, Barry E.; Remington, Tami L.; Wells, Trisha D.; Dorsch, Michael P.; Guthrie, Sally K.; Stumpf, Janice L.; Alaniz, Marissa C.; Ellingrod, Vicki L.; Tingen, Jeffrey M.	Team-Based Learning to Improve Learning Outcomes in a Therapeutics Course Sequence	AMERICAN JOURNAL OF PHARMACEUTICAL EDUCATION	2014	10.5688/ajpe78113	University of Michigan; University of Michigan; University of Michigan; University of Michigan; University of Michigan; University of Michigan; University of Michigan; University of Michigan; University of Michigan	USA	Pharmacy		one institution, many authors			
Altintas, Levent; Altintas, Ozgul; Caglar, Yusuf	Modified use of team-based learning in an ophthalmology course for fifth-year medical students	ADVANCES IN PHYSIOLOGY EDUCATION	2014	10.1152/advan.00129.2013	Kocaeli University; Kocaeli University; Kocaeli University	Turkey	Medicine		one institution, many authors			
Punja, Dhiren; Kalludi, Shivananda N.; Pai, Kirtana M.; Rao, Raghavendra K.; Dhar, Murali	Team-based learning as a teaching strategy for first-year medical students	AUSTRALASIAN MEDICAL JOURNAL	2014	10.4066/AMJ.2014.2244	Manipal Academy of Higher Education (MAHE); Manipal Academy of Higher Education (MAHE); Manipal Academy of Higher Education (MAHE); Akash Inst Med Sci & Res CtrKasturba Medical College, Manipal; Int Inst Populat Sci	India	Medicine			India		
Hashmi, Noreen Rahat	Team Based Learning (TBL) in Undergraduate Medical Education	JCPSP-JOURNAL OF THE COLLEGE OF PHYSICIANS AND SURGEONS PAKISTAN	2014		Rahbar Med & Dent Coll	Pakistan	Medicine	one author				
Gray, Jacob; Fana, Golden T.; Campbell, Thomas B.; Hakim, James G.; Borok, Margaret Z.; Aagaard, Eva M.	Feasibility and sustainability of an interactive team-based learning method for medical education during a severe faculty shortage in Zimbabwe	BMC MEDICAL EDUCATION	2014	10.1186/1472-6920-14-63	University of Colorado; University of Zimbabwe; University of Zimbabwe; University of Zimbabwe; University of Colorado; University of Zimbabwe; University of Zimbabwe; University of Zimbabwe; Alaska Native Medical Center	USA+Zimbabwe	Medicine				USA+Zimbabwe	
Livingston, Beven; Lundy, Mary; Harrington, Shana	Physical therapy students' perceptions of team-based learning in gross anatomy using the Team-Based Learning Student Assessment Instrument	JOURNAL OF EDUCATIONAL EVALUATION FOR HEALTH PROFESSIONS	2014	10.3352/jeehp.2014.11.1	University of North Florida; University of North Florida; University of North Florida; University of Florida	USA	Medicine			USA - one state		
Deardorff, Adam S.; Moore, Jeremy A.; McCormick, Colleen; Koles, Paul G.; Borges, Nicole J.	Incentive structure in team-based learning: graded versus ungraded Group Application exercises	JOURNAL OF EDUCATIONAL EVALUATION FOR HEALTH PROFESSIONS	2014	10.3352/jeehp.2014.11.6	Wright State University Dayton; Wright State University Dayton; Wright State University Dayton; Wright State University Dayton; Wright State University Dayton	USA	Medicine		one institution, many authors			yes
Elliott, Shannon	Using a Modified Team-Based Learning Approach to Teach Nursing Students About Communicable Disease Control and Community Health Nursing	JOURNAL OF NURSING EDUCATION	2014	10.3928/01484834-20141027-01	British Columbia Institute of Technology	Canada	Nursing	one author				
Hall, Jason; Freeman, Sally; Parmar, Harsha; Pluen, Alain	Team Based Learning: Preparing pharmacy students for an integrated curriculum during induction	PHARMACY EDUCATION	2014		Manchester Pharm Sch; Manchester Pharm Sch; Manchester Pharm Sch; Manchester Pharm Sch	UK	Pharmacy		one institution, many authors			
Cheng, Ching-Yu; Liou, Shwu-Ru; Hsu, Tsui-Hua; Pan, Mei-Yu; Liu, Hsiu-Chen; Chang, Chia-Hao	PREPARING NURSING STUDENTS TO BE COMPETENT FOR FUTURE PROFESSIONAL PRACTICE: APPLYING THE TEAM-BASED LEARNING	JOURNAL OF PROFESSIONAL NURSING	2014	10.1016/j.profnurs.2013.11.005	Chang Gong Univ Sci & Technol Chiayi Campus; Chang Gong Univ Sci & Technol Chiayi Campus; Chang Gong Univ Sci & Technol Chiayi Campus; Chang Gong Univ Sci & Technol Chiayi Campus	Taiwan	Nursing		one institution, many authors			

	TEACHING STRATEGY											
Nyindo, Mramba; Kitau, Jovin; Lisasi, Esther; Kapanda, Gibson; Matowo, Johnston; Francis, Patrick; Bartlett, John	Introduction of team-based learning (TBL) at Kilimanjaro Christian Medical University College: Experience with the ectoparasites module	MEDICAL TEACHER	2014	10.3109/0142159X.2013.876490	Kilimanjaro Christian Medical Centre; Kilimanjaro Christian Medical Centre; Kilimanjaro Christian Medical Centre; Kilimanjaro Christian Medical Centre; Kilimanjaro Christian Medical Centre; Duke University	USA+Tanzania	Medicine				Tanzania+USA	
Addo-Atuah, Joyce; Dutta, Arjun; Kovera, Craig	INSTRUCTIONAL DESIGN AND ASSESSMENT A Global Health Elective Course in a PharmD Curriculum	AMERICAN JOURNAL OF PHARMACEUTICAL EDUCATION	2014		Touro University; Marshall B Ketchum Uni; Janssen Pharmaceuticals	USA	Pharmacy			USA - multiple states		
Considine, Julie; Currey, Judy; Payne, Roslyn; Williamson, Stacey	Participant evaluation of team-based learning using one-off teams in a hospital setting	AUSTRALASIAN EMERGENCY NURSING JOURNAL	2014	10.1016/j.aenj.2014.03.002	Deakin University; Deakin University; Northern Hosp; Northern Hosp	Australia	Nursing			Australia		
Cheng, Ching-Yu; Liou, Shwu-Ru; Tsai, Hsiu-Min; Chang, Chia-Hao	The effects of Team-Based Learning on learning behaviors in the maternal-child nursing course	NURSE EDUCATION TODAY	2014	10.1016/j.nedt.2013.03.013	Chang Gung University of Science & Technology; Chang Gung University of Science & Technology; Chang Gung University of Science & Technology; Chang Gung University of Science & Technology	Taiwan	Nursing		one institution, many authors			
Elmore, Lindsey; Skelley, Jessica; Woolley, Thomas	Impact of adapted team-based learning methods on student self-assessment of professionalism, teamwork, and skills in a self-care course	CURRENTS IN PHARMACY TEACHING AND LEARNING	2014	10.1016/j.cptl.2014.04.002	St Vincents Hlth Sys; Samford University; Samford University	USA	Pharmacy		one institution, many authors			
Martinez, Emilio G.; Tuesca, Rafael	Modified Team-Based Learning Strategy to Improve Human Anatomy Learning: A Pilot Study at the Universidad del Norte in Barranquilla, Colombia	ANATOMICAL SCIENCES EDUCATION	2014	10.1002/ase.1444	Universidad del Norte Colombia; Universidad del Norte Colombia	Colombia	Medicine		one institution, many authors			
Ambizas, Emily M.; Bastianelli, Karen M. S.; Ferreri, Stefanie P.; Haines, Seena L.; Orr, Katherine Kelly; Stutz, Misty M.; VanAmburgh, Jenny A.; Wilhelm, Miranda	Evolution of Self-Care Education	AMERICAN JOURNAL OF PHARMACEUTICAL EDUCATION	2014		University of Rhode Island; Saint John's University; University of Minnesota Duluth; University of North Carolina Chapel Hill; Palm Beach Atlantic University West Palm Beach; Sullivan Univ; Northeastern University; Southern Illinois University System; Southern Illinois University Edwardsville	USA	Pharmacy			USA - multiple states		yes
Bandiera, Glen; LeBlanc, Constance; Regehr, Glenn; Snell, Linda; Frank, Jason R.; Sherbino, Jonathan	Education scholarship in emergency medicine part 2: supporting and developing scholars	CANADIAN JOURNAL OF EMERGENCY MEDICINE	2014		University of Toronto; Dalhousie University; University of British Columbia; McGill University; University of Ottawa; McMaster University	Canada	Medicine			Canada		
Brandler, Tamar C.; Laser, Jordan; Williamson, Alex K.; Louie, James; Esposito, Michael J.	Team-Based Learning in a Pathology Residency Training Program	AMERICAN JOURNAL OF CLINICAL PATHOLOGY	2014	10.1309/AJCPB8T1DZKCMWUT	Hofstra University; Hofstra University; Hofstra University; Hofstra University; Hofstra University	USA	Medicine		one institution, many authors			
Choi, Sandy Pin-pin; Cheung, Kin; Pang, Samantha Mei-che	A field study of the role of nurses in advocating for safe practice in hospitals	JOURNAL OF ADVANCED NURSING	2014	10.1111/jan.12316	Open Univ Hong Kong; Hong Kong Polytechnic University; Hong Kong Polytechnic University	China	Nursing			China		
Drazen, Jeffrey M.; Shields, Helen M.; Loscalzo, Joseph	A Division of Medical Communications in an Academic Medical Center's Department of Medicine	ACADEMIC MEDICINE	2014	10.1097/ACM.0000000000000472	Brigham & Women's Hospital; Brigham & Women's Hospital; Harvard University; Harvard University; Harvard University; Brigham & Women's Hospital	USA	Medicine			USA - one state		
Wright, Kelly J.; Frame, Tracy R.; Hartzler, Melody L.	Student perceptions of a Self-Care course taught exclusively by team-based learning and utilizing Twitter	CURRENTS IN PHARMACY TEACHING AND LEARNING	2014	10.1016/j.cptl.2014.07.003	Cedarville University; Cedarville University; Cedarville University	USA	Pharmacy		one institution, many authors			
Knox, Shane; Cullen, Walter; Dunne, Colum	Continuous Professional Competence (CPC) for Irish paramedics and advanced paramedics: a national study	BMC MEDICAL EDUCATION	2014	10.1186/1472-6920-14-41	University of Limerick; University of Limerick; University of Limerick	Ireland	Medicine		one institution, many authors			
Persky, Adam M.; Dupuis, Robert E.	An Eight-year Retrospective Study in Flipped Pharmacokinetics Courses	AMERICAN JOURNAL OF PHARMACEUTICAL EDUCATION	2014	10.5688/ajpe7810190	University of North Carolina Chapel Hill; University of North Carolina Chapel Hill	USA	Pharmacy		one institution, many authors			yes
Yang, Lian-Hong; Jiang, Long-Yuan; Xu, Bing; Liu, Shu-Qiong; Liang, Yan-Ran; Ye, Jin-Hao; Tao, En-Xiang	Evaluating team-based, lecture-based, and hybrid learning methods for neurology clerkship in China: a method-comparison study	BMC MEDICAL EDUCATION	2014	10.1186/1472-6920-14-98	Sun Yat Sen University; Sun Yat Sen University; Sun Yat Sen University; Sun Yat Sen University; Sun Yat Sen University; Sun Yat Sen University; Sun Yat Sen University	China	Medicine		one institution, many authors			
Fujikura, Terumichi; Nemoto, Takehiro; Takayanagi, Kazue; Kashimura, Masami; Hayasaka, Yoshiaki; Shimizu, Kazuo	A Freshman Orientation Program to Provide an Overview of the Medical Learning Roadmap	JOURNAL OF NIPPON MEDICAL SCHOOL	2014	10.1272/jnms.81.378	Nippon Medical School; Nippon Medical School; Nippon Medical School; Nippon Medical School; Nippon Medical School; Nippon Medical School	Japan	Medicine		one institution, many authors			

Moore-Davis, Tonia L.; Schorn, Mavis N.; Collins, Michelle R.; Phillippi, Julia; Holley, Sharon	Team-Based Learning for Midwifery Education	JOURNAL OF MIDWIFERY & WOMENS HEALTH	2015	10.1111/jmwh.12330	Vanderbilt University; Vanderbilt University; Vanderbilt University; Vanderbilt University; Vanderbilt University	USA	Midwifery		one institution, many authors			
Currey, Judy; Eustace, Paula; Oldland, Elizabeth; Glanville, David; Story, Ian	Developing professional attributes in critical care nurses using Team-Based Learning	NURSE EDUCATION IN PRACTICE	2015	10.1016/j.nepr.2015.01.011	Deakin University; Deakin University; Deakin University; Deakin University; Epworth Healthcare	Australia	Nursing			Australia		
Della Ratta, Carol B.	Flipping the Classroom With Team-Based Learning in Undergraduate Nursing Education	NURSE EDUCATOR	2015	10.1097/NNE.0000000000000112	State University of New York (SUNY) Stony Brook; Adelphi University	USA	Nursing	one author				
Currey, Judy; Oldland, Elizabeth; Considine, Julie; Glanville, David; Story, Ian	Evaluation of postgraduate critical care nursing students' attitudes to, and engagement with, Team-Based Learning: A descriptive study	INTENSIVE AND CRITICAL CARE NURSING	2015	10.1016/j.iccn.2014.09.003	Deakin University; Deakin University; Deakin University; Deakin University	Australia	Nursing			Australia		
Remington, Tami L.; Hershock, Chad; Klein, Kristin C.; Niemer, Rachel K.; Bleske, Barry E.	Lessons from the trenches: Implementing team-based learning across several courses	CURRENTS IN PHARMACY TEACHING AND LEARNING	2015	10.1016/j.cptl.2014.09.008	University of Michigan; University of Michigan; University of Michigan; Carnegie Mellon University; Mon Childrens Hosp; University of Michigan	USA	Pharmacy			USA - multiple states		
Park, Hyung-Ran; Kim, Chun-Ja; Park, Jee-Won; Park, Eunyoung	Effects of team-based learning on perceived teamwork and academic performance in a health assessment subject	COLLEGIAN	2015	10.1016/j.colegn.2014.05.001	Ajou University; Ajou University; Ajou University; Ajou University	South Korea	Medicine		one institution, many authors			
Ouellette, Patricia S.; Blount, Kamilah	Team-Based Learning in a Graduate Nurse Residency Program	JOURNAL OF CONTINUING EDUCATION IN NURSING	2015	10.3928/00220124-20151112-10	Vidant Med Ctr; Vidant Med Ctr	USA	Nursing		one institution, many authors			
Harmon, Rebecca Bouterie; Hills, Robin L.	Transforming Psychiatric Mental Health Nursing Education With Team Based Learning	ARCHIVES OF PSYCHIATRIC NURSING	2015	10.1016/j.apnu.2015.06.014	University of Virginia; University of Virginia	USA	Nursing		one institution, many authors			
Mennenga, Heidi A.	Time to Adjust Team-Based Learning 2 Years Later	NURSE EDUCATOR	2015	10.1097/NNE.0000000000000116	South Dakota State University	USA	Nursing	one author				
Whitley, Heather P.; Bell, Edward; Eng, Marty; Fuentes, David G.; Helms, Kristen L.; Maki, Erik D.; Vyas, Deepti	Practical Team-Based Learning from Planning to Implementation	AMERICAN JOURNAL OF PHARMACEUTICAL EDUCATION	2015	10.5688/ajpe7910149	Auburn University; Auburn University; Drake University; Drake University; Cedarville University; University of the Pacific; University of the Pacific;	USA	Pharmacy			USA - multiple states		
Bouw, Justin W.; Gupta, Vasudha; Hincapie, Ana L.	Assessment of students' satisfaction with a student-led team-based learning course	JOURNAL OF EDUCATIONAL EVALUATION FOR HEALTH PROFESSIONS	2015	10.3352/jeehp.2015.12.23	Calif Northstate Univ; Calif Northstate Univ; Calif Northstate Univ	USA	Medicine		one institution, many authors			
Frame, Tracy R.; Cailor, Stephanie M.; Gryka, Rebecca J.; Chen, Aleda M.; Kiersma, Mary E.; Sheppard, Lorin	Student Perceptions of Team-based Learning vs Traditional Lecture-based Learning	AMERICAN JOURNAL OF PHARMACEUTICAL EDUCATION	2015	10.5688/ajpe79451	Belmont University; Cedarville University; Cedarville University; Cedarville University; Univ Manchester; Univ Manchester	USA	Pharmacy			USA - multiple states		
Farland, Michelle Z.; Franks, Andrea S.; Barlow, Patrick B.; Rowe, A. Shaun; Chisholm-Burns, Marie	Assessment of student learning patterns, performance, and long-term knowledge retention following use of didactic lecture compared to team-based learning	CURRENTS IN PHARMACY TEACHING AND LEARNING	2015	10.1016/j.cptl.2014.12.009	University of Florida; University of Tennessee; University of Tennessee; University of Iowa; University of Tennessee	USA	Pharmacy			USA - multiple states		
Takeuchi, Hisahiro; Omoto, Katsuhiro; Okura, Kazuo; Tajima, Toyoko; Suzuki, Yoshitaka; Hosoki, Maki; Koori, Motoharu; Shigemoto, Shuji; Ueda, Mayu; Nishigawa, Keisuke; Rodis, Omar Marianito Maningo; Matsuka, Yoshizo	Effects of Team-Based Learning on Fixed Prosthodontic Education in a Japanese School of Dentistry	JOURNAL OF DENTAL EDUCATION	2015		Tokushima University; Tokushima University; Tokushima University; Tokushima University; Tokushima University; Tokushima University; Tokushima University; Tokushima University; Tokushima University; Tokushima University; Tokushima University; Tokushima University	Japan	dentistry		one institution, many authors			
Azzi, Alain J.; Ramnanan, Christopher J.; Smith, Jennifer; Dionne, Eric; Jalali, Alireza	To quiz or not to quiz: Formative tests help detect students at risk of failing the clinical anatomy course	ANATOMICAL SCIENCES EDUCATION	2015	10.1002/ase.1488	University of Ottawa; University of Ottawa; University of Ottawa; University of Ottawa; University of Ottawa	Canada	Medicine		one institution, many authors			
Huitt, Tiffany W.; Killins, Anita; Brooks, William S.	Team-Based Learning in the Gross Anatomy Laboratory Improves Academic Performance and Students' Attitudes Toward Teamwork	ANATOMICAL SCIENCES EDUCATION	2015	10.1002/ase.1460	University of Central Arkansas; University of Central Arkansas; Harding University; University of Alabama Birmingham	USA	Medicine			USA - multiple states		
Orr, Katherine Kelly; Feret, Brett M.; Lemay, Virginia A.; Cohen, Lisa B.; Mac Donnell, Celia P.; Seeram, Navindra; Hume, Anne L.	Assessment of a hybrid team-based learning (TBL) format in a required self-care course	CURRENTS IN PHARMACY TEACHING AND LEARNING	2015	10.1016/j.cpd.2015.04.016	University of Rhode Island; University of Rhode Island; University of Rhode Island; University of Rhode Island; University of Rhode Island; University of Rhode Island; University of Rhode Island	USA	Pharmacy		one institution, many authors			

Muzyk, Andrew J.; Fuller, Steve; Jiroutek, Michael R.; Grochowski, Colleen O'Connor; Butler, Andrew C.; May, D. Byron	Implementation of a flipped classroom model to teach psychopharmacotherapy to third-year Doctor of Pharmacy (PharmD) students	PHARMACY EDUCATION	2015		Campbell University; Campbell University; Campbell University; Campbell University; Duke University; Duke University	USA	Pharmacy			USA - one state		
Farland, Michelle Z.; Barlow, Patrick B.; Lancaster, T. Levi; Franks, Andrea S.	Comparison of Answer-Until-Correct and Full-Credit Assessments in a Team-based Learning Course	AMERICAN JOURNAL OF PHARMACEUTICAL EDUCATION	2015	10.5688/ajpe79221	University of Florida; University of Iowa, University of Tennessee - Knoxville; University of Tennessee - Knoxville	USA	Pharmacy			USA - multiple states		
Haber, Stacy L.; Boomershine, Virginia	An elective course in evidence-based health care using team-based learning	CURRENTS IN PHARMACY TEACHING AND LEARNING	2015	10.1016/j.cptl.2014.11.004	Midwestern University; Midwestern University	USA	Pharmacy		one institution, many authors			
Borges, Nicole J.; Thompson, Britta M.; Roman, Brenda J.; Townsend, Mark H.; Carchedi, Lisa R.; Cluver, Jeff S.; Frank, Julia B.; Haidet, Paul M.; Levine, Ruth E.	Team Emotional Intelligence, Team Interactions, and Gender in Medical Students During a Psychiatry Clerkship	ACADEMIC PSYCHIATRY	2015	10.1007/s40596-015-0282-4	Wright State University Dayton; Wright State University Dayton; University of Oklahoma; Louisiana State University; University of Texas Austin; Medical University of South Carolina; George Washington University; Pennsylvania State University; University of Texas Medical Branch Galveston	USA	Medicine			USA - multiple states		yes
Emke, Amanda R.; Cheng, Steven; Dufault, Carolyn; Cianciolo, Anna T.; Musick, David; Richards, Boyd; Violato, Claudio	Developing Professionalism via Multisource Feedback in Team-Based Learning	TEACHING AND LEARNING IN MEDICINE	2015	10.1080/10401334.2015.1077135	Washington University (WUSTL); Washington University (WUSTL); Washington University (WUSTL); Southern Illinois University; Virginia Polytechnic Institute & State University; Columbia University; Wake Forest University	USA	Medicine			USA - multiple states		
Sugihara, Takumichi	Current and Ideal Stages of Pharmaceutical Education	YAKUGAKU ZASSHI-JOURNAL OF THE PHARMACEUTICAL SOCIETY OF JAPAN	2015	10.1248/yakushi.14-00215-2	Niigata University	Japan	Pharmacy	one author				
Antoun, Jumana; Nasr, Rihab; Zgheib, Nathalie K.	Use of technology in the readiness assurance process of team based learning: Paper, automated response system, or computer based testing	COMPUTERS IN HUMAN BEHAVIOR	2015	10.1016/j.chb.2015.01.003	American University of Beirut; American University of Beirut; American University of Beirut	Lebanon	Medicine		one institution, many authors			
Catalino, Tricia; Chiarello, Lisa A.; Long, Toby; Weaver, Priscilla	Promoting Professional Development for Physical Therapists in Early Intervention	INFANTS & YOUNG CHILDREN	2015	10.1097/IYC.0000000000000034	Touro Univ Nevada; Drexel University; Georgetown University; Bradley University	USA	Medicine			USA - multiple states		
Dolmans, Diana; Michaelsen, Larry; Van Merrienboer, Jeroen; van der Vleuten, Cees	Should we choose between problem-based learning and team-based learning? No, combine the best of both worlds!	MEDICAL TEACHER	2015	10.3109/0142159X.2014.948828	Maastricht University; Maastricht University; Maastricht University; University Central Missouri	USA+Netherlands	Medicine				Netherlands+USA	
Persky, Adam M.; Henry, Teague; Campbell, Ashley	An Exploratory Analysis of Personality, Attitudes, and Study Skills on the Learning Curve within a Team-based Learning Environment	AMERICAN JOURNAL OF PHARMACEUTICAL EDUCATION	2015	10.5688/ajpe79220	University of North Carolina Chapel Hill; University of North Carolina Chapel Hill; University of North Carolina Chapel Hill	USA	Pharmacy		one institution, many authors			yes
Knollmann-Ritschel, Barbara E. C.; Durning, Steven J.	Using Concept Maps in a Modified Team-Based Learning Exercise	MILITARY MEDICINE	2015	10.7205/MILMED-D-14-00568	Uniformed Services University of the Health Sciences - USA; Uniformed Services University of the Health Sciences - USA	USA	Medicine		one institution, many authors			
Goodman, Barbara E.; Koster, Karen L.; Swanson, David L.	The development and implementation of a new medical biology major including physiology	ADVANCES IN PHYSIOLOGY EDUCATION	2015	10.1152/advan.00010.2015	University of South Dakota; University of South Dakota; University of South Dakota	USA	Medicine		one institution, many authors			
Carbrey, Jennifer M.; Grochowski, Colleen O'Connor; Cawley, Joseph; Engle, Deborah L.	A comparison of the effectiveness of the team-based learning readiness assessments completed at home to those completed in class	JOURNAL OF EDUCATIONAL EVALUATION FOR HEALTH PROFESSIONS	2015	10.3352/jeehp.2015.12.34	Duke University; Duke University; Duke University; Duke University	USA	Medicine		one institution, many authors			
Essa-Hadad, J.; Murdoch-Eaton, D.; Rudolf, M. C. J.	What impact does community service learning have on medical students' appreciation of population health?	PUBLIC HEALTH	2015	10.1016/j.puhe.2015.05.009	Bar Ilan Fac Med Galilee; Bar Ilan Fac Med Galilee; University of Sheffield	UK+Israel	Medicine				UK+Israel	
Zumberg, Marc S.; Broudy, Virginia C.; Bengtson, Elizabeth M.; Gitlin, Scott D.	Preclinical Medical Student Hematology/Oncology Education Environment	JOURNAL OF CANCER EDUCATION	2015	10.1007/s13187-014-0778-8	University of Florida; University of Washington; Dartmouth Hitchcock Med Ctr; University of Michigan	USA	Medicine			USA - multiple states		
Thompson, Britta M.; Haidet, Paul; Borges, Nicole J.; Carchedi, Lisa R.; Roman, Brenda J. B.; Townsend, Mark H.; Butler, Agata P.; Swanson, David B.; Anderson, Michael P.; Levine, Ruth E.	Team cohesiveness, team size and team performance in team-based learning teams	MEDICAL EDUCATION	2015	10.1111/medu.12636	University of Oklahoma Health Sciences Center; University of Oklahoma Health Sciences Center; Pennsylvania State University; Penn State Health; Wright State University Dayton; University of Texas Southwestern Medical Center Dallas; Wright State University Dayton; Louisiana State University Health Sciences Center New Orleans; University of Oklahoma Health Sciences Center; University of Texas Medical Branch Galveston	USA	Medicine			USA - multiple states		yes

Saudek, Kris; Treat, Robert	Team-based learning on a third-year pediatric clerkship improves NBME subject exam blood disorder scores	MEDICAL EDUCATION ONLINE	2015	10.3402/meo.v20.29021	Medical College of Wisconsin; Medical College of Wisconsin	USA	Medicine		one institution, many authors			
Anwar, Khurshid; Shaikh, Abdul A.; Sajid, Muhammad R.; Cahusac, Peter; Alarifi, Norah A.; Al Shedoukhy, Ahlam	Tackling student neurophobia in neurosciences block with team-based learning	MEDICAL EDUCATION ONLINE	2015	10.3402/meo.v20.28461	Alfaisal University; Alfaisal University; Alfaisal University; Alfaisal University; Alfaisal University; Alfaisal University	Saudi Arabia	Medicine		one institution, many authors			
Morris, Jenny	The use of team-based learning in a second year undergraduate pre-registration nursing course on evidence-informed decision making	NURSE EDUCATION IN PRACTICE	2016	10.1016/j.nepr.2016.09.005	University of Plymouth	UK	Nursing	one author				
Wilson, Jennifer A.; Waghel, Rashi C.; Free, Nicole R.; Borries, Alaina	Impact of team-based learning on perceived and actual retention of over-the-counter pharmacotherapy	CURRENTS IN PHARMACY TEACHING AND LEARNING	2016	10.1016/j.cptl.2016.06.008	Wingate University; Wingate University; Wingate University; Carolinas HealthCare System	USA	Pharmacy			USA - one state		
Franklin, Ann Snyder; Markowsky, Susan; De Leo, Justin; Normann, Sven; Black, Erik	Using Team-based Learning to Teach a Hybrid Pharmacokinetics Course Online and in Class	AMERICAN JOURNAL OF PHARMACEUTICAL EDUCATION	2016		University of Florida; University of Florida; University of Florida; University of Florida; University of Florida	USA	Pharmacy		one institution, many authors			
Hincapie, Ana L.; Cutler, Timothy W.; Fingado, Amanda R.	Incorporating Health Information Technology and Pharmacy Informatics in a Pharmacy Professional Didactic Curriculum-with a Team-based Learning Approach	AMERICAN JOURNAL OF PHARMACEUTICAL EDUCATION	2016		University of Cincinnati; University of California San Francisco; University of California San Francisco	USA	Pharmacy			USA - multiple states		
Kang, Kyung-Ah; Kim, Shin-Jeong; Oh, Jina; Kim, Sunghee; Lee, Myung-Nam	Effectiveness of simulation with team-based learning in newborn nursing care	NURSING & HEALTH SCIENCES	2016	10.1111/nhs.12245	Sahmyook University; Chung Ang University; Hallym University; Inje University; Kangwon National University	USA	Nursing			South Korea		
Ismail, Noor Akmal Shareela	Effectiveness of Team-Based Learning in teaching Medical Genetics to Medical Undergraduates	MALAYSIAN JOURNAL OF MEDICAL SCIENCES	2016		Universiti Kebangsaan Malaysia	Malaysia	Medicine	one author				
Burgess, Annette; Ayton, Tom; Mellis, Craig	Implementation of team-based learning in year 1 of a PBL based medical program: a pilot study	BMC MEDICAL EDUCATION	2016	10.1186/s12909-016-0550-3	University of Sydney; University of Sydney; University of Sydney	Australia	Medicine		one institution, many authors			
Obad, Adam S.; Peeran, Ahmed A.; Shareef, Mohammad Abrar; Alsheikh, Wissal J.; Kalagi, Dana A.; AlAmodi, Abdulhadi A.; Khan, Tehreem A.; Shaikh, Abdul Ahad; Ganguly, Paul; Yaqinuddin, Ahmed	Assessment of first-year medical students' perceptions of teaching and learning through team-based learning sessions	ADVANCES IN PHYSIOLOGY EDUCATION	2016	10.1152/advan.00001.2016	University of Mississippi; Alfaisal University; Alfaisal University; Alfaisal University, Alfaisal University; Alfaisal University; Alfaisal University; ; Alfaisal University; Alfaisal University	USA+Saudi Arabia	Medicine				Saudi Arabia+USA	
Pogge, Elizabeth	Evaluation of an interprofessional team-based learning nutrition and lifestyle modification course	JOURNAL OF INTERPROFESSIONAL CARE	2016	10.3109/13561820.2015.1092118	Midwestern University	USA	Interprofessional	one author				
Morris, Jenny	Implementation of a team-based learning course: Work required and perceptions of the teaching team	NURSE EDUCATION TODAY	2016	10.1016/j.nedt.2016.09.002	Knowledge Spa	UK	Nursing	one author				
Frame, Tracy R.; Gryka, Rebecca; Kiersma, Mary E.; Todt, Abby L.; Cailor, Stephanie M.; Chen, Aleda M. H.	Student Perceptions of and Confidence in Self-Care Course Concepts Using Team-based Learning	AMERICAN JOURNAL OF PHARMACEUTICAL EDUCATION	2016	10.5688/ajpe80346	Belmont University; Cedarville University; Cedarville University; Cedarville University; Accreditat Council Pharm Educ; Univ Manchester; Univ Manchester; Parkview Hlth	USA	Pharmacy			USA - multiple states		
Khodaveisi, Masoud; Qaderian, Khosro; Oshvandi, Khodayar; Soltanian, Ali Reza; Vardanjani, Mehdi Molavi; Khalili, Arash	Comparison of two methods: TBL-based and lecture-based learning in nursing care of patients with diabetes in nursing students	INTERNATIONAL JOURNAL OF MEDICAL RESEARCH & HEALTH SCIENCES	2016		Hamadan University of Medical Sciences; Hamadan University of Medical Sciences; Hamadan University of Medical Sciences; Hamadan University of Medical Sciences; Hamadan University of Medical Sciences; Hamadan University of Medical Sciences	Iran	Nursing		one institution, many authors			
Nation, Leanne Marie; Tweddell, Simon; Rutter, Paul	The applicability of a validated team-based learning student assessment instrument to assess United Kingdom pharmacy students' attitude toward team-based learning	JOURNAL OF EDUCATIONAL EVALUATION FOR HEALTH PROFESSIONS	2016	10.3352/jeehp.2016.13.30	University of Wolverhampton; University of Bradford; University of Central Lancashire	UK	Pharmacy			England		
Tweddell, Simon; Clark, David; Nelson, Michael	Team-based learning in pharmacy: The faculty experience	CURRENTS IN PHARMACY TEACHING AND LEARNING	2016	10.1016/j.cptl.2015.09.008	University of Bradford; Regis University; Regis University	USA+UK	Pharmacy				UK+USA	
Zgheib, Nathalie K.; Dimassi, Zakia; Akl, Imad Bou; Badr, Kamal F.; Sabra, Ramzi	The long-term impact of team-based learning on medical students' team performance scores and on their peer evaluation scores	MEDICAL TEACHER	2016	10.3109/0142159X.2016.1147537	American University of Beirut; American University of Beirut; American University of Beirut; American University of Beirut; American University of Beirut	Lebanon	Medicine		one institution, many authors			

Kim, Hae-Ran; Song, Yeoungsuk; Lindquist, Ruth; Kang, Hee-Young	Effects of team-based learning on problem-solving, knowledge and clinical performance of Korean nursing students	NURSE EDUCATION TODAY	2016	10.1016/j.nedt.2015.12.003	Honam University; Kyungpook National University (KNU); University of Minnesota Twin Cities; Chosun University	USA+South Korea	Nursing				South Korea+USA	
Behling, K. C.; Murphy, M. M.; Mitchell-Williams, J.; Rogers-McQuade, H.; Lopez, O. J.	Team-Based Learning in a Pipeline Course in Medical Microbiology for Under-Represented Student Populations in Medicine Improves Learning of Microbiology Concepts	JOURNAL OF MICROBIOLOGY & BIOLOGY EDUCATION	2016	10.1128/jmbe.v17i3.1083	Rowan University; Rowan University; Rowan University; Rowan University; Rowan University	USA	Medicine		one institution, many authors			
Boysen-Osborn, Megan; Anderson, Craig L.; Navarro, Roman; Yanuck, Justin; Strom, Suzanne; McCoy, Christopher E.; Youm, Julie; Ypma-Wong, Mary Frances; Langdorf, Mark I.	Flipping the Advanced Cardiac Life Support Classroom with Team-based Learning: Comparison of Cognitive Testing Performance for Medical Students at the University of California, Irvine, United States	JOURNAL OF EDUCATIONAL EVALUATION FOR HEALTH PROFESSIONS	2016	10.3352/jeehp.2016.13.11	University of California Irvine; University of California Irvine; University of California Irvine; University of California Irvine; University of California Irvine; University of California Irvine; University of California Irvine; University of California Irvine; University of California Irvine	USA	Medicine		one institution, many authors			
Fitzpatrick, Leo R.; Millette-Snodgrass, Carol; Atef, Eman	A Novel Mathematical Model for Determining Faculty Workload	AMERICAN JOURNAL OF PHARMACEUTICAL EDUCATION	2016		Calif Northstate Univ; Calif Northstate Univ; Calif Northstate Univ	USA	Pharmacy		one institution, many authors			
Feather, Rebecca A.; Carr, Doug E.; Reising, Deanna L.; Garletts, Derrick M.	Team-Based Learning for Nursing and Medical Students Focus Group Results From an Interprofessional Education Project	NURSE EDUCATOR	2016	10.1097/NNE.0000000000000240	Indiana University Bloomington; Indiana University Bloomington; Indiana University Bloomington; Indiana University Bloomington;	USA	Nursing		one institution, many authors			
Thrall, Grace C.; Coverdale, John H.; Benjamin, Sophiya; Wiggins, Anna; Lane, Christianne Joy; Pato, Michele T.	A Randomized Controlled Trial of Team-Based Learning Versus Lectures with Break-Out Groups on Knowledge Retention	ACADEMIC PSYCHIATRY	2016	10.1007/s40596-016-0501-7	Cent Reg Hosp; Baylor College of Medicine; McMaster University; University of Southern California; University of Southern California; University of Southern California; SUNY Downstate Health Sciences University	USA+Canada	Medicine				Canada+USA	yes
Holman, Michelle A.; Porter, Samuel G.; Pawlina, Wojciech; Juskewitch, Justin E.; Lachman, Nirusha	Does emotional intelligence change during medical school gross anatomy course? Correlations with students' performance and team cohesion	ANATOMICAL SCIENCES EDUCATION	2016	10.1002/ase.1541	Mayo Clinic; University of Kansas; Mayo Clinic; Mayo Clinic; Mayo Clinic	USA	Medicine			USA - multiple states		
Bleske, Barry E.; Remington, Tami L.; Wells, Trisha D.; Klein, Kristin C.; Guthrie, Sally K.; Tingen, Jeffrey M.; Marshall, Vincent D.; Dorsch, Michael P.	A Randomized Crossover Comparison of Team-based Learning and Lecture Format on Learning Outcomes	AMERICAN JOURNAL OF PHARMACEUTICAL EDUCATION	2016		University of Michigan; University of Michigan; University of Michigan; University of Michigan; University of Michigan; University of Michigan; University of Michigan; University of New Mexico; University of Virginia	USA	Pharmacy			USA - multiple states		
McKee, Anne	Developing and Assessing Teams Working Collaboratively Across Professions	ASSESSING COMPETENCE IN PROFESSIONAL PERFORMANCE ACROSS DISCIPLINES AND PROFESSIONS	2016	10.1007/978-3-319-30064-1_15	King's College London	UK	Medicine	one author				
Sherrier, William; Brennan, Teresa; Rabatsky, Ali	Chiropractic student attitudes toward team-based learning	JOURNAL OF CHIROPRACTIC EDUCATION	2016	10.7899/JCE-15-17	Palmer Coll Chiropract Florida; Palmer Coll Chiropract Florida; Palmer Coll Chiropract Florida	USA	Medicine		one institution, many authors			
Peacock, Justin G.; Grande, Joseph P.	An online app platform enhances collaborative medical student group learning and classroom management	MEDICAL TEACHER	2016	10.3109/0142159X.2015.1020290	San Antonio Military Medical Center; Mayo Clinic	USA	Medicine			USA - multiple states		
Huang, Zheqian; Li, Miaoling; Zhou, Yuxian; Ao, Yong; Xin, Wei; Jia, Yu; Yang, Ying; Cai, Yu; Xu, Chaochao; Yang, Yangfan; Lin, Haotian	Modified Team-Based Learning in an Ophthalmology Clerkship in China	PLOS ONE	2016	10.1371/journal.pone.0154250	Sun Yat Sen University; Sun Yat Sen University; Sun Yat Sen University; Sun Yat Sen University; Sun Yat Sen University; Sun Yat Sen University; Sun Yat Sen University; Sun Yat Sen University; Sun Yat Sen University; Sun Yat Sen University; Sun Yat Sen University	China	Medicine		one institution, many authors			
Wong, Arkers Kwan Ching; Wong, Frances Kam Yuet; Chan, Lap Ki; Chan, Namkiu; Ganotice, Fraide A.; Ho, Jacqueline	The effect of interprofessional team-based learning among nursing students: A quasi-experimental study	NURSE EDUCATION TODAY	2017	10.1016/j.nedt.2017.03.004	Hong Kong Polytechnic University; Hong Kong Polytechnic University; University of Hong Kong; Hong Kong Polytechnic University; University of Hong Kong; University of Hong Kong	China	Nursing			China		
Sharaf, Fawzy; Alnohair, Sultan	Comparison of medical students' learning approaches between electronic and hard copy team-based learning	INTERNATIONAL JOURNAL OF HEALTH SCIENCES-IJHS	2017		Qassim University; Qassim University	Saudi Arabia	Medicine		one institution, many authors			
Chen, Xianling; Chen, Buyuan; Li, Xiaofan; Song, Qingxiao; Chen, Yuanzhong	Mutual Benefit for Foreign Medical Students and Chinese Postgraduates: A Mixed Team-Based Learning Method Overcomes	BIOCHEMISTRY AND MOLECULAR BIOLOGY EDUCATION	2017	10.1002/bmb.20997	Fujian Med Univ	China	Medicine		one institution, many authors			

	Communication Problems in Hematology Clerkship											
Miller, Danielle M.; Khalil, Karen; Iskaros, Olivia; Van Amburgh, Jenny A.	Professional and pre-professional pharmacy students' perceptions of team based learning (TBL) at a private research-intensive university	CURRENTS IN PHARMACY TEACHING AND LEARNING	2017	10.1016/j.cptl.2017.03.001	Northeastern University; Northeastern University; Northeastern University; Lahey Hospital & Medical Center	USA	Pharmacy			USA - one state		
McRae, Marion E.; Chan, Alice; Lee, Ai Jin; Hulett, Renee; Coleman, Bernice	Team-Based Learning Improves Staff Nurses' Knowledge of Open- and Closed-Chest Cardiac Surgical Resuscitation	DIMENSIONS OF CRITICAL CARE NURSING	2017	10.1097/DCC.0000000000000221	Cedars Sinai Medical Center; Cedars Sinai Medical Center; University of California Los Angeles; University of California Los Angeles; Cedars Sinai Medical Center; St Catherine Hosp; Cedars Sinai Medical Center;	USA	Nursing			USA - one state		
Miles, Jane M.; Larson, Kim L.; Swanson, Melvin	Team-Based Learning in a Community Health Nursing Course: Improving Academic Outcomes	JOURNAL OF NURSING EDUCATION	2017	10.3928/01484834-20170619-07	Marquette University; East Carolina University; East Carolina University	USA	Nursing			USA - multiple states		
Oldland, Elizabeth; Currey, Judy; Considine, Julie; Allen, Josh	Nurses' perceptions of the impact of Team-Based Learning participation on learning style, team behaviours and clinical performance: An exploration of written reflections	NURSE EDUCATION IN PRACTICE	2017	10.1016/j.nepr.2017.03.008	Deakin University; Deakin University; Deakin University; Deakin University	Australia	Nursing		one institution, many authors			
Zeng, Rui; Xiang, Lian-rui; Zeng, Jing; Zuo, Chuan	Applying team-based learning of diagnostics for undergraduate students: assessing teaching effectiveness by a randomized controlled trial study	ADVANCES IN MEDICAL EDUCATION AND PRACTICE	2017	10.2147/AMEP.S127626	Sichuan University; Sichuan University; Sichuan University; Sichuan University	China	Medicine		one institution, many authors			
Remington, Tami L.; Bleske, Barry E.; Bartholomew, Tracy; Dorsch, Michael P.; Guthrie, Sally K.; Klein, Kristin C.; Tingen, Jeffrey M.; Wells, Trisha D.	Qualitative Analysis of Student Perceptions Comparing Team-based Learning and Traditional Lecture in a Pharmacotherapeutics Course	AMERICAN JOURNAL OF PHARMACEUTICAL EDUCATION	2017		University of Michigan; University of Michigan; University of Michigan; University of New Mexico; University of Michigan; University of Virginia	USA	Pharmacy			USA - multiple states		
Buhse, Marijean; Della Ratta, Carol	Enhancing Interprofessional Education With Team-Based Learning	NURSE EDUCATOR	2017	10.1097/NNE.0000000000000370	State University of New York (SUNY) Stony Brook; State University of New York (SUNY) Stony Brook	USA	Nursing		one institution, many authors			
Brich, Jochen; Jost, Meike; Bruestle, Peter; Giesler, Marianne; Rijntjes, Michel	Teaching neurology to medical students with a simplified version of team-based learning	NEUROLOGY	2017	10.1212/WNL.0000000000004211	University of Freiburg; University of Freiburg; University of Freiburg; Albert Ludwigs Univ Freiburg; Albert Ludwigs Univ Freiburg	Germany	Medicine		one institution, many authors			
Burgess, Annette; Bleasel, Jane; Haq, Inam; Roberts, Chris; Garsia, Roger; Robertson, Tomas; Mellis, Craig	Team-based learning (TBL) in the medical curriculum: better than PBL?	BMC MEDICAL EDUCATION	2017	10.1186/s12909-017-1068-z	University of Sydney; University of Sydney; University of Sydney; University of Sydney; University of Sydney; University of Sydney; University of Sydney; NSW Health; Royal Prince Alfred Hospital	Australia	Medicine			Australia		
Zinski, Anne; Blackwell, Kristina T. C. Panizzi Woodley; Belue, F. Mike; Brooks, William S.	Is lecture dead? A preliminary study of medical students' evaluation of teaching methods in the preclinical curriculum	INTERNATIONAL JOURNAL OF MEDICAL EDUCATION	2017	10.5116/ijme.59b9.5f40	University of Alabama Birmingham; University of Alabama Birmingham; University of Alabama Birmingham; University of Alabama Birmingham	USA	Medicine		one institution, many authors			
Kebodeaux, Clark D.; Peters, Golden L.; Stranges, Paul M.; Woodyard, Jamie L.; Vouri, Scott Martin	Faculty perception of team-based learning over multiple semesters	CURRENTS IN PHARMACY TEACHING AND LEARNING	2017	10.1016/j.cptl.2017.07.004	K Coll Pharm; St Louis Coll Pharm; University of Illinois Chicago; Purdue University System; Purdue University; St Louis Coll Pharm	USA	Pharmacy			USA - multiple states		
Lein, Donald H., Jr.; Lowman, John D.; Eidson, Christopher A.; Yuen, Hon K.	Evaluation of team-based learning in a doctor of physical therapy curriculum in the United States	JOURNAL OF EDUCATIONAL EVALUATION FOR HEALTH PROFESSIONS	2017	10.3352/jeehp.2017.14.3	University of Alabama Birmingham; University of Alabama Birmingham; University of Alabama Birmingham; University of Alabama Birmingham	USA	Medicine		one institution, many authors			
Emke, Amanda R.; Cheng, Steven; Chen, Ling; Tian, Dajun; Dufault, Carolyn	A Novel Approach to Assessing Professionalism in Preclinical Medical Students Using Multisource Feedback Through Paired Self- and Peer Evaluations	TEACHING AND LEARNING IN MEDICINE	2017	10.1080/10401334.2017.1306446	Washington University (WUSTL); Washington University (WUSTL); Washington University (WUSTL); Washington University (WUSTL); Washington University (WUSTL)	USA	Medicine		one institution, many authors			
Gryka, Rebecca; Kiersma, Mary E.; Frame, Tracy R.; Cailor, Stephanie M.; Chen, Aleda M. H.	Comparison of student confidence and perceptions of biochemistry concepts using a team-based learning versus traditional lecture-based format	CURRENTS IN PHARMACY TEACHING AND LEARNING	2017	10.1016/j.cptl.2016.11.020	Cedarville University; Cedarville University; Cedarville University; ACPE; Belmont University	USA	Pharmacy			USA - multiple states		
Behling, Kathryn C.; Kim, Rose; Gentile, Matthew; Lopez, Osvaldo	Does team-based learning improve performance in an infectious diseases course in a preclinical curriculum?	INTERNATIONAL JOURNAL OF MEDICAL EDUCATION	2017	10.5116/ijme.5895.0eea	Rowan University; Rowan University; Rowan University; Cooper University Hospital	USA	Medicine			USA - multiple states		
Fete, Matthew G.; Haight, Robert C.; Clapp, Peter; McCollum, Marianne	Peer Evaluation Instrument Development, Administration, and Assessment in a	AMERICAN JOURNAL OF PHARMACEUTICAL EDUCATION	2017		Regis University; Regis University; Regis University; Regis University	USA	Pharmacy		one institution, many authors			

	Team-based Learning Curriculum											
Eaton, Melody; deValpine, Maria; Sanford, Julie; Lee, Jamie; Trull, Laura; Smith, Kandy	Be the Change An Interprofessional Team-Based Health Advocacy Summit	NURSE EDUCATOR	2017	10.1097/NNE.0000000000000382	James Madison University; James Madison University; James Madison University; James Madison University; James Madison University; University of Mississippi	USA	Nursing			USA - multiple states		
Ono, Shin-ichi; Ito, Yoshihisa; Ishige, Kumiko; Inokuchi, Norio; Kosuge, Yasuhiro; Asami, Satoru; Izumisawa, Megumi; Kobayashi, Hiroko; Hayashi, Hiroyuki; Suzuki, Takashi; Kishikawa, Yukinaga; Hata, Harumi; Kose, Eiji; Tabata, Kei-ichi	Verification of Learning Effects by Team-based Learning	YAKUGAKU ZASSHI-JOURNAL OF THE PHARMACEUTICAL SOCIETY OF JAPAN	2017	10.1248/yakushi.17-00094	Nihon University; Nihon University; Nihon University; Nihon University; Nihon University; Nihon University; Nihon University; Nihon University; Nihon University; Nihon University; Nihon University; Nihon University; Nihon University; Nihon University	Japan	Pharmacy		one institution, many authors			
Sharma, Anita; Janke, Kristin K.; Larson, Andrea; Peter, Wendy St.	Understanding the early effects of team-based learning on student accountability and engagement using a three session TBL pilot	CURRENTS IN PHARMACY TEACHING AND LEARNING	2017	10.1016/j.cptl.2017.05.024	HealthEast Grand Ave Clin; University of Minnesota; Hlth East Midway Clin; University of Minnesota	USA	Pharmacy			USA - one state		
Asknes, Edna	Using Quantitative Literacy to Enhance Critical Thinking Skills in Undergraduate Nursing Students	JOURNAL OF NURSING EDUCATION	2017	10.3928/01484834-20170323-10	City University of New York (CUNY) System	USA	Nursing	one author				
Najdanovic-Visak, Vesna	Team-based learning for first year engineering students	EDUCATION FOR CHEMICAL ENGINEERS	2017	10.1016/j.ece.2016.09.001	Lancaster University	UK	Medicine	one author				
Lee, Shuh Shing	Has medical education killed silence?	MEDICAL TEACHER	2017	10.1080/0142159X.2016.1248919	National University of Singapore	Singapore	Medicine	one author				
Persky, Adam M.; Hogg, Abigail	Influence of Reading Material Characteristics on Study Time for Pre-Class Quizzes in a Flipped Classroom	AMERICAN JOURNAL OF PHARMACEUTICAL EDUCATION	2017		University of North Carolina Chapel Hill; University of North Carolina Chapel Hill	USA	Pharmacy			USA - multiple states		yes
Lein, Donald H., Jr.; Lowman, John D.; Eidson, Christopher A.; Yuen, Hon K.	Cross-validation of the Student Perceptions of Team-Based Learning Scale in the United States	JOURNAL OF EDUCATIONAL EVALUATION FOR HEALTH PROFESSIONS	2017	10.3352/jeehp.2017.14.15	University of Alabama Birmingham; University of Alabama Birmingham; University of Alabama Birmingham; University of Alabama Birmingham	USA	Medicine		one institution, many authors			
Jimborean, Gabriela; Ianosi, Edith Simona; Csipor, Alpar; Postolache, Paraschiva	Training the Future Trainers - Intensifying Anti-smoking Education for a Better Community Health	REVISTA DE CHIMIE	2017		Univ Med & Pharm Tg Mures; Univ Med & Pharm Tg Mures; Clin Cty Hosp Mures; Grigore T Popa Univ Med & Pharm	Romania	Medicine			Romania		
Dopheide, Julie A.; Bostwick, Jolene R.; Goldstone, Lisa W.; Thomas, Kelan; Nemire, Ruth; Gable, Kelly N.; Cates, Marshall; Caballero, Joshua; Smith, Tawny; Bainbridge, Jacquelyn	Curriculum in Psychiatry and Neurology for Pharmacy Programs	AMERICAN JOURNAL OF PHARMACEUTICAL EDUCATION	2017		University of Southern California; University of Southern California; University of Southern California; University of Michigan; Touro University California; Southern Illinois University Edwardsville; Samford University; Larkin Univ; University of Texas Austin; University of Colorado Anschutz Medical Campus; University of Colorado Anschutz Medical Campus	USA	Pharmacy			USA - multiple states		
Nishigawa, Keisuke; Omoto, Katsuhiro; Hayama, Rika; Okura, Kazuo; Tajima, Toyoko; Suzuki, Yoshitaka; Hosoki, Maki; Shigemoto, Shuji; Ueda, Mayu; Rodis, Omar Marianito Maningo; Matsuka, Yoshizo	Comparison between flipped classroom and team-based learning in fixed prosthodontic education	JOURNAL OF PROSTHODONTIC RESEARCH	2017	10.1016/j.jpor.2016.04.003	Tokushima University; Tokushima University; Tokushima University; Tokushima University; Tokushima University; Tokushima University; Tokushima University; Tokushima University; Tokushima University; Tokushima University; Tokushima Univ Hosp	Japan	Medicine			Japan		
Shiels, Lisa; Majmundar, Pratish; Zywot, Aleksander; Sobotka, John; Lau, Christine S. M.; Jalonen, Tuula O.	Medical student attitudes and educational interventions to prevent neurophobia: a longitudinal study	BMC MEDICAL EDUCATION	2017	10.1186/s12909-017-1055-4	St Georges Univ; St Georges Univ; St Georges Univ; St Georges Univ; St Georges Univ; St Georges Univ; Morristown Med Ctr	USA+Grenada	Medicine				Grenada+USA	
Alimoglu, Mustafa Kemal; Yardim, Selda; Uysal, Hilmi	The effectiveness of TBL with real patients in neurology education in terms of knowledge retention, in-class engagement, and learner reactions	ADVANCES IN PHYSIOLOGY EDUCATION	2017	10.1152/advan.00130.2016	Akdeniz University; Akdeniz University; Akdeniz University	Turkey	Medicine			Turkey		
Sherrill, Christina H.	Implementation and impact of a chronic kidney disease elective for second-year pharmacy students	CURRENTS IN PHARMACY TEACHING AND LEARNING	2017	10.1016/j.cptl.2016.11.005	Western New England University; High Point Univ	USA	Pharmacy	one author				

Alizadeh, Maryam; Mirzazadeh, Azim; Parmelee, Dean X.; Peyton, Elizabeth; Janani, Leila; Hassanzadeh, Gholamreza; Nedjat, Saharnaz	Uncover it, students would learn leadership from Team-Based Learning (TBL): The effect of guided reflection and feedback	MEDICAL TEACHER	2017	10.1080/0142159X.2017.1293237	Tehran University of Medical Sciences; Tehran University of Medical Sciences; Tehran University of Medical Sciences; Wright State University Dayton; Iran University of Medical Sciences; Tehran University of Medical Sciences; Tehran University of Medical Sciences; Tehran University of Medical Sciences	USA+Iran	Medicine				Iran+USA	yes
Branney, Jonathan; Priego-Hernandez, Jacqueline	A mixed methods evaluation of team-based learning for applied pathophysiology in undergraduate nursing education	NURSE EDUCATION TODAY	2018	10.1016/j.nedt.2017.11.014	Bournemouth University; University of Portsmouth	UK	Nursing			England		
Goktepe, Nilgun; Turkmen, Emine; Zeybekoglu, Zuhal; Yalcin, Begum	Use of Team-Based Learning in a Nursing Leadership Course An Action Research Study	NURSE EDUCATOR	2018	10.1097/NNE.0000000000000500	Koc University; Koc University; Koc University; Koc University	Turkey	Nursing		one institution, many authors			
DeJongh, Beth; Lemoine, Nicia; Buckley, Elizabeth; Traynor, Laura	Student preparation time for traditional lecture versus team-based learning in a pharmacotherapy course	CURRENTS IN PHARMACY TEACHING AND LEARNING	2018	10.1016/j.cptl.2017.11.009	Concordia University Wisconsin; Concordia University Wisconsin; Concordia University Wisconsin; Concordia University Wisconsin	USA	Pharmacy		one institution, many authors			
Kazory, Amir; Zaidi, Zareen	Team-Based Learning Activities for First-Year Medical Students: Perception of the Learners	SOUTHERN MEDICAL JOURNAL	2018	10.14423/SMJ.0000000000000865	University of Florida; University of Florida	USA	Medicine		one institution, many authors			
Lee, Kyung Eun	Effects of Team-Based Learning on the Core Competencies of Nursing Students: A Quasi-Experimental Study	JOURNAL OF NURSING RESEARCH	2018	10.1097/jnr.0000000000000259	Keimyung University	South Korea	Nursing	one author				
Eksteen, Mariet J.; Reitsma, Gerda M.; Swart, Sonet B.; Fourie, Erika	Team-Based Learning Experiences of Fourth-Year Pharmacy Students in a South African University	AMERICAN JOURNAL OF PHARMACEUTICAL EDUCATION	2018		University of the Free State; University of the Free State; North West University - South Africa; North West University - South Africa; North West University - South Africa	South Africa	Pharmacy			South Africa		
Falter, Rebecca A.; Ealey, Megan R.; Carroll, Kacey A.	Evaluation of modified team-based learning activities on student performance on therapeutic assessments	CURRENTS IN PHARMACY TEACHING AND LEARNING	2018	10.1016/j.cptl.2018.05.005	Shenandoah University; Shenandoah University; Shenandoah University; Butler University	USA	Pharmacy			USA - multiple states		
Park, Young-Sun; Kim, Jung-Ae; Jee, Young-Ju	Application of Team-Based Learning as a Teaching Method in Nursing Education	JOURNAL OF MEDICAL IMAGING AND HEALTH INFORMATICS	2018	10.1166/jmihi.2018.2327	Kyungnam University; Kyungbok Univ; Kyungbok Univ	South Korea	Nursing			South Korea		
Burgess, Annette; Roberts, Chris; Ayton, Tom; Mellis, Craig	Implementation of modified team-based learning within a problem based learning medical curriculum: a focus group study	BMC MEDICAL EDUCATION	2018	10.1186/s12909-018-1172-8	University of Sydney; University of Sydney; University of Sydney; University of Sydney	Australia	Medicine		one institution, many authors			
Quesnelle, Kelly M.; Bright, David R.; Salvati, Lisa A.	Interprofessional education through a telehealth team based learning exercise focused on pharmacogenomics	CURRENTS IN PHARMACY TEACHING AND LEARNING	2018	10.1016/j.cptl.2018.05.015	Western Michigan University; Ferris State University; Ferris State University	USA	Pharmacy			USA - one state		
Alwahab, Ahmed; Abdulqader, Saud; Nugud, Assmaa; Nugud, Shomous; Cyprian, Farhan; Shaikh, Abdul Ahad; Anwar, Khurshid	Team-based learning in an undergraduate pathology curriculum and its effects on student performance	JOURNAL OF TAIBAH UNIVERSITY MEDICAL SCIENCES	2018	10.1016/j.jtumed.2018.03.010	University of Sharjah; University of Sharjah; University of Sharjah; University of Sharjah; Ras AlKhaimah Med & Hlth Sci Univ; Alfaisal University; Alfaisal University	UAE+Saudi Arabia	Medicine				Saudi Arabia+UAE	
Fallon, Timothy; Strout, Tania D.	Free Open Access Medical Education (FOAM) Resources in a Team-Based Learning Educational Series	WESTERN JOURNAL OF EMERGENCY MEDICINE	2018	10.5811/westjem.2017.11.35091	Tufts Univ; Tufts Univ	USA	Medicine		one institution, many authors			
Tahira, Quratul Ain; Lodhi, Sidrah; Abaidullah, Sajid	Comparison of Lecture Based and Modified Team Based Learning in Achieving Cognitive Skills in Medical Education	ANNALS OF KING EDWARD MEDICAL UNIVERSITY LAHORE PAKISTAN	2018	10.21649/akemu.v24i1.2338	Mayo Hosp; Mayo Hosp; Mayo Hosp	Pakistan	Medicine		one institution, many authors			
Langdorf, Mark I.; Anderson, Craig L.; Navarro, Roman E.; Strom, Suzanne; Mccoy, C. Eric; Youm, Julie; Ipma-Wong, Mary Francis	Comparing the Results of Written Testing for Advanced Cardiac Life Support Teaching Using Team-based Learning and the Flipped Classroom Strategy	CUREUS JOURNAL OF MEDICAL SCIENCE	2018	10.7759/cureus.2574	University of California Irvine; University of California Irvine; University of California Irvine; University of California Irvine; University of California Irvine; University of California Irvine; University of California Irvine	USA	Medicine		one institution, many authors			
Salih, Karimeldin M. A.	Quality of Medical Students Performance through Team-Based Learning in Comparison to Performance in Traditional Lecture	PAKISTAN JOURNAL OF MEDICAL & HEALTH SCIENCES	2018		University of Bisha; University of Bahri	Saudi Arabia	Medicine	one author				

Jabbar, Hussein A.; Jarrahi, Abbas H.; Vamegh, Motahareh H.; Alhabahbeh, Dalia A. Moh'd; Mahmoud, Noor A.; Eladl, Mohamed A.	Effectiveness of the team-based learning (TBL) strategy on medical students' performance	JOURNAL OF TAIBAH UNIVERSITY MEDICAL SCIENCES	2018	10.1016/j.jtumed.2017.09.003	University of Sharjah; University of Sharjah; University of Sharjah; University of Sharjah; University of Sharjah; University of Sharjah; University of Sharjah; University of Sharjah	UAE	Medicine		one institution, many authors			
Chamberlin, Shaunta' M.; White, Cyle E.; Wheeler, James S.; Eudaley, Sarah T.; Franks, Andrea S.; Rowe, A. Shaun	Implementation and assessment of a pulmonary diseases elective course for third-year pharmacy students	CURRENTS IN PHARMACY TEACHING AND LEARNING	2018	10.1016/j.cptl.2018.02.007	University of Tennessee; University of Tennessee; University of Tennessee; University of Tennessee; University of Tennessee; University of Tennessee; Erlanger Hlth Syst	USA	Pharmacy			USA - one state		
Lexen, Annika; Hultqvist, Jenny; Amner, Gunilla	Occupational therapy student experiences of a university mental health course based on an integrated application of problem-based and team-based learning	SCANDINAVIAN JOURNAL OF OCCUPATIONAL THERAPY	2018	10.1080/11038128.2017.1367416	Lund University; Lund University; Lund University	Sweden	Occupational medicine		one institution, many authors			
Alizadeh, Maryam; Mirzazadeh, Azim; Parmelee, Dean X.; Peyton, Elizabeth; Mehrdad, Neda; Janani, Leila; Shahsavari, Hooman	Leadership Identity Development Through Reflection and Feedback in Team-Based Learning Medical Student Teams	TEACHING AND LEARNING IN MEDICINE	2018	10.1080/10401334.2017.1331134	Tehran University of Medical Sciences; Tehran University of Medical Sciences; Tehran University of Medical Sciences; Wright State University Dayton; Natl Ctr Org Dev; Tehran University of Medical Sciences; Iran University of Medical Sciences; Iran University of Medical Sciences; Tehran University of Medical Sciences; Tehran University of Medical Sciences	USA+Iran	Medicine				Iran+USA	yes
Luetmer, Marianne T.; Cloud, Beth A.; Youdas, James W.; Pawlina, Wojciech; Lachman, Nirusha	Simulating the multi-disciplinary care team approach: Enhancing student understanding of anatomy through an ultrasound-anchored interprofessional session	ANATOMICAL SCIENCES EDUCATION	2018	10.1002/ase.1731	Mayo Clinic; Mayo Clinic; Mayo Clinic; Mayo Clinic; Mayo Clinic	USA	Medicine		one institution, many authors			
Lochner, Lukas; Girardi, Sandra; Pavcovich, Alessandra; Meier, Horand; Mantovan, Franco; Ausserhofer, Dietmar	Applying interprofessional Team-Based Learning in patient safety: a pilot evaluation study	BMC MEDICAL EDUCATION	2018	10.1186/s12909-018-1164-8	Claudiana Coll Healthcare Profess; Claudiana Coll Healthcare Profess; Claudiana Coll Healthcare Profess; South Tyrolean Hlth Trust; South Tyrolean Hlth Trust; South Tyrolean Hlth Trust; South Tyrolean Hlth Trust; Clin Governance; University of Basel	Italy+Switzerland	Interprofessional				Italy+Switzerland	
Krase, Kelli; Pfeifer, Emily; Swan, Kimberly	Team-Based Learning Sessions Compared With Traditional Lecture in the Obstetrics and Gynecology Clerkship	OBSTETRICS AND GYNECOLOGY	2018	10.1097/AOG.0000000000002856	University of Kansas; University of Kansas; University of Kansas	USA	Medicine		one institution, many authors			
Eckardt, Patricia; Janotha, Brenda; Marino, Marie Ann; Erlanger, David P.; Cannella, Dolores	Equipping Advanced Practice Nurses With Real-World Skills	HEALTH CARE DELIVERY AND CLINICAL SCIENCE: CONCEPTS, METHODOLOGIES, TOOLS, AND APPLICATIONS	2018	10.4018/978-1-5225-3926-1.ch072	State University of New York (SUNY) Stony Brook; State University of New York (SUNY) Stony Brook; State University of New York (SUNY) Stony Brook; State University of New York (SUNY) Stony Brook	USA	Medicine		one institution, many authors			
Brooks, Marta J.; Nelson, Michael H.	A preliminary model for faculty workload for a highly integrated curriculum delivered by team-based learning	CURRENTS IN PHARMACY TEACHING AND LEARNING	2018	10.1016/j.cptl.2018.07.012	Regis University; Drake University	USA	Pharmacy			USA - multiple states		
da Rocha Cunha, Mariana Lucas; Amendola, Fernanda; Fernandez Samperiz, Maria Mercedes; da Costa Mohallem, Andrea Gomes	Evaluation of student perception of the Team-based Learning method (APA-TBL): Instrument construction and validation	NURSE EDUCATION IN PRACTICE	2018	10.1016/j.nepr.2018.09.008	FICSAE; FICSAE; FICSAE; FICSAE	Brazil	Nursing		one institution, many authors			
Farland, Michelle Z.; Feng Xiaoying; Franks, Andrea S.; Sando, Karen R.; Behar-Horenstein, Linda S.	Pharmacy resident teaching and learning curriculum program outcomes: Student performance and quality assessment	CURRENTS IN PHARMACY TEACHING AND LEARNING	2018	10.1016/j.cptl.2018.03.015	University of Florida; University of Florida; University of Tennessee - Knoxville; Nova Southeastern University; University of Florida; University of Florida; University of Florida	USA	Pharmacy			USA - one state		
Rotgans, Jerome I.; Schmidt, Henk G.; Rajalingam, Preman; Hao, Joey Wong Ying; Canning, Claire Ann; Ferenczi, Michael A.; Low-Beer, Naomi	How cognitive engagement fluctuates during a team-based learning session and how it predicts academic achievement	ADVANCES IN HEALTH SCIENCES EDUCATION	2018	10.1007/s10459-017-9801-2	Nanyang Technological University; Nanyang Technological University; Nanyang Technological University; Nanyang Technological University; Nanyang Technological University; Nanyang Technological University; Erasmus University Rotterdam	Netherlands+Singapore	Medicine				Netherlands+Singapore	
Rajati, Fatemeh; Sharifirad, Gholamreza; Babakhani, Maryam; Mohebi, Siamak	The effect of team-based learning on public health students' educational outcomes	JOURNAL OF EDUCATION AND HEALTH PROMOTION	2018	10.4103/jehp.jehp_124_17	Kermanshah University of Medical Sciences; Kermanshah University of Medical Sciences; Qom Univ Med Sci; Qom Univ Med Sci	Iran	Medicine			Iran		
Ganotice, Fraide A.; Chan, Lap Ki	Construct validation of the English version of Readiness for Interprofessional Learning Scale (RIPLS): Are Chinese undergraduate students ready for 'shared learning'?	JOURNAL OF INTERPROFESSIONAL CARE	2018	10.1080/13561820.2017.1359508	University of Hong Kong; University of Hong Kong	China	Interprofessional		one institution, many authors			

Bleske, Barry E.; Remington, Tami L.; Wells, Trisha D.; Klein, Kristin C.; Tingen, Jeffrey M.; Dorsch, Michael P.	A Randomized Crossover Comparison between Team-Based Learning and Lecture Format on Long-Term Learning Outcomes	PHARMACY	2018	10.3390/pharmacy6030081	University of New Mexico; University of Michigan; University of Michigan; University of Michigan; University of Virginia; University of Michigan;	USA	Pharmacy			USA - multiple states		
Rajalingam, Preman; Rotgans, Jerome I.; Zary, Nabil; Ferenczi, Michael Alan; Gagnon, Paul; Low-Beer, Naomi	Implementation of team-based learning on a large scale: Three factors to keep in mind	MEDICAL TEACHER	2018	10.1080/0142159X.2018.1451630	Nanyang Technological University; Nanyang Technological University; Nanyang Technological University; Nanyang Technological University; Nanyang Technological University; Nanyang Technological University	Singapore	Medicine		one institution, many authors			
Carrasco, Gonzalo A.; Behling, Kathryn C.; Lopez, Osvaldo J.	Evaluation of the role of incentive structure on student participation and performance in active learning strategies: A comparison of case-based and team-based learning	MEDICAL TEACHER	2018	10.1080/0142159X.2017.1408899	Rowan University; Rowan University; Seton Hall Hackensack Meridian Sch Med	USA	Medicine			USA - one state		
Riklefs, Viktor; Abakassova, Gulmira; Bukeyeva, Aliya; Kaliyeva, Sholpan; Serik, Bakhtiyar; Muratova, Alma; Dosmagambetova, Raushan	Transforming medical education in Kazakhstan: Successful case of internationalization from Karaganda State Medical University	MEDICAL TEACHER	2018	10.1080/0142159X.2018.1441989	Karaganda Medical University; Karaganda Medical University; Karaganda Medical University; Karaganda Medical University; Karaganda Medical University; Karaganda Medical University; Karaganda Medical University	Kazakhstan	Medicine		one institution, many authors			
Viswesh, Velliyur; Yang, Haoshu; Gupta, Vasudha	EVALUATION OF A MODIFIED DEBATE EXERCISE ADAPTED TO THE PEDAGOGY OF TEAM-BASED LEARNING	AMERICAN JOURNAL OF PHARMACEUTICAL EDUCATION	2018	10.5688/ajpe6278	Roseman Univ Hlth Sci; Roseman Univ Hlth Sci; Cedars Sinai Medical Center	USA	Pharmacy			USA - one state		
Li, Qing; Chen, Jianhui; Wan, Bo; Li, Xiaofan	No Gender Difference in Foreign Medical Students' Hematology Clerkship	EDUCATION RESEARCH INTERNATIONAL	2018	10.1155/2018/9069574	Fujian Medical University; Fujian Medical University; Fujian Medical University; Fujian Inst Hematol	China	Medicine			China		
Coyne, Leanne; Takemoto, Jody K.; Parmentier, Brittany L.; Merritt, Thayer; Sharpton, Rachel A.	Exploring virtual reality as a platform for distance team-based learning	CURRENTS IN PHARMACY TEACHING AND LEARNING	2018	10.1016/j.cptl.2018.07.005	University of Texas at Tyler; University of Texas at Tyler; University of Texas at Tyler; University of Texas at Tyler; University of Texas at Tyler	USA	Pharmacy		one institution, many authors			
Almasi, Mustapha; Zhu, Chang	Students' Perceptions of Social Presence in Blended Learning Courses in a Tanzanian Medical College	INTERNATIONAL JOURNAL OF EMERGING TECHNOLOGIES IN LEARNING	2018	10.3991/ijet.v13i09.8566	Vrije Universiteit Brussel; Vrije Universiteit Brussel	Belgium	Medicine		one institution, many authors			
Gradl-Dietsch, G.; Menon, A. K.; Guersel, A.; Goetzenich, A.; Hatam, N.; Aljalloud, A.; Schrading, S.; Hoelzl, F.; Knobe, M.	Basic echocardiography for undergraduate students: a comparison of different peer-teaching approaches	EUROPEAN JOURNAL OF TRAUMA AND EMERGENCY SURGERY	2018	10.1007/s00068-017-0819-1	RWTH Aachen University; RWTH Aachen University; University of Duisburg Essen; RWTH Aachen University; RWTH Aachen University; RWTH Aachen University; RWTH Aachen University; RWTH Aachen University; Med Ctr Marienhohe	Germany	Medicine			Germany		
Goolsarran, Nirvani; Hamo, Carine E.; Lane, Susan; Frawley, Stacey; Lu, Wei-Hsin	Effectiveness of an interprofessional patient safety team-based learning simulation experience on healthcare professional trainees	BMC MEDICAL EDUCATION	2018	10.1186/s12909-018-1301-4	State University of New York (SUNY) System; State University of New York (SUNY) Stony Brook; Stony Brook University Hospital; State University of New York (SUNY) System; State University of New York (SUNY) Stony Brook; Stony Brook University Hospital; State University of New York (SUNY) System; State University of New York (SUNY) Stony Brook; Stony Brook University Hospital; State University of New York (SUNY) System; State University of New York (SUNY) Stony Brook	USA	Interprofessional			USA - one state		
Fereshteh, Farhadi; Hadi, Mostafaie; Negar, Taleschian-Tabrizi; Sakineh, Hajebrahimi; Neda, Madani; Mohammadali, Hajebrahimi; Neda, Parnianfard; Morteza, Abbaspour; Amin, Talebpour; Fariba, Pashazadeh	Evidence based medicine summer school for undergraduate medical students using innovative methods	MEDICAL SCIENCE	2018		Tabriz University of Medical Science; Tabriz University of Medical Science; Tabriz University of Medical Science; Tabriz University of Medical Science; Tabriz University of Medical Science; Tabriz University of Medical Science; Tabriz University of Medical Science; Tabriz University of Medical Science; Tabriz University of Medical Science; Tabriz University of Medical Science	Iran	Medicine		one institution, many authors			
Tully, Vicki; Murphy, Douglas; Fioratou, Evridiki; Chaudhuri, Arun; Shaw, James; Davey, Peter	Learning from errors: assessing final year medical students' reflection on safety improvement, five year cohort study	BMC MEDICAL EDUCATION	2018	10.1186/s12909-018-1173-7	University of Dundee; University of Dundee; University of Dundee; University of Dundee; NHS Tayside; NHS Tayside; NHS Tayside	UK	Medicine			Scotland		
Alavi-Arjas, Fatemeh; Farnam, Farnaz; Granmayeh, Mehrnaz; Haghani, Hamid	The Effect of Sexual and Reproductive Health Education on Knowledge and Self-Efficacy of School Counselors	JOURNAL OF ADOLESCENT HEALTH	2018	10.1016/j.jadohealth.2018.05.031	Tehran University of Medical Sciences; Tehran University of Medical Sciences; Tehran University of Medical Sciences; Iran University of Medical Sciences; Islamic Azad University	Iran	Medicine			Iran		

Kek, Boon; Buchanan, Judith; Adisesh, Anil	An Introduction to Occupational Medicine Using a Team-Based Learning Methodology	JOURNAL OF OCCUPATIONAL AND ENVIRONMENTAL MEDICINE	2019	10.1097/JOM.0000000000001499	Dalhousie University; JD Irving Ltd; JD Irving Ltd	Canada	Occupational medicine			Canada		
Ozgonul, Levent; Alimoglu, Mustafa Kemal	Comparison of lecture and team-based learning in medical ethics education	NURSING ETHICS	2019	10.1177/0969733017731916	Akdeniz University; Akdeniz University	Turkey	Nursing		one institution, many authors			
Minges, Karl E.	Team-Based Learning in the Clinical Setting: Perspectives of Doctor of Nursing Practice Students	JOURNAL OF DOCTORAL NURSING PRACTICE	2019	10.1891/2380-9418.12.1.41	Yale University; Yale University	USA	Nursing	one author				
Siah, Chiew-Jiat; Lim, Fui-Ping; Lim, Ah-Ewe; Lau, Siew-Tiang; Tam, Wilson	Efficacy of team-based learning in knowledge integration and attitudes among year-one nursing students: A pre- and post-test study	COLLEGIAN	2019	10.1016/j.colegn.2019.05.003	National University of Singapore; National University of Singapore; National University of Singapore; National University of Singapore; National University of Singapore	Singapore	Nursing		one institution, many authors			
Mousavi, Mandana Akbarinejad; Amini, Mitra; Delavari, Somayeh; Seifi, Ali	Using Team-Based Learning to Teach Evidence-Based Medicine to First-Year Residents	ACTA FACULTATIS MEDICAE NAISSENSIS	2019	10.2478/afmnai-2019-0006	Shiraz University of Medical Science; Shiraz University of Medical Science; Iran University of Medical Sciences; University of Texas Health Science Center at San Antonio	USA+Iran	Medicine				Iran+USA	
McKeirnan, Kimberly C.; Bertsch, Taylor G.; Arnold, Ennifer; Panther, Shannon G.	Using Team-Based Learning to Train Student Pharmacists to Perform Tuberculin Skin Testing	AMERICAN JOURNAL OF PHARMACEUTICAL EDUCATION	2019		Washington State University; Washington State University; Washington State University; Washington State Pharm Assoc	USA	Pharmacy			USA - one state		
Anderson, Sarah M.; Geyer, Sarah; Cailor, Stephanie M.; Chen, Aleda M. H.	Impact of a team-based learning drug misuse education training program on student pharmacists' confidence	CURRENTS IN PHARMACY TEACHING AND LEARNING	2019	10.1016/j.cptl.2018.09.016	University of North Carolina Chapel Hill; Cedarville University; Cedarville University; Cedarville University	USA	Pharmacy			USA - multiple states		yes
Eksteen, M. J.	DOES TEAM-BASED LEARNING DEVELOP ESSENTIAL GENERIC SKILLS IN PHARMACY STUDENTS?	SOUTH AFRICAN JOURNAL OF HIGHER EDUCATION	2019	10.20853/33-1-1332	North West University - South Africa	South Africa	Pharmacy	one author				
Cevik, Arif Alper; ElZubeir, Margaret; Abu-Zidan, Fikri M.; Shaban, Sami	Team-based learning improves knowledge and retention in an emergency medicine clerkship	INTERNATIONAL JOURNAL OF EMERGENCY MEDICINE	2019	10.1186/s12245-019-0222-2	United Arab Emirates University; Tawam John Hopkins Hosp; United Arab Emirates University; United Arab Emirates University	UAE	Medicine			UAE		
Parthasarathy, Prabha; Apampa, Bugewa; Manfrin, Andrea	Perceptions of team-based learning using the Team-Based Learning Student Assessment Instrument: an exploratory analysis amongst pharmacy and biomedical students in the United Kingdom	JOURNAL OF EDUCATIONAL EVALUATION FOR HEALTH PROFESSIONS	2019	10.3352/jeehp.2019.16.23	Imperial College London; University of Sussex; University of Sussex	UK	Medicine		one institution, many authors			
Collins, Christine M.; Carrasco, Gonzalo A.; Lopez, Osvaldo J.	Participation in Active Learning Correlates to Higher Female Performance in a Pipeline Course for Underrepresented Students in Medicine	MEDICAL SCIENCE EDUCATOR	2019	10.1007/s40670-019-00794-2	Rowan University; Rowan University; Seton Hall Univ	USA	Medicine			USA - one state		
Carson, Ron; Mennenga, Heidi	Team-Based Learning and the Team-Based Learning Student Assessment Instrument (TBL-SAI): A Longitudinal Study of Master of Occupational Therapy Students' Changing Perceptions	AMERICAN JOURNAL OF OCCUPATIONAL THERAPY	2019	10.5014/ajot.2019.032623	Interim Home Hlth; Adventist University Hlth Sci; South Dakota State University	USA	Occupational medicine			USA - multiple states		
Carrasco, Gonzalo A.; Behling, Kathryn C.; Lopez, Osvaldo J.	Implementation of Team-Based Learning: a Tale of Two New Medical Schools	MEDICAL SCIENCE EDUCATOR	2019	10.1007/s40670-019-00815-0	Rowan University; Rowan University; Seton Hall Univ	USA	Medicine			USA - multiple states		
Norouzi, Zahra; Jafarnejad, Farzaneh; Khadivzadeh, Talaat; Esmaily, Habibollah; Hedjazi, Arya	Comparison of the effect of standardized patient-based training with team-based learning on the knowledge of midwifery students in providing services to victims of rape	JOURNAL OF EDUCATION AND HEALTH PROMOTION	2019	10.4103/jehp.jehp_237_18	Mashhad University Medical Science; Mashhad University Medical Science; Mashhad University Medical Science; Mashhad University Medical Science; Legal Med Org	Iran	Midwifery			Iran		
Wheeler, Sarah; Valentino, Alexa Sevin; Liston, Beth W.; Li, Junan; McAuley, James W.	A team-based learning approach to interprofessional education of medical and pharmacy students	CURRENTS IN PHARMACY TEACHING AND LEARNING	2019	10.1016/j.cptl.2019.07.010	Ohio State University; Ohio State University; Ohio State University; Ohio State University; Ohio State University	USA	Pharmacy		one institution, many authors			
Fernandes, Ashley K.; Ecklar, Pat; Rundell, Kristen; Luster, Gail; Cavalcanti, Maureen	Integrating Simulated Patients in TBL: a Strategy for Success in Medical Education	MEDICAL SCIENCE EDUCATOR	2019	10.1007/s40670-019-00727-z	Ohio State University; Ohio State University; Ohio State University; Ohio State University; Ohio State University	USA	Medicine		one institution, many authors			
Williams, Sarah Marie; Pereira-Reyes, Xiomara Lizeth; Korndorffer, Melanie L.	Student Learning of Radiological Anatomy Through Team-Based Learning Modules: Early Successes and Qualitative Analysis	MEDICAL SCIENCE EDUCATOR	2019	10.1007/s40670-019-00835-w	Louisiana State University; Universidad Nacional Autonoma de Honduras; Tulane University	USA+Honduras	Medicine				Honduras+USA	

Juliana, Koh Ying Yun; Rotgans, Jerome I.; Rajalingam, Preman; Gagnon, Paul; Low-Beer, Naomi; Schmidt, Henk G.	Effects of graded versus ungraded individual readiness assurance scores in team-based learning: a quasi-experimental study	ADVANCES IN HEALTH SCIENCES EDUCATION	2019	10.1007/s10459-019-09878-5	Nanyang Technological University; Nanyang Technological University; Nanyang Technological University; Nanyang Technological University; Nanyang Technological University; Erasmus University Rotterdam	Netherlands+Singapore	Medicine				Netherlands+Singapore	
Tulloch, Luis Gonzaga; Relan, Anju; Curello, Jennifer; Martin, Elise; Patel, Roma; Vijayan, Tara	Using Modified Team-Based Learning to Teach Antimicrobial Stewardship to Medical Students: One Institution's Approach	MEDICAL SCIENCE EDUCATOR	2019	10.1007/s40670-019-00804-3	Puget Sound Vet Affairs Healthcare Syst Hosp & Sp; University of California Los Angeles; University of California Los Angeles; University of California Los Angeles; University of California Los Angeles; Univ Pittsburg	USA	Medicine			USA - multiple states		
Ellis, Stanley K.; Quick, Charles M.; Graham, James	Faculty Peer Review as a Strategy to Assure Quality in a New Team-Based Learning Curriculum-a Single Institution Experience	MEDICAL SCIENCE EDUCATOR	2019	10.1007/s40670-019-00735-z	University of Arkansas Medical Sciences; University of Arkansas Medical Sciences; University of Arkansas Medical Sciences	USA	Medicine		one institution, many authors			
Wilson, Jennifer A.; Waghel, Rashi C.; Dinkins, Melissa M.	Flipped classroom versus a didactic method with active learning in a modified team-based learning self-care pharmacotherapy course	CURRENTS IN PHARMACY TEACHING AND LEARNING	2019	10.1016/j.cptl.2019.09.017	Wingate University; Wingate University; Wingate University	USA	Pharmacy		one institution, many authors			
Horst, Alexis; Schwartz, Brian D.; Fisher, Jenifer A.; Michels, Nicole; Van Winkle, Lon J.	Selecting and Performing Service-Learning in a Team-Based Learning Format Fosters Dissonance, Reflective Capacity, Self-Examination, Bias Mitigation, and Compassionate Behavior in Prospective Medical Students	INTERNATIONAL JOURNAL OF ENVIRONMENTAL RESEARCH AND PUBLIC HEALTH	2019	10.3390/ijerph16203926	Rocky Vista Univ; Rocky Vista Univ; Rocky Vista Univ; Rocky Vista Univ; Rocky Vista Univ	USA	Medicine		one institution, many authors			
Mohammad, Rima A.; Ellingrod, Vicki L.; Bleske, Barry E.	Implementing and evaluating virtual patient cases within a team-based learning pedagogy in a therapeutics course sequence	JOURNAL OF THE AMERICAN COLLEGE OF CLINICAL PHARMACY	2019	10.1002/jac5.1053	University of Michigan; University of Michigan; University of Michigan; University of New Mexico; University of Michigan	USA	Pharmacy			USA - one state		
Wiley, Rachel; Shelal, Zeena; Bernard, Carolyn; Urbauer, Diana; Toy, Eugene; Ramondetta, Lois	Team-Based Learning Module for Undergraduate Medical Education: a Module Focused on the Human Papilloma Virus to Increase Willingness to Vaccinate	JOURNAL OF CANCER EDUCATION	2019	10.1007/s13187-017-1311-7	University of Texas Health Science Center Houston; University of Texas Health Science Center Houston; University of Texas Health Science Center Houston; UUTMD Anderson Cancer Center; UUTMD Anderson Cancer Center; UUTMD Anderson Cancer Center; UUTMD Anderson Cancer Center	USA	Medicine		one institution, many authors			yes
Carrasco, Gonzalo A.; Behling, Kathryn C.; Lopez, Osvaldo J.	First year medical student performance on weekly team-based learning exercises in an infectious diseases course: insights from top performers and struggling students	BMC MEDICAL EDUCATION	2019	10.1186/s12909-019-1608-9	Rowan University; Rowan University; Rowan University	USA	Medicine			USA - one state		
Carrasco, Gonzalo A.; Behling, Kathryn C.; Lopez, Osvaldo J.	A Novel Grading Strategy for Team-Based Learning Exercises in a Hands-on Course in Molecular Biology for Senior Undergraduate Underrepresented Students in Medicine Resulted in Stronger Student Performance	BIOCHEMISTRY AND MOLECULAR BIOLOGY EDUCATION	2019	10.1002/bmb.21200	Rowan University; Rowan University; Rowan University; Rowan University	USA	Medicine			USA - one state		
Alnowaiser, Hadeel M.; Alwehaibi, Muaath; Mekkawy, Maha	THE EFFECT OF COMBINING TEAM BASED LEARNING (TBL) AND PROBLEM-BASED LEARNING (PBL) UPON THE STUDENTS' PERCEPTION TOWARD PROBLEM BASED LEARNING AT COLLEGE OF DENTISTRY, QASSIM UNIVERSITY: A CROSS SECTIONAL STUDY	INDO AMERICAN JOURNAL OF PHARMACEUTICAL SCIENCES	2019	10.5281/zenodo.2598064	Qassim University; Qassim University; Qassim University	Saudi Arabia	Pharmacy		one institution, many authors			
Volerman, Anna; Poeppelman, Rachel Stork	A pilot study of team-based learning in one-hour pediatrics residency conferences	BMC MEDICAL EDUCATION	2019	10.1186/s12909-019-1702-z	University of Chicago; University of Chicago; Nationwide Childrens Hospital	USA	Medicine			USA - multiple states		
DeMasi, Joseph; Harvan, Robin Ann; Luca, Magdalena	Online and In-class Team-Based Learning in Undergraduate Immunology: a Comparative Analysis	MEDICAL SCIENCE EDUCATOR	2019	10.1007/s40670-019-00814-1	Massachusetts Coll Pharm & Hlth Sci; Massachusetts Coll Pharm & Hlth Sci; Massachusetts Coll Pharm & Hlth Sci	USA	Medicine		one institution, many authors			
Hosny, Somaya; Ghaly, Mona; Eldesouki, Raghda; Hegazy, Ghada	Application of Modified Team-Based Learning Approach for Enhancing Undergraduate Medical Educational	MEDICAL SCIENCE EDUCATOR	2019	Egypt	Suez Canal University; Suez Canal University; Suez Canal University; Suez Canal University	Egypt	Medicine		one institution, many authors			

	Seminars											
Knight, Candace C.; Hamilton, Sharon H.	Getting Students to Value Leadership Early in the Nursing Curriculum: Innovation Makes It Possible	NURSING EDUCATION PERSPECTIVES	2019	10.1097/01.NEP.0000000000000351	University of Alabama Birmingham; University of Alabama Birmingham	USA	Nursing		one institution, many authors			
Gordon, Sarah K.; Trovinger, Sara; DeLellis, Teresa	Escape from the usual: Development and implementation of an 'escape room' activity to assess team dynamics	CURRENTS IN PHARMACY TEACHING AND LEARNING	2019	10.1016/j.cptl.2019.04.013	Univ Manchester; Univ Manchester; Univ Manchester	USA	Pharmacy		one institution, many authors			
Chen, Weichao; McCollum, Melanie A.; Bradley, Elizabeth B.; Nathan, Barnett R.; Chen, Donna T.; Worden, Mary Kate	Using Instrument-Guided Team Reflection and Debriefing to Cultivate Teamwork Knowledge, Skills, and Attitudes in Pre-Clerkship Learning Teams	MEDICAL SCIENCE EDUCATOR	2019	10.1007/s40670-018-00669-y	University of Virginia; University of Virginia; University of Virginia; Michigan State University; Michigan State University; University of Virginia; University of Virginia	USA	Medicine			USA - multiple states		
Chu, Tsung-Lan; Wang, Jeng; Monrouxe, Lynn; Sung, Yu-Chih; Kuo, Chen-li; Ho, Lun-Hui; Lin, Yueh-E	The effects of the flipped classroom in teaching evidence based nursing: A quasi-experimental study	PLOS ONE	2019	10.1371/journal.pone.0210606	Chang Gung Memorial Hospital; Chang Gung University of Science & Technology; Chang Gung University of Science & Technology; Chang Gung University of Science & Technology; Linkou Chang Gung Mem Hosp; Linkou Chang Gung Mem Hosp; Linkou Chang Gung Mem Hosp; Linkou Chang Gung Mem Hosp; Chang Gung University; National Taipei University of Nursing & Health Science (NTUNHS)	Taiwan	Nursing			Taiwan		
Malekigorji, Maryam	The Effect of Continued Team Randomization on Student's Perception and Performance in a Blended Team-Based Teaching Approach	EDUCATION SCIENCES	2019	10.3390/educsci9020102	Queens University Belfast	UK	Medicine	one author				
Ganotice, Fraide A., Jr.; Chan, Lap Ki	How can students succeed in computer-supported interprofessional team-based learning? Understanding the underlying psychological pathways using Biggs' 3P model	COMPUTERS IN HUMAN BEHAVIOR	2019	10.1016/j.chb.2018.09.029	Hong Kong Shue Yan University; University of Hong Kong	China	Interprofessional		one institution, many authors			
Sheakley, Maria L.; Bauler, Laura D.; Tanager, Claire L.; Newby, Dart	Student Perceptions of a Novel Approach to Promote Professionalism Using Peer Evaluation in a Team-Based Learning™ Setting: a Quality Improvement Project	MEDICAL SCIENCE EDUCATOR	2019	10.1007/s40670-019-00830-1	Western Michigan University; Western Michigan University; Western Michigan University; Western Michigan University	USA	Medicine		one institution, many authors			
Hibbard, Lisa	Case Studies for General Chemistry: Teaching with a Newsworthy Story	JOURNAL OF CHEMICAL EDUCATION	2019	10.1021/acs.jchemed.9b00420	Spelman College	USA	Medicine	one author				
Solai, LalithKumar K.; Kumar, Keerthana; Mulvaney, Elizabeth; Rosen, Daniel; Rodakowski, Juleen; Fabian, Tanya; Lingler, Jennifer H.; Reynolds, Charles F., III; Sewell, Daniel	Geriatric Mental Healthcare Training: A Mini-Fellowship Approach to Interprofessional Assessment and Management of Geriatric Mental Health Issues	AMERICAN JOURNAL OF GERIATRIC PSYCHIATRY	2019	10.1016/j.jagp.2019.04.018	University of Pittsburgh; University of Pittsburgh; University of Pittsburgh; University of Pittsburgh; University of Pittsburgh; University of Pittsburgh; University of Pittsburgh; Inst Med Boards; Inst Med Boards; University of California San Diego	USA	Interprofessional			USA - multiple states		
Rasmussen, Chad M.	A Small-Group Stratified-Learner Modification of Team-Based Learning (SGSL-TBL) for Resident Education	MEDICAL SCIENCE EDUCATOR	2019	10.1007/s40670-019-00762-w	Mayo Clinic	USA	Medicine	one author				
Ganguly, Anisha; Faulkner, Christopher; Sendelbach, Dorothy	Association of group composition diversity and performance outcomes in a pre-clerkship team-based learning program	MEDICAL TEACHER	2019	10.1080/0142159X.2019.1616682	University of Texas Southwestern Medical Center Dallas; University of Texas Southwestern Medical Center Dallas; University of Texas Southwestern Medical Center Dallas;	USA	Medicine		one institution, many authors			yes
Wasserman, Jason Adam; Kononova, Anastasia; Moldovan, Tudor; Cotten, Shelia R.	A Pilot Study Examining the Effects of Educational Setting and Stress on Multitasking Among Medical Students	MEDICAL SCIENCE EDUCATOR	2019	10.1007/s40670-018-00648-3	Oakland University; Michigan State University; Michigan State University; Michigan State University;	USA	Medicine			USA - one state		
Mookerjee, Anuradha Lele; Fischer, Bradford D.; Cavanaugh, Susan; Rajput, Vijay	Innovative curriculum: Integrating the bio-behavioral and social science principles across the LifeStages in basic science years	MEDICAL TEACHER	2019	10.1080/0142159X.2018.1457214	Rowan University; Rowan University; Rowan University; Ross Univ	USA	Medicine			USA - multiple states		
Earl, Grace L.; Harris, Elizabeth M.; Dave, Mohak; Estriplet-Jiang, Jessica	Implementing a health literacy module fostering patient-centered written communication in a cardiovascular prevention elective course	CURRENTS IN PHARMACY TEACHING AND LEARNING	2019	10.1016/j.cptl.2019.03.008	Univ Sci - Philadelphia; Univ Sci - Philadelphia; NewYork-Presbyterian Hospital; Essex Cty Coll	USA	Pharmacy			USA - multiple states		

Lin, Jian-Wei	The impact of team-based learning on students with different self-regulated learning abilities	JOURNAL OF COMPUTER ASSISTED LEARNING	2019	10.1111/jcal.12382	Chien Hsin University of Science & Technology	Taiwan	Medicine	one author				
Burgess, Annette; Matar, Elie; Neuen, Brendon; Fox, Greg J.	A longitudinal faculty development program: supporting a culture of teaching	BMC MEDICAL EDUCATION	2019	10.1186/s12909-019-1832-3	University of Sydney; University of Sydney; University of Sydney; University of New South Wales Sydney; George Institute for Global Health; George Institute for Global Health; Royal Prince Alfred Hospital; University of Sydney	Australia	Medicine			Australia		
LoSavio, Stefanie T.; Dillon, Kirsten H.; Murphy, Robert A.; Goetz, Karen; Houston, Falesha; Resick, Patricia A.	Using a Learning Collaborative Model to Disseminate Cognitive Processing Therapy to Community-Based Agencies	BEHAVIOR THERAPY	2019	10.1016/j.beth.2018.03.007	Duke University; Duke University; Duke University; Duke University; Duke University; Duke University; Durham VA Medical Center; Ctr Child & Family Hlth	USA	Medicine			USA - one state		
Lerchenfeldt, Sarah; Eng, Marty	A Survey of Health Sciences Faculty Practices and Attitudes Regarding the Peer Feedback Component of Team-Based Learning	MEDICAL SCIENCE EDUCATOR	2019	10.1007/s40670-019-00816-z	Oakland University; Cedarville University	USA	Medicine			USA - multiple states		
Farland, Michelle Z.; Feng, Xiaoying; Behar-Horenstein, Linda S.; Beck, Diane E.	Impact of Team Formation Method on Student Team Performance Across Multiple Courses Incorporating Team-based Learning	AMERICAN JOURNAL OF PHARMACEUTICAL EDUCATION	2019	10.5688/ajpe7030	University of Florida; University of Florida; University of Florida; University of Florida	USA	Pharmacy			USA - multiple states		
AlSheikh, Mona Hmoud; Iqbal, Muhammad Zafar	Student Perceptions Regarding Group Learning Activities in a Hybrid Medical Curriculum	MEDICAL SCIENCE EDUCATOR	2019	10.1007/s40670-019-00817-y	Imam Abdulrahman Bin Faisal University; Imam Abdulrahman Bin Faisal University	Saudi Arabia	Medicine		one institution, many authors			
Mansoor, Memoona; Aly, Syed Moyn; Javaid, Arshad	Effect of Team-based Learning on Second Year Students' Academic Performance	JCPSP-JOURNAL OF THE COLLEGE OF PHYSICIANS AND SURGEONS PAKISTAN	2019	10.29271/jcpsp.2019.09.860	Islamabad Med & Dent Coll; Jinnah Sindh Medical University - Pakistan; Shifa College of Medicine	Pakistan	Medicine			Pakistan		
Aliakbarzadeh Arani, Zahra; Haji Mohammad Hoseini, Mahsa; Ghanbari Afra, Leila; Mohammadzade, Maede	The effect of teaching on team-based learning and group discussion on learning and academic motivation of operating room students in the technology of gastrointestinal surgery lesson	JOURNAL OF NURSING AND MIDWIFERY SCIENCES	2019	10.4103/JNMS.JNMS_61_18	Qom Univ Med Sci; Qom Univ Med Sci; Qom Univ Med Sci; Univ Social Welf & Rehabil Sci; Islamic Azad University	Iran	Nursing			Iran		
Janke, Kristin K.; Bechtol, Robert A.; James, Stephanie; Lepp, Gardner; Moote, Rebecca; Clapp, Peter	Determining Indicators of High-Quality Application Activities for Team-Based Learning	AMERICAN JOURNAL OF PHARMACEUTICAL EDUCATION	2019		University of Minnesota Twin Cities; University of Minnesota Twin Cities; Regis University; Regis University; University of Minnesota Duluth; University of Texas Austin	USA	Pharmacy			USA - multiple states		
Lin, Yulia; Tilokee, Everad; Charge, Sophie; Alam, Asim; Cserti-Gazdewich, Christine; Lau, Wendy; Lee, Christie; Lieberman, Lani; Nixon, Paula; Owens, Wendy; Pavenski, Katerina; Pendergrast, Jacob; Saidenberg, Elianna; Shehata, Nadine; Skeate, Robert; Yi, Qi-Long; Conrad, David; Dudebout, Jill; Hsia, Cyrus C.; Murphy, Michael; Prokopchuk-Gauk, Oksana; Shah, Akshay; Solh, Ziad; Trudeau, Jacqueline; Zeller, Michelle P.; Callum, Jeannie	Transfusion Camp: a prospective evaluation of a transfusion education program for multispecialty postgraduate trainees	TRANSFUSION	2019	10.1111/trf.15284	University of Toronto; Sunnybrook Health Science Center; Sunnybrook Research Institute; University of Toronto; University of Toronto; University of Toronto; University of Toronto; University of Toronto; North York General Hospital; University of Toronto; University Health Network Toronto; University of Toronto; Hospital for Sick Children (SickKids); University of Toronto; Saint Michaels Hospital Toronto; University of Toronto; Sinai Health System Toronto; Lunenfeld Tanenbaum Research Institute; Canadian Blood Services; University of Ottawa; Ottawa Hospital Research Institute; Dalhousie University; Queens University - Canada; Western University (University of Western Ontario); University of Saskatchewan; University of British Columbia; McMaster University; Canadian Blood Services	Canada +UK	Medicine				UK+Canada	
Das, Saswati; Nandi, Kajal; Baruah, Priyanki; Sarkar, Sajib K.; Goswami, Binita; Koner, Bidhan C.	Is learning outcome after team based learning influenced by gender and academic standing?	BIOCHEMISTRY AND MOLECULAR BIOLOGY EDUCATION	2019	10.1002/bmb.21197	Maulana Azad Medical College; Maulana Azad Medical College; Maulana Azad Medical College; Maulana Azad Medical College; Maulana Azad Medical College; Maulana Azad Medical College	India	Medicine		one institution, many authors			
Maeno, Takami; Haruta, Junji; Takayashiki, Ayumi; Yoshimoto, Hisashi; Goto, Ryohei; Maeno, Tetsuhiro	Interprofessional education in medical schools in Japan	PLOS ONE	2019	10.1371/journal.pone.0210912	University of Tsukuba; University of Tsukuba; University of Tsukuba; University of Tsukuba; University of Tsukuba; University of Tsukuba	Japan	Medicine		one institution, many authors			
Salih, Karimeldin Mohamed Ali; AL-Shahrani, Abdullah M.; Eljac, Ibrahim Awad; Abbas, Mohammed	Perception of Faculty Members of Regional Medical School Toward Faculty Development Program	SUDAN JOURNAL OF MEDICAL SCIENCES	2019	10.18502/sjms.v14i3.5205	University of Bisha; University of Bisha; University of Bisha; University of Bahri; University of Bisha; University of Bisha; Kassala Univ	Saudi Arabia+ Sudan	Medicine				Saudi Arabia+Sudan	

Singh, Keerti; Bharatha, Ambadasu; Sa, Bidyadhar; Adams, Oswald Peter; Majumder, Md Anwarul Azim	Teaching anatomy using an active and engaging learning strategy	BMC MEDICAL EDUCATION	2019	10.1186/s12909-019-1590-2	University West Indies Cave Hill Campus; University West Indies Cave Hill Campus; University West Indies Cave Hill Campus; University West Indies Cave Hill Campus; University West Indies Saint Augustine	Trinidad Tobago +Barbados	Medicine		one institution, many authors			
Burgess, Annette; Haq, Inam; Bleasel, Jane; Roberts, Chris; Garsia, Roger; Randal, Nicholas; Mellis, Craig	Team-based learning (TBL): a community of practice	BMC MEDICAL EDUCATION	2019	10.1186/s12909-019-1795-4	University of Sydney; University of Sydney; University of Sydney; University of Sydney; University of Sydney; University of Sydney; University of Sydney	Australia	Medicine		one institution, many authors			
Klincova, Martina; Harazim, Hana; Schwarz, Daniel; Kosinova, Martina; Smekalova, Olga; Stourac, Petr	What Can Be Achieved With Motivation-Based Teaching of Medical Students? A Monocentric Retrospective Audit of Retention Among Highly Motivated Graduates Who Underwent the Learning-by-Doing Concept in Anesthesiology and Intensive Care Medicine	JMIR SERIOUS GAMES	2019	10.2196/10155	Masaryk University Brno; Masaryk University Brno; Masaryk University Brno; Masaryk University Brno; Masaryk University Brno; Charles University Prague	Czech Republic	Medicine			Chech Republic		
Kaminski, Ashley D.; Babbitt, Katherine M.; McCarthy, Mary C.; Markert, Ronald J.; Roelle, Melissa P.; Parikh, Priti P.	Team-Based Learning in the Surgery Clerkship: Impact on Student Examination Scores, Evaluations, and Perceptions	JOURNAL OF SURGICAL EDUCATION	2019	10.1016/j.jsurg.2018.07.031	Wright State University Dayton; Wright State University Dayton; Wright State University Dayton; Wright State University Dayton; Wright State University Dayton; Wright State University Dayton	USA	Medicine		one institution, many authors			yes
El-Banna, Majeda M.; Whitlow, Malinda; McNelis, Angela M.	Improving Pharmacology Standardized Test and Final Examination Scores Through Team-Based Learning	NURSE EDUCATOR	2020	10.1097/NNE.0000000000000671	George Washington University; George Washington University; George Washington University	USA	Nursing		one institution, many authors			
Burgess, Annette; Bleasel, Jane; Hickson, John; Guler, Ceren; Kalman, Eszter; Haq, Inam	Team-based learning replaces problem-based learning at a large medical school	BMC MEDICAL EDUCATION	2020	10.1186/s12909-020-02362-4	University of Sydney; University of Sydney; University of Sydney; University of Sydney; University of Sydney; University of Sydney	Australia	Medicine		one institution, many authors			
Rezende, Alice B.; de Oliveira, Andre G. F.; Vale, Thiago C.; Teixeira, Luciana A. S.; Lima, Alba R. A.; Lucchetti, Alessandra L. G.; Lucchetti, Giancarlo; Tibirica, Sandra H. C.; Ezequiel, Oscarina S.	Comparison of Team-Based Learning versus Traditional Lectures in Neuroanatomy: Medical Student Knowledge and Satisfaction	ANATOMICAL SCIENCES EDUCATION	2020	10.1002/ase.1926	Universidade Federal de Juiz de Fora; Universidade Federal de Juiz de Fora; Universidade Federal de Juiz de Fora; Universidade Federal do Triangulo Mineiro; UFaculdade de Medicina de Sao Jose do Rio Preto (FAMERP); Universidade Federal de Juiz de Fora; Universidade Federal de Juiz de Fora; Universidade Federal de Juiz de Fora; Universidade Federal de Juiz de Fora; Universidade Federal de Juiz de Fora;	Brazil	Medicine			Brazil		
Liu, Hua; Mi, Xu Feng; Huang, Zi Zhan; Heng, Boon Chin; Shen, Wei Liang	Challenges and strategies in developing team-based learning in Chinese medical education	MEDICAL TEACHER	2020	10.1080/0142159X.2020.1801995	Zhejiang University; Zhejiang University; Zhejiang University; Zhejiang University; Peking University	China	Medicine			China		
Ueda, Masahiro; Takagaki, Nobumasa; Onda, Mitsuko; Arakawa, Yukio; Shoji, Masaki; Ohmori, Shiho; Shimizu, Tadashi	Introduction of Team-based Learning to Evidence-based Medicine Educational Course for Pharmacy Students	YAKUGAKU ZASSHI-JOURNAL OF THE PHARMACEUTICAL SOCIETY OF JAPAN	2020		Hyogo Medical University; Setsunan University; Senshunkai Hosp; Osaka Medical & Pharmaceutical University; Osaka University of Pharmaceutical Sciences; Osaka University of Pharmaceutical Sciences; Osaka University of Pharmaceutical Sciences; Hyogo Medical University;	Japan	Pharmacy			Japan		
Randall, Sue; Crawford, Tonia; River, Jo	Us and them: The experience of international nursing students engaged in team based learning: A qualitative descriptive study	NURSE EDUCATION TODAY	2020	10.1016/j.nedt.2020.104527	University of Sydney; University of Sydney; University of Sydney	Australia	Nursing		one institution, many authors			
Ihm, Jungjoon; Shin, Yongmin; Seo, Deog-Gyu	Did Clinical Reasoning and Knowledge Questions During Team-Based Learning Enhance Dental Students' Performance in Esthetic Dentistry?	JOURNAL OF DENTAL EDUCATION	2020	10.21815/JDE.019.191	Seoul National University; Seoul National University; Seoul National University	South Korea	Dentistry		one institution, many authors			
Sakamoto, Sabrina Ramires; Queiroz Dell'Acqua, Magda Cristina; Fernandes Abbade, Luciana Patricia; Caldeira, Silvia Maria; Benato Fusco, Suzimar de Fatima; Garcia de Avila, Marla Andreia	Team-Based Learning: a randomized clinical trial in undergraduate nursing	REVISTA BRASILEIRA DE ENFERMAGEM	2020	10.1590/0034-7167-2018-0621	Universidade Estadual Paulista; Universidade Estadual Paulista; Universidade Estadual Paulista; Universidade Estadual Paulista; Universidade Estadual Paulista; Universidade Estadual Paulista	Brazil	Nursing		one institution, many authors			
Graham, Jane; Hayes, Conrad; Pendry, Kate	Can Team-Based Learning (TBL) Be Used to Deliver Postgraduate Education in Transfusion Medicine for UK Physicians?	MEDICAL SCIENCE EDUCATOR	2020	10.1007/s40670-019-00844-9	Univ Hosp North Midlands NHS Trust; Keele University; Keele University; Manchester Univ NHS Fdn Trust	UK	Medicine			England		

Roh, Young Sook; Kim, Sang Suk; Park, Sunah; Ahn, Jung-Won	Effects of a Simulation With Team-Based Learning on Knowledge, Team Performance, and Teamwork for Nursing Students	CIN-COMPUTERS INFORMATICS NURSING	2020	10.1097/CIN.0000000000000628	Chung Ang University; Chung Ang University; Chung Ang University; Gangneung-Wonju National University	South Korea	Nursing			South Korea		
Lochner, Lukas; Wieser, Heike; Oberhoeller, Gabi; Ausserhofer, Dietmar	Interprofessional team-based learning in basic sciences: students' attitude and perception of communication and teamwork	INTERNATIONAL JOURNAL OF MEDICAL EDUCATION	2020	10.5116/ijme.5f5b.24e3	Coll Healthcare Profess; Coll Healthcare Profess; Coll Healthcare Profess; Laimburg Res Ctr	Italy	Interprofessional			Italy		
Lewis, Chrystal L.; Estis, Julie M.	Using Team-Based Learning to Teach Human Subjects Research Ethics	MEDICAL SCIENCE EDUCATOR	2020	10.1007/s40670-019-00846-7	University of South Alabama; University of South Alabama	USA	Medicine		one institution, many authors			
Jalali, Alireza; Jeong, Dahn; Sutherland, Stephanie	Implementing a Competency-Based Approach to Anatomy Teaching: Beginning With the End in Mind	JOURNAL OF MEDICAL EDUCATION AND CURRICULAR DEVELOPMENT	2020	10.1177/2382120520907899	University of Ottawa; University of Ottawa; Ottawa Hospital Research Institute	Canada	Medicine			Canada		
Ari, Mim; Venci, Julie A.; Kulasekaran, Vishnu; Adams, Jennifer E.	Team-Based Learning in a Longitudinal Integrated Clerkship: an Opportunity for Integrated Multidisciplinary Learning and Curricular Focus on the Underserved	MEDICAL SCIENCE EDUCATOR	2020	10.1007/s40670-019-00856-5	University of Chicago; University of Colorado; University of Colorado; University of Colorado; Denver Health Medical Center; Denver Health Medical Center; Denver Health Medical Center	USA	Medicine			USA - multiple states		
Tweddell, Simon	Evaluating the introduction of team - based learning in a pharmacy consultation skills module	PHARMACY EDUCATION	2020	10.46542/pe.2020.201.151157	University of Bradford	UK	Pharmacy	one author				
Yang, Lishan; Rajalingam, Preman	Are Two Teachers Better than One? Team Teaching in TBL	MEDICAL SCIENCE EDUCATOR	2020	10.1007/s40670-019-00828-9	Nanyang Technological University; Nanyang Technological University	Singapore	Medicine		one institution, many authors			
Seo, Yukyeong; Roh, Young Sook	Effects of pressure ulcer prevention training among nurses in long-term care hospitals	NURSE EDUCATION TODAY	2020	10.1016/j.nedt.2019.104225	Chung Ang University; Chung Ang University	South Korea	Nursing		one institution, many authors			
Anderson, Hana; Sweeney, Colleen; Perry, Ross; Vaquero, Jorge; Ison, Hannah	Patient-Centered Team-Based Learning in Pre-Clinical Curriculum Supporting the Application of Knowledge to Real-World Clinical Experience	MEDICAL SCIENCE EDUCATOR	2020	10.1007/s40670-019-00872-5	University of California Davis; University of California Davis; University of California Davis; University of California Davis; Stanford Ctr Inherited Cardiovasc Dis	USA	Medicine			USA - one state		
Kim, Soomi; Kim, Chul-Gyu	Effects of an Electrocardiography Training Program: Team-Based Learning for Early-Stage Intensive Care Unit Nurses	JOURNAL OF CONTINUING EDUCATION IN NURSING	2020	10.3928/00220124-20200317-07	Chungbuk National University; Chungbuk National University	South Korea	Nursing		one institution, many authors			
Lancellotti, Domingo; Abarca, Alejandro; Jorquera, Javiera; Lobos, Camila; Aguilera, Diego; Sanchez, Nelly	Evaluation of team-based learning methodology for teaching statistics to medical students	REVISTA MEDICA DE CHILE	2020		Universidad Catolica del Norte; Universidad Catolica del Norte; Universidad Catolica del Norte; Universidad Catolica del Norte; Universidad Catolica del Norte; Universidad Catolica del Norte	Chile	Medicine			Chile		
Chitkara, Maribeth B.; Pongvitayapanu, Richard; Lu, Wei-Hsin	Integrating iPads into Team-Based Learning in the Pediatrics Clerkship: Do They Provide Any Value?	JOURNAL OF MEDICAL EDUCATION AND CURRICULAR DEVELOPMENT	2020	10.1177/2382120520957645	State University of New York (SUNY) Stony Brook; State University of New York (SUNY) Stony Brook; State University of New York (SUNY) Stony Brook	USA	Medicine		one institution, many authors			
Mogali, Sreenivasulu Reddy; Rotgans, Jerome, I; Rosby, Lucy; Ferenczi, Michael Alan; Beer, Naomi Low	Summative and Formative Style Anatomy Practical Examinations: Do They Have Impact on Students' Performance and Drive for Learning?	ANATOMICAL SCIENCES EDUCATION	2020	10.1002/ase.1931	Nanyang Technological University; Nanyang Technological University; Nanyang Technological University; Nanyang Technological University; Nanyang Technological University; Erasmus University Rotterdam	Netherlands+Singapore	Medicine				Netherlands+Singapore	
Smeby, Susanne Skjervold; Lillebo, Borge; Slordahl, Tobias S.; Berntsen, Erik Magnus	Express Team-Based Learning (eTBL): A Time-Efficient TBL Approach in Neuroradiology	ACADEMIC RADIOLOGY	2020	10.1016/j.acra.2019.04.022	Norwegian University of Science & Technology (NTNU); Norwegian University of Science & Technology (NTNU); Norwegian University of Science & Technology (NTNU); Norwegian University of Science & Technology (NTNU); Levanger Hosp; Trondheim Reg & Univ Hosp	Norway	Medicine			Norway		
Daou, Dayane; Sabra, Ramzi; Zgheib, Nathalie K.	Factors That Determine the Perceived Effectiveness of Peer Feedback in Collaborative Learning: a Mixed Methods Design	MEDICAL SCIENCE EDUCATOR	2020	10.1007/s40670-020-00980-7	American University of Beirut; American University of Beirut; American University of Beirut	Lebanon	Medicine		one institution, many authors			
Hamada, Shuhei; Haruta, Junji; Maeno, Takami; Maeno, Tetsuhiro; Suzuki, Hideo; Takayashiki, Ayumi; Inada, Haruhiko; Naito, Takahiro; Tomita, Mika; Kanou, Naomi; Baba, Takeshi	Effectiveness of an interprofessional education program using team-based learning for medical students: A randomized controlled trial	JOURNAL OF GENERAL AND FAMILY MEDICINE	2020	10.1002/jgf2.284	University of Tsukuba; University of Tsukuba; University of Tsukuba; University of Tsukuba; University of Tsukuba; University of Tsukuba; Johns Hopkins University; Teikyo Univ Sci; Ibaraki Prefectural Univ Hlth Sci	USA+Japan	Medicine				Japan+USA	

Al-Hammouri, Mohammed Munther; Rababah, Jehad A.; Rowland, Michael L.; Tetreault, Allison Serra; Aldalaykeh, Mohammed	Does a novel teaching approach work? A Students' perspective	NURSE EDUCATION TODAY	2020	10.1016/j.nedt.2019.104229	Jordan University of Science & Technology; Jordan University of Science & Technology; Jordan University of Science & Technology; University of Kentucky; University of Kansas	USA+Jordan	Nursing				Jordan+USA	
Mayel, Masoud; Hoseini, Seyed-Hosein; Teimouri, Ali; Shabouni, Zeinab; Rayat-Dost, Esmaeil; Foroughian, Mahdi	Teaching Approach to Tachycardia and Bradycardia in Medical Students: A Quasi-Experimental Study to Compare Team-Based Learning and Lecture Method	ADVANCED JOURNAL OF EMERGENCY MEDICINE	2020	10.22114/ajem.v0i0.167	Kerman University of Medical Sciences; Kerman University of Medical Sciences; Mashhad University Medical Science; Mashhad University Medical Science; Kerman University of Medical Sciences; Jahrom Univ Med Sci	Iran	Medicine			Iran		
Ibrahim, Mutasim E.	Team-based learning student assessment instrument (TBL-SAI) for assessing students' acceptance of TBL in a Saudi medical school. Psychometric analysis and differences by academic year	SAUDI MEDICAL JOURNAL	2020	10.15537/smj.2020.5.25054	University of Bisha	Saudi Arabia	Medicine	one author				
Nanda, Manpreet Singh; Singh, Rajdeep; Kotwal, Sonika	Impact of Introduction of Team Based Learning on Third Year MBBS Undergraduate Students	JOURNAL OF EVOLUTION OF MEDICAL AND DENTAL SCIENCES-JEMDS	2020	10.14260/jemds/2020/794	Maharishi Markandeshwar University; Maharishi Markandeshwar University; Maharishi Markandeshwar University	India	dentistry		one institution, many authors			
Bridges, Susan M.; Chan, Lap Ki; Chen, Julie Y.; Tsang, Joyce P. Y.; Ganotice, Fraide A.	Learning environments for interprofessional education: A micro-ethnography of sociomaterial assemblages in team-based learning	NURSE EDUCATION TODAY	2020	10.1016/j.nedt.2020.104569	University of Hong Kong; University of Hong Kong; Macau University of Science & Technology; University of Hong Kong; University of Hong Kong	USA+China	Nursing				USA+China	
Khalafalla, Farid G.; Covarrubias, Kenya; Fesperman, Madison; Eichmann, Kelly; VanGarsse, Anne; Ofstad, William	Enhancing nutrition and lifestyle education for healthcare professional students through an interprofessional, team-based training program	CURRENTS IN PHARMACY TEACHING AND LEARNING	2020	10.1016/j.cptl.2020.07.017	California Health Sciences University (CHSU); California Health Sciences University (CHSU); California Health Sciences University (CHSU); Clovis Unified Sch Dist; Natl Univ - Fresno; California Health Sciences University (CHSU); California Health Sciences University (CHSU)	USA	Pharmacy			USA - one state		
Bauler, Timothy J.; Sheakley, Maria L.; Ho, Arlene	Use of the Team-Based Learning Readiness Assessment Test as a Low-Stakes Weekly Summative Assessment to Promote Spaced and Retrieval-Based Learning	MEDICAL SCIENCE EDUCATOR	2020	10.1007/s40670-019-00826-x	Western Michigan University; Western Michigan University; Western Michigan University	USA	Medicine		one institution, many authors			
Huang, Cheng-Yi; Wang, Ya-huei	Toward an Integrative Nursing Curriculum: Combining Team-Based and Problem-Based Learning with Emergency-Care Scenario Simulation	INTERNATIONAL JOURNAL OF ENVIRONMENTAL RESEARCH AND PUBLIC HEALTH	2020	10.3390/ijerph17124612	Chung Shan Medical University; Chung Shan Medical University	Taiwan	Nursing		one institution, many authors			
Fernandes, Ashley K.; Wilson, Sheria; Kasick, Rena; Humphrey, Lisa; Mahan, John; Spencer, Sandra	Team-Based Learning in Bioethics Education: Creating a Successful Curriculum for Residents in an Era of Curricular Squeeze	MEDICAL SCIENCE EDUCATOR	2020	10.1007/s40670-019-00836-9	Nationwide Childrens Hospital; Nationwide Childrens Hospital; Nationwide Childrens Hospital; Nationwide Childrens Hospital; Nationwide Childrens Hospital; Nationwide Childrens Hospital	USA	Medicine		one institution, many authors			
Levine, Ruth E.; Hsieh, Peggy; Kelly, P. Adam; Carchedi, Lisa; Gibson, Jennifer; Haidet, Paul; Koles, Paul; Pershern, Lindsey; Schatte, Dawnelle; Talley, Brenda; Wolf, Dwight; Thompson, Britta	The Facilitator Instrument for Team-Based Learning (FIT)	TEACHING AND LEARNING IN MEDICINE	2020	10.1080/10401334.2019.1646653	University of Texas Medical Branch Galveston; University of Texas Medical Branch Galveston; McGovern Medical School; Tulane University; Univ Texas Southwestern; Tulane University; Commonwealth System of Higher Education (PCSHE); Pennsylvania State University; Pennsylvania State University; Wright State University Dayton; Univ Texas Southwestern; McGovern Medical School; University of Texas Health Science Center at San Antonio	USA	Medicine			USA - multiple states		yes
Eladl, Mohamed A.; Jarrahi, Abbas	Using practical-based team-based learning (PTBL) as a tool for providing an immediate feedback to the students during Anatomy Education	EUROPEAN JOURNAL OF ANATOMY	2020		University of Sharjah; University of Sharjah	UAE	Medicine		one institution, many authors			
Nuebel, Esther; Nowinski, Sara M.; Hemmis, Casey W.; Lindsley, Janet E.	A Curriculum Design and Teaching Experience Created by and for Bioscience Postdoctoral Fellows in a Medical School	MEDICAL SCIENCE EDUCATOR	2020	10.1007/s40670-019-00889-w	University of Utah; University of Utah; University of Utah; University of Utah; Howard Hughes Medical Institute	USA	Medicine			USA - multiple states		
Mastel-Smith, Beth; Kimzey, Michelle; Garner, Jennifer; Shoair, Osama A.; Stocks, Eric; Wallace, Takova	Dementia care boot camp: interprofessional education for healthcare students	JOURNAL OF INTERPROFESSIONAL CARE	2020	10.1080/13561820.2019.1696287	University of Texas at Tyler; Texas Christian University; Tyler Jr Coll; University of Texas at Tyler; University of Texas at Tyler; University of Texas at Tyler	USA	Interprofessional			USA - one state		
Schmidt, Henk G.; Mamede, Silvia	How cognitive psychology changed the face of medical education research	ADVANCES IN HEALTH SCIENCES EDUCATION	2020	10.1007/s10459-020-10011-0	Erasmus University Rotterdam; Erasmus University Rotterdam	Netherlands	Medicine		one institution, many authors			

Wang, Xinjue Rachel; Hillier, Tracey; Oswald, Anna; Lai, Hollis	Patterns of performance in students with frequent low stakes team based learning assessments: Do students change behavior?	MEDICAL TEACHER	2020	10.1080/0142159X.2019.1670339	University of Alberta; University of Alberta; University of Alberta; University of Alberta	Canada	Medicine		one institution, many authors			
Hanna, Lezley-Anne; McReynolds, Meadhbh; Hall, Maurice; Hanna, Alan	Final year pharmacy students' opinions on teamwork: A questionnaire-based study from a UK School of Pharmacy	PHARMACY EDUCATION	2020		Queens University Belfast; Queens University Belfast; Queens University Belfast; Queens University Belfast	Ireland	Pharmacy		one institution, many authors			
Pek, Jen Heng; Chia, Wen Jie Dennis; Kaliannan, Sathya; Wong, Yin Theng; Chan, Kim Poh	Teaching ultrasound guided femoral nerve block in the emergency department	MEDICAL ULTRASONOGRAPHY	2020	10.11152/mu-2112	SingHealth; SingHealth; SingHealth; SingHealth; SingHealth	Singapore	Medicine		one institution, many authors			
Mlika, Mona; Charfi, Rim; Cheikhrouhou, Sarah; Mezni, Faouzi	About the association of a lecture-based learning and team-based learning in a pathology course	ANNALES DE PATHOLOGIE	2020	10.1016/j.annpat.2019.11.004	Universite de Tunis-El-Manar; Universite de Tunis-El-Manar; Universite de Tunis-El-Manar; Hopital Habib Thameur	Tunisia	Medicine			Tunisia		
Goncalves Pires, Elda Maria Stafuzza; Daniel-Filho, Durval Anibal; de Nooijer, Jascha; Dolmans, Diana H. J. M.	Collaborative learning: Elements encouraging and hindering deep approach to learning and use of elaboration strategies	MEDICAL TEACHER	2020	10.1080/0142159X.2020.1801996	Fac Israelita Ciencias Saude Albert Einstein FICS; Fac Israelita Ciencias Saude Albert Einstein FICS; Fac Israelita Ciencias Saude Albert Einstein FICS; Fac Israelita Ciencias Saude Albert Einstein FICS	Brazil	Medicine		one institution, many authors			
Karandish, Maryam	Comparison of individual and team readiness assurance tests in a modified team based learning method	JOURNAL OF DENTAL EDUCATION	2020	10.1002/jdd.12190	Shiraz University of Medical Science	Iran	dentistry	one author				
Fettkether, Rebekah M. Jackowski; Hall-Lipsy, Elizabeth A.	Using a longitudinal case-based approach to teach population health and disease prevention	CURRENTS IN PHARMACY TEACHING AND LEARNING	2020	10.1016/j.cptl.2020.04.024	Midwestern University; University of Arizona	USA	Pharmacy			USA - one state		
Franz, Thad; Cailor, Stephanie; Chen, Aleda M. H.; Thornton, Phillip; Norfolk, MeiLing	Improvement of student confidence and competence through a self-care skills multi-course integration	CURRENTS IN PHARMACY TEACHING AND LEARNING	2020	10.1016/j.cptl.2019.12.022	Cedarville University; Cedarville University; Cedarville University; Cedarville University; Mint Hill Pharm	USA	Pharmacy			USA - multiple states		
Currey, Judy; Sprogis, Stephanie K.; Burdeu, Gabby; Story, Ian; Considine, Julie; O'Donnell, Marcus; Gentle, Alex; Backhouse, Simon; Oldland, Elizabeth	Stakeholder acceptance of digital team-based learning	NURSE EDUCATION IN PRACTICE	2020	10.1016/j.nepr.2020.102833	Deakin University; Deakin University; Deakin University; Deakin University; Deakin University; Deakin University; Deakin University; Deakin University; Deakin University; Eastern Hlth Partnership; Eastern Hlth Partnership	Australia	Nursing			Australia		
Crisafio, Anthony; Cho, Stephanie H.	Impact of Varying Active Learning Time on Student Performance on a Standardized Exam in the Psychiatry Clerkship	ACADEMIC PSYCHIATRY	2020	10.1007/s40596-019-01147-2	St Georges Univ; University of Southern California	USA	Medicine		one institution, many authors			
Sibomana, Isaie; Karenzi, Irenee David; Niyongombwa, Irenee; Byiringiro, Jean Claude; Gashegu, Julien; Ntirenganya, Faustin	Use of Student-Generated Multiple Choice Questions to Enhance Team-Based Learning of Anatomy at the University of Rwanda	ADVANCES IN MEDICAL EDUCATION AND PRACTICE	2020	10.2147/AMEP.S274298	University of Rwanda; University of Rwanda; University of Rwanda; University of Rwanda; University of Rwanda; University of Rwanda	Rwanda	Medicine			Rwanda		
Thomas, Aaron O.; Voils, Stacy A.; Childs-Kean, Lindsey	Modification of the self-regulated strategy inventory-self report for blended and team-based learning	CURRENTS IN PHARMACY TEACHING AND LEARNING	2020	10.1016/j.cptl.2020.04.021	University of Florida; University of Florida; University of Florida	USA	Pharmacy		one institution, many authors			
Brittain, Kristy; Spies, Alan; Worrall, Cathy	Leader Academy: A layered approach to learning leadership	CURRENTS IN PHARMACY TEACHING AND LEARNING	2020	10.1016/j.cptl.2019.12.012	Medical University of South Carolina; Medical University of South Carolina; GiANT Worldwide	USA	Pharmacy		one institution, many authors			
Holloway, W. Blake, II; Grove, Jordan; Tyson, Anna; Rochford, Laura; Day, Lee; Resuehr, David	Utilization of a Co-enrolled Course Structure for Point-of-Care Ultrasound Training in the Undergraduate Medical Education Setting	MEDICAL SCIENCE EDUCATOR	2020	10.1007/s40670-019-00831-0	University of Alabama Birmingham; University of Alabama Birmingham; University of Alabama Birmingham; University of Alabama Birmingham; University of Alabama Birmingham; University of Alabama Birmingham	USA	Medicine		one institution, many authors			
Hennrikus, Eileen F.; Skolka, Michael P.; Hennrikus, Nicholas	Social Constructivism in Medical School Where Students Become Patients with Dietary Restrictions	ADVANCES IN MEDICAL EDUCATION AND PRACTICE	2020	10.2147/AMEP.S259727	Pennsylvania State University; Mayo Clinic; Chula Vista Learning Community Charter Sch	USA	Medicine			USA - multiple states		
Koh, Ying Yun Juliana; Schmidt, Henk G.; Low-Beer, Naomi; Rotgans, Jerome I.	Team-Based Learning Analytics: An Empirical Case Study	ACADEMIC MEDICINE	2020	10.1097/ACM.0000000000003157	Nanyang Technological University; Nanyang Technological University; Nanyang Technological University; Erasmus University Rotterdam	Netherlands+Singapore	Medicine				Netherlands+Singapore	
Xin, Wei; Zou, Yuxian; Ao, Yong; Cai, Yu; Huang, Zheqian; Li, Miaoling; Xu, Chaochao; Jia, Yu; Yang, Ying; Yang, Yangfan; Lin, Haotian	Evaluation of integrated modular teaching in Chinese ophthalmology trainee courses	BMC MEDICAL EDUCATION	2020	10.1186/s12909-020-02073-w	Sun Yat Sen University; Sun Yat Sen University; Sun Yat Sen University; Sun Yat Sen University; Sun Yat Sen University; Sun Yat Sen University; Sun Yat Sen University; Sun Yat Sen University; Sun Yat Sen University; Sun Yat Sen University; Sun Yat Sen University	China	Medicine			China		

Nayak, Kirtana Raghurama; Punja, Dhiren; Suryavanshi, Chinmay Ajit; Kamath, Asha	Application of Case-Based Readiness Assurance Process as a Model for Case-Based Pedagogy and Collaborative Learning in Physiology	MEDICAL SCIENCE EDUCATOR	2020	10.1007/s40670-020-00967-4	Manipal Academy of Higher Education (MAHE); Manipal Academy of Higher Education (MAHE); Manipal Academy of Higher Education (MAHE); Manipal Academy of Higher Education (MAHE);	India	Medicine		one institution, many authors			
Bindayna, Khalid Mubarak; Qareeballa, Ahmed; Joji, Ronni Mol; Al Mahmeed, Ali; Ezzat, Hicham; Ismaeel, Abdulrahman Yousif; Tabbara, Khaled S.	Student Perception of Microbiology Laboratory Skills Learning Through a Problem-Based Learning Curriculum: Arabian Gulf University Experience	ADVANCES IN MEDICAL EDUCATION AND PRACTICE	2020	10.2147/AMEP.S276221	Arabian Gulf University; Arabian Gulf University; Arabian Gulf University; Arabian Gulf University; Arabian Gulf University; Arabian Gulf University; Arabian Gulf University	Bahrain	Medicine		one institution, many authors			
Pan, San-Qiang; Chan, Lap Ki; Yan, Yu; Yang, Xuesong	Survey of Gross Anatomy Education in China: The Past and the Present	ANATOMICAL SCIENCES EDUCATION	2020	10.1002/ase.1952	Jinan University; Macau University of Science & Technology; Jinan University; Jinan University	China	Medicine			China		
Burgess, Annette; Kalman, Eszter; Haq, Inam; Leaver, Andrew; Roberts, Chris; Bleasel, Jane	Interprofessional team-based learning (TBL): how do students engage?	BMC MEDICAL EDUCATION	2020	10.1186/s12909-020-02024-5	University of Sydney; University of Sydney; University of Sydney; University of Sydney; University of Sydney; University of Sydney	Australia	Interprofessional		one institution, many authors			
Zaja, Roko; Brborovic, Hana; Oroz, Dominik; Vuksinic, Katarina Zahariev; Bubas, Marija; Bozic, Tajana; Milosevic, Milan	Knowledge of and attitudes to occupational and sports medicine among medical students in Zagreb, Croatia	ARHIV ZA HIGIJENU RADA I TOKSIKOLOGIJU-ARCHIVES OF INDUSTRIAL HYGIENE AND TOXICOLOGY	2021	10.2478/aiht-2021-72-3535	University of Zagreb; University of Zagreb; University of Zagreb; Croatian Inst Publ Hlth; Croatian Inst Publ Hlth; Croatian Inst Publ Hlth; Polyclin Dr Zora Profozi	Croatia	Occupational medicine			Croatia		
Park, Hyung-Ran; Park, Eunyoung	Nursing students ' perception of class immersion facilitators in psychiatric nursing: Team-based learning combined with flipped learning	NURSE EDUCATION TODAY	2021	10.1016/j.nedt.2020.104653	Chungbuk National University; Chungnam National University	South Korea	Nursing			South Korea		
Salih, Karim Eldin M. A.; El-Samani, El-Fatih Z.; Bilal, Jalal Ali; Hamid, Emtinan K.; Elfaki, Omer Abdelgadir; Idris, Muawia E. A.; Elsiddig, Hind A.; Salim, Maha M.; Missawi, Hashim; Abass, Mohammed; Elfakey, Walyeldin	Team-Based Learning and Lecture-Based Learning: Comparison of Sudanese Medical Students' Performance	ADVANCES IN MEDICAL EDUCATION AND PRACTICE	2021	10.2147/AMEP.S331296	University of Bahri; University of Bahri; University of Bahri; University of Bisha; University of Bisha; Ahfad Univ Women; Shaqra University; University of Khartoum; Al-Rayan Colleges; Al-Rayan Colleges; University of Bahri; University of Bahri	Sudan+ Saudi Arabia+ Bahrain	Medicine				Bahrain+Saudi Arabia+Sudan	
Ulfa, Yunefit; Igarashi, Yukari; Takahata, Kaori; Shishido, Eri; Horiuchi, Shigeko	Effectiveness of team-based learning on postpartum hemorrhage in midwifery students in Indonesia: A quasi-experimental study	NURSE EDUCATION TODAY	2021	10.1016/j.nedt.2021.105015	St. Luke's International Hospital; St. Luke's International Hospital; St. Luke's International Hospital; St. Luke's International Hospital; St. Luke's International Hospital	Japan	Nursing		one institution, many authors			
Burton, Rob; van de Mortel, Thea; Kain, Victoria	Applying team-based learning in a transnational post registration bachelor of nursing program in Singapore	BMC NURSING	2021	10.1186/s12912-021-00593-4	Griffith University; Griffith University; Griffith University	Australia	Nursing		one institution, many authors			
Khalafalla, Farid G.; Alqaysi, Rafal	Blending team-based learning and game-based learning in pharmacy education	CURRENTS IN PHARMACY TEACHING AND LEARNING	2021	10.1016/j.cptl.2021.06.013	California Health Sciences University (CHSU); California Health Sciences University (CHSU); A.T. Still University of Health Sciences	USA	Pharmacy			USA - multiple states		
Carrasco, Gonzalo A.; Behling, Kathryn C.; Gentile, Matthew; Fischer, Bradford D.; Ferraro, Thomas N.	Effectiveness of a Team-Based Learning exercise in the learning outcomes of a medical pharmacology course: insight from struggling students	NAUNYN-SCHMIEDEBERGS ARCHIVES OF PHARMACOLOGY	2021	10.1007/s00210-021-02093-3	Rowan University; Rowan University; Rowan University; Rowan University; Rowan University	USA	Pharmacy		one institution, many authors			
Allen, John M.; Egelund, Eric F.; Santevecchi, Barbara A.; Venugopalan, Veena; Whalen, Karen; Patel, Priti N.	Impact of supplemental individual verbal defense on confidence, engagement, and performance in a team-based learning therapeutics course	CURRENTS IN PHARMACY TEACHING AND LEARNING	2021	10.1016/j.cptl.2020.09.010	University of Florida; University of Central Florida; University of Florida; University of Florida; University of Florida; University of Florida	USA	Pharmacy			USA - one state		
Yeh, Tzu-Pei; Chang, Shan-Mei; Ho, Ya-Fang; Ma, Wei-Fen	Online Team-Based Learning Teaching Strategy for Developing Caring Competencies in Nursing Students under COVID-19 Pandemic Restrictions	HEALTHCARE	2021	10.3390/healthcare9111510	China Medical University Taiwan; China Medical University Taiwan; China Medical University Taiwan; China Medical University Taiwan	Taiwan	Nursing		one institution, many authors			
Burgess, Annette; Matar, Elie; Roberts, Chris; Haq, Inam; Wynter, Lucy; Singer, Julian; Kalman, Eszter; Bleasel, Jane	Scaffolding medical student knowledge and skills: team-based learning (TBL) and case-based learning (CBL)	BMC MEDICAL EDUCATION	2021	10.1186/s12909-021-02638-3	University of Sydney; University of Sydney; University of Sydney; University of Sydney; Sydney Hlth Profess Educ Res Network; University of Sydney; University of Sydney;	Australia	Medicine			Australia		
Yu, Frank; Wooster, Jessica; Yang, Tianrui	Pharmacy students and faculty perceptions of online team-based learning due to the COVID-19 pandemic	PHARMACY EDUCATION	2021	10.46542/pe.2021.211.121125	University of Texas at Tyler; University of Texas at Tyler; University of Texas at Tyler	USA	Pharmacy		one institution, many authors			

Kim, Mi Eun; Kim, Jin Young	The impact of a team-based learning group readiness assurance test on nursing students? problem solving, learning satisfaction, and team efficacy: A crossover study	NURSE EDUCATION TODAY	2021	10.1016/j.nedt.2021.104819	Jeonju University; Jeonju University	South Korea	Nursing			South Korea		
Albarrak, Ahmed I.; Zakaria, Nasriah; Almulhem, Jwaher; Khan, Samina A.; Karim, Norshahriza Abdul	Modified team-based and blended learning perception: a cohort study among medical students at King Saud University	BMC MEDICAL EDUCATION	2021	10.1186/s12909-021-02639-2	King Saud University; King Saud University; King Saud University; Universiti Malaya; Prince Sultan University	UAE+Malaysia	Medicine				Malaysia+UAE	
Eksteen, M. J.; Reitsma, G. M.; Fourie, E.	Promoting deeper learning in pharmacy education using team-based learning	AFRICAN JOURNAL OF HEALTH PROFESSIONS EDUCATION	2021	10.7196/AJHPE.2021.v13i2.878	University of the Free State; North West University - South Africa; North West University - South Africa	South Africa	Pharmacy			South Africa		
Li, Yun; Sears, Nicholas A.; Murray, Ian V. J.; Yadav, Kamlesh K.	Rethinking Teaching Team-Based Learning: The Challenges and Strategies for Medical Education in a Pandemic	AERA OPEN	2021	10.1177/23328584211067207	Texas A&M University; Texas A&M University; Texas A&M University; Texas A&M University	USA	Medicine		one institution, many authors			
Ulfa, Yunefit; Igarashi, Yukari; Takahata, Kaori; Horiuchi, Shigeko	Effects of team-based learning about postpartum haemorrhage on learning outcomes and experience of midwifery students in Indonesia: A pilot study	NURSING OPEN	2021	10.1002/nop2.623	St. Luke's International Hospital; St. Luke's International Hospital; St. Luke's International Hospital; Shonan Kamakura Univ Med Sci	Japan	Nursing			Japan		
Runyan, Carmelita; Marshall, Caroline; Aronow, Harriet; Vongkavivathanakul, Saisunee; Daniels, Laura; Currey, Judy; Coleman, Bernice	Evaluation of Team-Based Learning to Increase Nurses' Knowledge of the Ventricular Assist Device	JOURNAL OF CONTINUING EDUCATION IN NURSING	2021	10.3928/00220124-20201215-06	Cedars Sinai Medical Center; Cedars Sinai Medical Center; Cedars Sinai Medical Center; Cedars Sinai Medical Center; Deakin University; Cedars Sinai Medical Center	USA	Nursing			USA - multiple states		
Keister, Kathy J.; Farra, Sharon L.; Smith, Sherrill J.; Bottomley, Michael	Development of a Scale to Measure Synergy: A Tool to Evaluate Team-Based Learning in Nursing	NURSING EDUCATION PERSPECTIVES	2021	10.1097/01.NEP.0000000000000783	Northwest State Community Coll; Wright State University Dayton; Wright State University Dayton; Univ Wyoming; Wright State University Dayton	USA	Nursing			USA - multiple states		yes
Silberman, Dave; Carpenter, Rob; Takemoto, Jody K.; Coyne, Leanne	The impact of team-based learning on the critical thinking skills of pharmacy students	CURRENTS IN PHARMACY TEACHING AND LEARNING	2021	10.1016/j.cptl.2020.09.008	University of Texas at Tyler; University of Texas at Tyler; University of Texas at Tyler; University of Texas at Tyler; California Health Sciences University (CHSU); California Health Sciences University (CHSU)	USA	Pharmacy			USA - multiple states		
Martirosov, Amber Lanae; Moser, Lynette R.	Team-based Learning to Promote the Development of Metacognitive Awareness and Monitoring in Pharmacy Students	AMERICAN JOURNAL OF PHARMACEUTICAL EDUCATION	2021	10.5688/ajpe848112	Wayne State University Dayton; Wayne State University Dayton	USA	Pharmacy		one institution, many authors			
Mohammed, Heitham Mutwakil	Team-Based Learning (TBL) Implementation in General Embryology at the Faculty of Medicine - Jazan University, KSA	BAHRAIN MEDICAL BULLETIN	2021		King Khalid University	Saudi Arabia	Medicine	one author				
Liu, Wenyuan; Gao, Xinzhu; Han, Lingfei; Liu, Jing; Feng, Feng	Blended Teaching Practices for Active Learning in Higher Pharmacy Education	INDIAN JOURNAL OF PHARMACEUTICAL EDUCATION AND RESEARCH	2021	10.5530/ijper.55.3.13718012955795	China Pharmaceutical University; China Pharmaceutical University; China Pharmaceutical University; China Pharmaceutical University; China Pharmaceutical University; Jiangsu Food & Pharmaceutical Science College	China	Pharmacy			China		
Zeng, Jinrong; Liu, Liyao; Tong, Xiaoliang; Gao, Lihua; Zhou, Lu; Guo, Aiyuan; Tan, Lina	Application of blended teaching model based on SPOC and TBL in dermatology and venereology	BMC MEDICAL EDUCATION	2021	10.1186/s12909-021-03042-7	Central South University; Central South University; Central South University; Central South University; Central South University; Central South University	China	Medicine		one institution, many authors			
Miao, Julia H.	Adapting Medical Education Initiatives Through Team-Based e-Learning, Telemedicine Objective Structured Clinical Exams, and Student-Led Community Outreach During the COVID-19 Pandemic	JMIR MEDICAL EDUCATION	2021	10.2196/26797	Cornell University; State University of New York (SUNY) Stony Brook	USA	Medicine	one author				
Bertsch, Taylor G.; Denton, Travis T.; Perea, Nicole M.; Ahmed, Ayesha; McKeirnan, Kimberly C.	Drug development and the process of transitioning to team-based learning in a qualitative way	CURRENTS IN PHARMACY TEACHING AND LEARNING	2021	10.1016/j.cptl.2021.01.025	Washington State University; Washington State University; Washington State University; Washington State University; Washington State University	USA	Pharmacy		one institution, many authors			
Lafleur, Alexandre; Rousseau-Gagnon, Mathieu; Cote-Maheux, Marianne; Tremblay-Laroche, Dave; De Cotret, Paul Rene; Caumartin, Yves	Three Weeks of Team-Based Leaning Do Not Overload Undergraduate Students	MEDICAL SCIENCE EDUCATOR	2021	10.1007/s40670-021-01314-x	Laval University; Laval University; Laval University; Laval University; Laval University	Canada	Medicine		one institution, many authors			

Xue, Hui; Yuan, Hua; Li, Guichen; Liu, Jiamei; Zhang, Xiuying	Comparison of team-based learning vs. lecture-based teaching with small group discussion in a master's degree in nursing education course	NURSE EDUCATION TODAY	2021	10.1016/j.nedt.2021.105043	Jilin University; Jilin University; Jilin University; Jilin University; Jilin University	China	Nursing		one institution, many authors			
Ulfa, Yunefit; Igarashi, Yukari; Takahata, Kaori; Shishido, Eri; Horiuchi, Shigeko	A comparison of team-based learning and lecture-based learning on clinical reasoning and classroom engagement: a cluster randomized controlled trial	BMC MEDICAL EDUCATION	2021	10.1186/s12909-021-02881-8	St. Luke's International Hospital; St. Luke's International Hospital; St. Luke's International Hospital; St. Luke's International Hospital; St. Luke's International Hospital	Japan	Medicine		one institution, many authors			
Yaqoob, Muhammad Furqan; Khalid, Zara; Azim, Muhammad Ehab; Ahsan, Sana; Hassan, Muhammad Furqan; Naeem, Aamer	Perceptions regarding team-based learning among undergraduate physical students	JOURNAL OF THE PAKISTAN MEDICAL ASSOCIATION	2021	10.47391/JPMA.1172	Fdn Univ; Fdn Univ; Fdn Univ; Fdn Univ; Fdn Univ; Fdn Univ	Pakistan	Medicine		one institution, many authors			
Vinall, Ruth; Malhotra, Ashim; Puglisi, Jose	Use of Team-Based Learning Pedagogy to Prepare for a Pharmacy School Accreditation Self-Study	PHARMACY	2021	10.3390/pharmacy9030148	Calif Northstate Univ; Calif Northstate Univ; Calif Northstate Univ	USA	Pharmacy		one institution, many authors			
Carrasco, Gonzalo A.; Behling, Kathryn C.; Lopez, Osvaldo	Weekly team-based learning scores and participation are better predictors of successful course performance than case-based learning performance: role of assessment incentive structure	BMC MEDICAL EDUCATION	2021	10.1186/s12909-021-02948-6	Rowan University; Rowan University; Seton Hall Univ	USA	Medicine			USA - one state		
Cremerius, Cassian; Gradl-Dietsch, Gertraud; Beeres, Frank J. P.; Link, Bjoern-Christian; Hitpass, Lea; Nebelung, Sven; Horst, Klemens; Weber, Christian David; Neuerburg, Carl; Eschbach, Daphne; Bliemel, Christopher; Knobe, Matthias	Team-based learning for teaching musculoskeletal ultrasound skills: a prospective randomised trial	EUROPEAN JOURNAL OF TRAUMA AND EMERGENCY SURGERY	2021	10.1007/s00068-019-01298-9	Marienhospital Aachen; University of Duisburg Essen; Lucerne Cantonal Hospital; Lucerne Cantonal Hospital; Lucerne Cantonal Hospital; RWTH Aachen University; RWTH Aachen University; RWTH Aachen University; RWTH Aachen University; University of Munich; University Hospital of Giessen & Marburg	Germany+Switzerland	Medicine				Germany+Switzerland	
Nawabi, Shazia; Bilal, Rabia; Javed, Muhammad Qasim	Team-based learning versus Traditional lecture-based learning: An investigation of students' perceptions and academic achievements	PAKISTAN JOURNAL OF MEDICAL SCIENCES	2021	10.12669/pjms.37.4.4000	Qassim University; Qassim University; Qassim University	Saudi Arabia	Medicine		one institution, many authors			
Lin, I-Chun; Sen-Crowe, Brendon; Pasarin, Anthony; McKenney, Mark; Elkbuli, Adel	Variability in tuition and curriculum among allopathic and osteopathic medical schools in the United States	ANNALS OF MEDICINE AND SURGERY	2021	10.1016/j.amsu.2021.102737	Kendall Reg Med Ctr; Kendall Reg Med Ctr; Kendall Reg Med Ctr; Kendall Reg Med Ctr; Kendall Reg Med Ctr; University of South Florida	USA	Medicine			USA - one state		
Sharma, Ramaswamy; King, Thomas S.; Hanson, Elizabeth R.; Fiebelkorn, Kristin	Medical Histopathology Laboratories: Remote Teaching in Response to COVID-19 Pandemic	ACADEMIC PATHOLOGY	2021	10.1177/2374289521998049	University of Texas Health Science Center at San Antonio; University of Texas Health Science Center at San Antonio; University of Texas Health Science Center at San Antonio; University of Texas Health Science Center at San Antonio	USA	Medicine		one institution, many authors			
Wiersma, Gretchen; Cox, Catherine Wilson; McNelis, Angela M.; Schumann, Mary Jean; Maring, Joyce	Faculty Perceptions in Facilitating Success for Accelerated BSN Student Veterans	NURSING EDUCATION PERSPECTIVES	2021	10.1097/01.NEP.0000000000000673	George Washington University; George Washington University; George Washington University; George Washington University; George Washington University	USA	Nursing		one institution, many authors			
Wai, Abraham K. C.; Lam, Veronica S. F.; Ng, Zoe L. H.; Pang, Michelle T. H.; Tsang, Vivien W. Y.; Lee, Jay J. J.; Wong, Janet Y. H.	Exploring the role of simulation to foster interprofessional teamwork among medical and nursing students: A mixed-method pilot investigation in Hong Kong	JOURNAL OF INTERPROFESSIONAL CARE	2021	10.1080/13561820.2020.1831451	University of Hong Kong; University of Hong Kong; University of Hong Kong; University of Hong Kong; University of Hong Kong; University of Hong Kong; University of Hong Kong;	China	Nursing		one institution, many authors			
Lovato, Chris Y.; Shaw, Graham	Helping Students Reflect on Their Interpersonal Skills: The Team Performance Scale (TPS)	CANADIAN JOURNAL OF PROGRAM EVALUATION	2021	10.3138/cjpe.70376	University of British Columbia; University of British Columbia	Canada	Medicine		one institution, many authors			
Takizawa, Peter A.; Honan, Linda; Brissette, David; Wu, Barry J.; Wilkins, Kirsten M.	Teamwork in the time of COVID-19	FASEB BIOADVANCES	2021	10.1096/fba.2020-00093	Yale University; Yale University; Yale University; Yale University; Yale University	USA	Medicine		one institution, many authors			
Veneri, Diana A.; Mongillo, Elizabeth M. N.	Flop to Flip: Integrating Technology and Team-Based Learning to Improve Student Engagement	INTERNET JOURNAL OF ALLIED HEALTH SCIENCES AND PRACTICE	2021		Sacred Heart University; Quinnipiac University	USA	Medicine			USA - one state		

Eguchi, Hironobu; Sakiyama, Haruhiko; Naruse, Hitoshi; Yoshihara, Daisaku; Fujiwara, Noriko; Suzuki, Keiichiro	Introduction of team-based learning improves understanding of glucose metabolism in biochemistry among undergraduate students	BIOCHEMISTRY AND MOLECULAR BIOLOGY EDUCATION	2021	10.1002/bmb.21485	Hyogo Medical University; Hyogo Medical University; Hyogo Medical University; Hyogo Medical University; Hyogo Medical University	Japan	Medicine		one institution, many authors			
Will, Kristen K.; Essary, Alison	Competency-Based Interprofessional Continuing Education Focusing on Systems Thinking and Health Care Delivery for Health Care Professionals	JOURNAL OF CONTINUING EDUCATION IN THE HEALTH PROFESSIONS	2021	10.1097/CEH.0000000000000330	Arizona State University-Tempe; Arizona State University-Scottsdale	USA	Interprofessional			USA - one state		
Tahir, Mohamed Elnajid; Elhuda, Daffalla Mohammed Elamean Alam; Gasmalla, Hosam Eldeen Elsadig	The Perception of Physiologists Toward Implementing Team-based Learning in Sudan: A national-scale Cross-sectional Study	SUDAN JOURNAL OF MEDICAL SCIENCES	2021	10.18502/sjms.v16i1.8941	Sudan Int Univ; Univ Med Sci & Technol UMST; University of Khartoum; Al Neelain Univ; Sudan Int Univ;	Sudan	Medicine			Sudan		
Chow, Ava K.; Sharmin, Nazlee	Developing an Interactive Computer Program for Integrated Dental Education	HEALTHCARE INFORMATICS RESEARCH	2021	10.4258/hir.2021.27.4.335	University of Alberta; University of Alberta	Canada	Medicine		one institution, many authors			
Parappilly, Maria; Woodman, Richard John; Randhawa, Sharmil	Feasibility and Effectiveness of Different Models of Team-Based Learning Approaches in STEMM-Based Disciplines	RESEARCH IN SCIENCE EDUCATION	2021	10.1007/s11165-019-09888-8	Flinders University South Australia; Flinders University South Australia; Flinders University South Australia	Australia	Medicine		one institution, many authors			
Ding, Chun; Wang, Qin; Zou, Jingling; Zhu, Kewei	Implementation of flipped classroom combined with case- and team-based learning in residency training	ADVANCES IN PHYSIOLOGY EDUCATION	2021	10.1152/advan.00022.2020	Central South University; Central South University; Central South University; Central South University	China	Medicine		one institution, many authors			
Anderson, Apryl N.; Patterson, Julie A.; Donohoe, Krista; Matulewicz, Abigale T.; Frankart, Laura M.; Peron, Emily P.; Caldas, Lauren M.	Lessons learned: Assessing team creation by personality inventories in pharmacy students	CURRENTS IN PHARMACY TEACHING AND LEARNING	2021	10.1016/j.cptl.2021.09.014	Virginia Commonwealth University; Virginia Commonwealth University; Virginia Commonwealth University; Virginia Commonwealth University; Virginia Commonwealth University; Virginia Commonwealth University; Virginia Commonwealth University	USA	Pharmacy		one institution, many authors			
Bhattacharya, Shelley B.; Jernigan, Stephen; Hyatt, Myra; Sabata, Dory; Johnston, Shane; Burkhardt, Crystal	Preparing a healthcare workforce for geriatrics care: an Interprofessional team based learning program	BMC GERIATRICS	2021	10.1186/s12877-021-02456-8	University of Kansas; University of Kansas; University of Kansas; University of Kansas; University of Kansas; University of Kansas	USA	Interprofessional		one institution, many authors			
Nawabi, Shazia; Javed, Muhammad Qasim; Bilal, Rabia	Problem-based Learning and Team-based Learning as a Novel Package Approach	JCPSP-JOURNAL OF THE COLLEGE OF PHYSICIANS AND SURGEONS PAKISTAN	2021	10.29271/jcpsp.2021.06.710	Qassim University; Qassim University; Qassim University	Saudi Arabia	Medicine		one institution, many authors			
Singh, Nina; Phoon, Colin K. L.	Not yet a dinosaur: the chalk talk	ADVANCES IN PHYSIOLOGY EDUCATION	2021	10.1152/advan.00126.2020	NYU Langone Medical Center; New York University	USA	Medicine			USA - one state		
Stout, Rebecca C.; Roberts, Sophie; Maxwell-Scott, Hector; Gothard, Philip	Necessity is the mother of invention: how the COVID-19 pandemic could change medical student placements for the better	POSTGRADUATE MEDICAL JOURNAL	2021	10.1136/postgradmedj-2021-139728	University College London Hospitals NHS Foundation Trust; University College London Hospitals NHS Foundation Trust; University College London Hospitals NHS Foundation Trust; University College London Hospitals NHS Foundation Trust	UK	Medicine			England		
Bork, Felix; Lehner, Alexander; Eck, Ulrich; Navab, Nassir; Waschke, Jens; Kugelmann, Daniela	The Effectiveness of Collaborative Augmented Reality in Gross Anatomy Teaching: A Quantitative and Qualitative Pilot Study	ANATOMICAL SCIENCES EDUCATION	2021	10.1002/ase.2016	Technical University of Munich; Technical University of Munich; Technical University of Munich; University of Munich; University of Munich	Germany	Medicine			Germany		
Omer, Ahmad AbdulAzeem Abdullah	The Early Milestones of Team-based Learning: The Key is Sustained Practice	SUDAN JOURNAL OF MEDICAL SCIENCES	2021	10.18502/sjms.v16i3.9704	Prince Sattam Bin Abdu Aziz Univ	Saudi Arabia	Medicine	one author				
Chen, Jui-O; Chang, Shu-Chen; Lin, Chiu-Chu	The development and pilot testing of an ACP simulation-based communication-training program: Feasibility and acceptability	PLOS ONE	2021	10.1371/journal.pone.0254982	Kaohsiung Medical University; Changhua Christian Hospital; Da Yeh University; Kaohsiung Medical University; Kaohsiung Medical University; Kaohsiung Medical University; Kaohsiung Medical University Hospital	Taiwan	Medicine			Taiwan		
Chaturvedi, Saurabh; Elmahdi, Asim Elsir; Abdelmonem, Adel M.; Haralur, Satheesh B.; Alqahtani, Nasser M.; Suleman, Ghazala; Sharif, Rania A.; Gurumurthy, Vishwanath; Alfarsi, Mohammed A.	Predoctoral dental implant education techniques-students' perception and attitude	JOURNAL OF DENTAL EDUCATION	2021	10.1002/jdd.12453	King Khalid University; King Khalid University; King Khalid University; King Khalid University; King Khalid University; King Khalid University; King Khalid University; King Khalid University; King Khalid University	Saudi Arabia	dentistry		one institution, many authors			
Lee, Sun Hee; Park, Hyun Jung	The effects of team-based learning on performance with a focus on high-risk a quasi-experimental study	KOREAN JOURNAL OF WOMEN HEALTH NURSING	2021	10.4069/kjwhn.2021.11.16	Gimcheon Univ; Pyeongtaek University	South Korea	Nursing			South Korea		

Langer, Arielle L.; Binder, Adam F.; Scigliano, Eileen	Long-term Outcomes of team-based learning	CLINICAL TEACHER	2021	10.1111/tct.13332	Brigham & Womens Hosp; Thomas Jefferson Univ; Icahn School of Medicine at Mount Sinai	USA	Medicine			USA - multiple states		
Arain, Shoukat Ali; Alhadid, Daeya Ahmad; Rasheed, Shahzad; Alrefaai, Maram Mansour; Alsibai, Tarek M. Ahyaf; Meo, Sultan Ayoub	Perceived effectiveness of learning methods among preclinical medical students- role of personality and changes over time	PAKISTAN JOURNAL OF MEDICAL SCIENCES	2021	10.12669/pjms.37.7.4355	Alfaisal University; Alfaisal University; Alfaisal University; Alfaisal University; Imam Mohammad Ibn Saud Islamic University (IMSIU); King Saud University	Saudi Arabia	Medicine			Saudi Arabia		
Alamoudi, Aliaa Amr; Al Shawwa, Lana Adey; Gad, Hoda; Tekian, Ara	Team-based learning versus traditional didactic lectures in teaching clinical biochemistry at King Abdulaziz University; learning outcomes and student satisfaction	BIOCHEMISTRY AND MOLECULAR BIOLOGY EDUCATION	2021	10.1002/bmb.21501	King Abdulaziz University; King Abdulaziz University; King Abdulaziz University; Alexandria University; University of Illinois Chicago	USA+Saudi Arabia	Medicine				Saudi Arabia+USA	
Papanagnou, Dimitrios; Ankam, Nethra; Ebbott, David; Ziring, Deborah	Towards a medical school curriculum for uncertainty in clinical practice	MEDICAL EDUCATION ONLINE	2021	10.1080/10872981.2021.1972762	Jefferson University; Jefferson University; Jefferson University; Jefferson University	USA	Medicine		one institution, many authors			
Choi, JiYeon; Lee, Seung Eun; Bae, Juyeon; Kang, Suyeon; Choi, Seongmi; Tate, Judith A.; Yang, You Lee	Undergraduate nursing students ' experience of learning respiratory system assessment using flipped classroom: A mixed methods study	NURSE EDUCATION TODAY	2021	10.1016/j.nedt.2020.104664	Yonsei University; Yonsei University; Yonsei University; Yonsei University; Ohio State University; Ohio State University; Eulji University	USA+South Korea	Nursing				South Korea+USA	
Khanna, Aakanksha; Ravindran, Adharsh; Ewing, Brandon; Zinnerstrom, Karen; Grabowski, Connor; Mishra, Archana; Makdissi, Regina	Escape MD: Using an Escape Room as a Gamified Educational and Skill-Building Teaching Tool for Internal Medicine Residents	CUREUS JOURNAL OF MEDICAL SCIENCE	2021	10.7759/cureus.18314	Jacobs Sch Med & Biomed Sc; Jacobs Sch Med & Biomed Sc; Jacobs Sch Med & Biomed Sc; Jacobs Sch Med & Biomed Sc; Jacobs Sch Med & Biomed Sc; Jacobs Sch Med & Biomed Sc; Jacobs Sch Med & Biomed Sc	USA	Medicine			USA - one state		
Hastuti, Agustina A. M. B.; Noviana, Eka; Siswanto, Soni; Indrasetiawan, Puguh; Endarti, Dwi; Kristina, Susi Ari; Hertiani, Triana	Implementing online team-based learning in an interuniversity setting: A case study of a traditional medicine course	PHARMACY EDUCATION	2022	10.46542/pe.2022.221.558568	Gadjah Mada University; Gadjah Mada University; Gadjah Mada University; Gadjah Mada University; Gadjah Mada University; Gadjah Mada University;	Indonesia	Pharmacy		one institution, many authors			
Basheer, Haneen A.; Isreb, Mohammad; Batarseh, Yazan S.; Tweddell, Simon	Team-Based Learning Approach for the Delivery of Over-the-counter Module in the Faculty of Pharmacy in Jordan	INDIAN JOURNAL OF PHARMACEUTICAL EDUCATION AND RESEARCH	2022	10.5530/ijper.56.2s.85	Zarqa University; University of Bradford; University of Bradford; Petra University	UK+Jordan	Pharmacy				UK+Jordan	
Daou, Dayane; Chakhtoura, Marlene; El-Yazbi, Ahmed; Mukherji, Deborah; Sbaity, Eman; Refaat, Marwan M.; Nabulsi, Mona	Teaching critical appraisal to large classes of undergraduate medical students using team-based learning versus group discussions: a randomized controlled trial	BMC MEDICAL EDUCATION	2022	10.1186/s12909-022-03145-9	American University of Beirut; American University of Beirut; American University of Beirut; American University of Beirut; American University of Beirut; American University of Beirut; American University of Beirut	Lebanon	Medicine		one institution, many authors			
Rao, Padma G. M.; Rabbani, Syed Arman	Team Based Learning Experience from a College of Pharmaceutical Sciences in United Arab Emirates	INDIAN JOURNAL OF PHARMACEUTICAL EDUCATION AND RESEARCH	2022	10.5530/ijper.56.4.177	RAK Med & Hlth Sci Univ; RAK Med & Hlth Sci Univ	UAE	Pharmacy		one institution, many authors			
Park, Hyung-Ran; Park, Eunyoung	Team-Based Learning Experiences of Nursing Students in a Health Assessment Subject: A Qualitative Study	HEALTHCARE	2022	10.3390/healthcare10050817	Chungbuk National University; Chungnam National University	South Korea	Nursing			South Korea		
Shen, Jing; Qi, Hongyan; Chen, Yingying; Mei, Ruhuan; Sun, Cencen; Wang, Zhengyang	Incorporating modified team-based learning into a flipped basic medical laboratory course: impact on student performance and perceptions	BMC MEDICAL EDUCATION	2022	10.1186/s12909-022-03676-1	Zhejiang University; Zhejiang University; Zhejiang University; Zhejiang University; Zhejiang University; Zhejiang University	China	Medicine		one institution, many authors			
Chen, Dong; Yue, Haitang; Liu, Shengbo; Meng, Lang; Yin, Wei	The introduction of team-based learning into the clinical pharmacology section of the endodontics clinical course	CLINICAL AND EXPERIMENTAL PHARMACOLOGY AND PHYSIOLOGY	2022	10.1111/1440-1681.13684	Wuhan University; Wuhan University; Wuhan University; Wuhan University; Wuhan University	China	Pharmacy		one institution, many authors			
Ho, Jacqueline Mei-Chi; Wong, Arnold Yu-Lok; Schoeb, Veronika; Chan, Alex Siu-Wing; Tang, Patrick Ming-Kuen; Wong, Frances Kam-Yuet	Interprofessional Team-Based Learning: A Qualitative Study on the Experiences of Nursing and Physiotherapy Students	FRONTIERS IN PUBLIC HEALTH	2022	10.3389/fpubh.2021.706346	Hong Kong Polytechnic University; Hong Kong Polytechnic University; Hong Kong Polytechnic University; Hong Kong Polytechnic University; University of Applied Sciences & Arts Western Switzerland; Chinese University of Hong Kong	China+Switzerland	Nursing				China+Switzerland	
Costa, Sarah Teixeira; Manzi, Flavio Ricardo; Lima, Izabella Lucas de Abreu; Pinto, Yasmin Dias de Almeida; Miranda, Diogo de Azevedo	TEAM BASED LEARNING: APPLICATION OF ACTIVE TEACHING METHODOLOGY AS EVALUATION IN THE PRE-CLINICAL MOMENT IN THE DENTISTRY COURSE	REVISTA IBERO-AMERICANA DE ESTUDOS EM EDUCACAO	2022	10.21723/riaee.v17i4.17154	Almeida Neves Univ UNIPTAN; Universidade Estadual de Campinas; Universidade Estadual de Campinas; Pontif Catholic Univ PUC; Pontif Catholic Univ PUC; Universidade Federal de Minas Gerais; Universidade Estadual de Campinas	Brazil	Dentistry			Brazil		

Woodcock, Jade; Henderson, Charles; Sheakley, Maria	To What Extent Do Faculty and Students Believe that Team-Based Learning Supports Important Goals of Undergraduate Medical Education?	MEDICAL SCIENCE EDUCATOR	2022	10.1007/s40670-022-01632-8	Western Michigan University; Western Michigan University; Western Michigan University	USA	Medicine		one institution, many authors			
Carrasco, Gonzalo A.; Gentile, Matthew; Salvatore, Michelle L.; Lopez, Osvaldo J.; Behling, Kathryn C.	Implementation of team-based learning (TBL) in a second year medical school course: does prior experience with TBL improve the impact of this pedagogy?	BMC MEDICAL EDUCATION	2022	10.1186/s12909-022-03363-1	Rowan University; Rowan University; Rowan University; Cooper University Hospital; Cooper University Hospital; Seton Hall Univ	USA	Medicine			USA - one state		
Babenko, Oksana; Ding, Mao; Lee, Ann S.	In-Person or Online? The Effect of Delivery Mode on Team-Based Learning of Clinical Reasoning in a Family Medicine Clerkship	MEDICAL SCIENCES	2022	10.3390/medsci10030041	University of Alberta; University of Alberta; University of Alberta	Canada	Medicine		one institution, many authors			
Carpenter, Rob E.; Silberman, Dave; Takemoto, Jody K.	The Student Engagement Effect of Team-Based Learning on Student Pharmacists	AMERICAN JOURNAL OF PHARMACEUTICAL EDUCATION	2022		University of Texas at Tyler; Boston University; University of Texas at Tyle	USA	Pharmacy		one institution, many authors			
Cunningham, Christopher J.; Giusto, Joseph; Reiss, Rachel; Garba, Deen L.; Lucke, Austin; Eltilib, Mohamed; Hastie, Eric	A team-based learning model using clinical vignettes in an advanced undergraduate pre-health professional physiology course facilitated by medical students	ADVANCES IN PHYSIOLOGY EDUCATION	2022	10.1152/advan.00174.2021	University of North Carolina Chapel Hill; University of North Carolina Chapel Hill; University of North Carolina Chapel Hill; University of North Carolina Chapel Hill; University of North Carolina Chapel Hill; University of North Carolina Chapel Hill; University of North Carolina Chapel Hill	USA	Medicine		one institution, many authors			yes
Zeb, Muhammad Asif; Mahboob, Usman; Shaheen, Neelofar	Effect of team-based learning on critical thinking: A quasi-experimental study	PAKISTAN JOURNAL OF MEDICAL SCIENCES	2022	10.12669/pjms.38.8.6146	Khyber Medical University; Khyber Medical University; Peshawar Med Coll; Riphah Int Univ	Pakistan	Medicine			Pakistan		
Eudaley, Sarah T.; Farland, Michelle Z.; Melton, Tyler; Brooks, Shelby P.; Heidel, R. Eric; Franks, Andrea S.	Student Performance With Graded vs. Ungraded Readiness Assurance Tests in a Team- Based Learning Elective	AMERICAN JOURNAL OF PHARMACEUTICAL EDUCATION	2022		University of Tennessee; University of Tennessee; University of Tennessee; University of Florida; University of Louisiana Monroe; University of Tennessee; University of Tennessee	USA	Pharmacy			USA - multiple states		
Burgess, Annette W.; Luscombe, Georgina M.; Ramsey-Stewart, George	An intensive anatomy by whole-body dissection elective: A longitudinal study	CLINICAL ANATOMY	2022	10.1002/ca.23861	University of Sydney; University of Sydney; University of Sydney	Australia	Medicine		one institution, many authors			
Courtney, Jennifer; Kreys, Eugene; Luu, Bryan; Kreys, Tiffany; Vinall, Ruth; Quang, Vy; Titus-Lay, Erika	Effectiveness of an Advanced Naloxone Training, Simulation, and Assessment of Second-Year Pharmacy Students	PHARMACY	2022	10.3390/pharmacy10060153	Calif Northstate Univ; Calif Northstate Univ; Calif Northstate Univ; Calif Northstate Univ; Calif Northstate Univ; Calif Northstate Univ; Calif Northstate Univ	USA	Pharmacy		one institution, many authors			
Carpenter, Rob Edwin; Coyne, Leanne; Silberman, Dave; Takemoto, Jody Kyoto	Enhanced numeracy skills following team-based learning in United States pharmacy students: a longitudinal cohort study	JOURNAL OF EDUCATIONAL EVALUATION FOR HEALTH PROFESSIONS	2022	10.3352/jeehp.2022.19.29	University of Texas at Tyler; West Coast Univ; West Coast Univ; Boston University	USA	Pharmacy			USA - multiple states		
Gong, Jie; Du, Junfeng; Hao, Jinjin; Li, Lei	Effects of bedside team-based learning on pediatric clinical practice in Chinese medical students	BMC MEDICAL EDUCATION	2022	10.1186/s12909-022-03328-4	Huazhong University of Science & Technology; Huazhong University of Science & Technology; Huazhong University of Science & Technology; Jingshan Peoples Hosp	China	Medicine			China		
Ahmed, Masood; Athar, Saima; Zainab, Saima; Akbani, Shaheena; Hasan, Batool; Hameed, Uzma	Does team-based learning affect test scores of the basic medical sciences students in a modular curriculum?	INTERNATIONAL JOURNAL OF HEALTH SCIENCES-IJHS	2022		Fazaia Ruth Pfau Med Coll; Liaquat Natl Hosp & Med Coll; Liaquat Natl Hosp & Med Coll; Liaquat Natl Hosp & Med Coll; Liaquat Natl Hosp & Med Coll; Dow Univ Hlth Sci	Pakistan	Medicine			Pakistan		
Yang, Shang-Yu; Liu, Cheng; Hsieh, Pei-Lun	Effects of Team-Based Learning on Students' Teamwork, Learning Attitude, and Health Care Competence for Older People in the Community to Achieve SDG-3	INTERNATIONAL JOURNAL OF ENVIRONMENTAL RESEARCH AND PUBLIC HEALTH	2022	10.3390/ijerph19116632	Asia University Taiwan; Asia University Taiwan; National Taichung University of Science & Technology	Taiwan	Medicine			Taiwan		
Spencer, Sandra P.; Lauden, Stephanie; Wilson, Sheria; Philip, Andrew; Kasick, Rena; Mahan, John D.; Fernandes, Ashley K.	Meeting the challenge of teaching bioethics: a successful residency curricula utilizing Team-Based Learning	ANNALS OF MEDICINE	2022	10.1080/07853890.2021.2013523	Nationwide Childrens Hospital; Ohio State University; Ohio State University; Ohio State University; Ohio State University; Ohio State University; Nationwide Childrens Hospital; Nationwide Childrens Hospital; Nationwide Childrens Hospital; Ohio State Coll Med; Nationwide Childrens Hospital; Ohio State University;	USA	Medicine			USA - multiple states		
Heginbotham, Lori; Baugh, Gina; Lefeber, Timothy; Friehling, Linda; Barnhart, Christy; Miller, Lee Ann; Moore, Lucas; Cottrell, Lesley	A parent-led, patient-centered medical home model instruction for interprofessional undergraduate and graduate learning opportunities	MEDICAL EDUCATION ONLINE	2022	10.1080/10872981.2021.2012105	West Virginia University; West Virginia University; West Virginia University; West Virginia University; West Virginia University; West Virginia University; West Virginia University; West Virginia University	USA	Medicine		one institution, many authors			
Prabha, M. Lakshmi; Rani, A. Geetha; Maheswari, Y. Nisha; Ramya, J. Ezhil	Impact and Perception of Virtual Team-based Learning in Comparison to Online Lectures in Pharmacology- A Randomised	JOURNAL OF CLINICAL AND DIAGNOSTIC RESEARCH	2022	10.7860/JCDR/2022/58726.17372	Tirunelveli Med Coll; Tirunelveli Med Coll; Tirunelveli Med Coll; Tirunelveli Med Coll	India	Pharmacy		one institution, many authors			

	Crossover Interventional Study											
Subedi, Nuwadatta; Hirachan, Neelu; Paudel, Sabita; Shrestha, Bijayata; Pradhan, Anju; Subedee, Anish; Li, Xiaodan	The effectiveness of online team-based learning in introduction to medical ethics education for medical students at a medical college of Nepal: a pilot study	BMC MEDICAL EDUCATION	2022	10.1186/s12909-022-03813-w	Gandaki Med Coll Teaching Hosp & Res Ctr; Gandaki Med Coll Teaching Hosp & Res Ctr; Gandaki Med Coll Teaching Hosp & Res Ctr; Gandaki Med Coll Teaching Hosp & Res Ctr; BP Koirala Inst Hlth Sci; Southern Medical University - China	China+Nepal	Medicine				China+Nepal	
Zagury-Orly, Ivry; Kamin, Daniel S.; Krupat, Edward; Charlin, Bernard; Fernandez, Nicolas; Fischer, Krisztina	The Student-Generated Reasoning Tool (SGRT): Linking medical knowledge and clinical reasoning in preclinical education	MEDICAL TEACHER	2022	10.1080/0142159X.2021.1967904	Universite de Montreal; Harvard University; Harvard University; Boston Children's Hospital; Boston Children's Hospital; Harvard University; Universite de Montreal; Universite de Montreal; Harvard University	USA+Canada	Medicine				Canada+USA	
Lee, Irene Cheng Jie; Wong, Peiyan; Goh, Suzanne Pei Lin; Cook, Sandy	A Synchronous Hybrid Team-Based Learning Class: Why and How to Do It?	MEDICAL SCIENCE EDUCATOR	2022	10.1007/s40670-022-01538-5	National University of Singapore; National University of Singapore; National University of Singapore; National University of Singapore; National University of Singapore; KK Women's & Children's Hospital	Singapore	Medicine			Singapore		
Rajeswarie, S.; Praveen, K.; Sangam, Muralidhar Reddy; Vinay, G.; Bokan, Raju R.; Deka, Roonmoni; Kaur, Amandeep	Comparison of Team-Based Learning Over Conventional Didactic Lecture Among Second-Year MBBS Students	CUREUS JOURNAL OF MEDICAL SCIENCE	2022	10.7759/cureus.21792	Al Azhar Med Coll & Super Specialty Hosp; All India Inst Med Sci; All India Inst Med Sci; All India Inst Med Sci; All India Inst Med Sci; All India Inst Med Sci; All India Inst Med Sci	India	Medicine			India		
Khamphaya, Tanaporn; Pouyfung, Phisit; Yimthiang, Supabhorn	Enhancing Toxicology Achievement by the VARK and the GRSLSS-mixed Models in Team-Based Learning	FRONTIERS IN PUBLIC HEALTH	2022	10.3389/fpubh.2021.732550	Walailak University; Walailak University; Walailak University	Thailand	Medicine		one institution, many authors			
Oliver, Michael; Fernberg, Taylor; Lyons, Paul; Elango, Sambandam; Green, Gordon J.; Talib, Zohray M.	Addressing health disparities in hispanic communities through an innovative team-based medical spanish program at the medical school level - a single-institution study	BMC MEDICAL EDUCATION	2022	10.1186/s12909-022-03151-x	Calif Univ Sci & Med; Calif Univ Sci & Med; Calif Univ Sci & Med; Calif Univ Sci & Med; Calif Univ Sci & Med; Calif Univ Sci & Med	USA	Medicine		one institution, many authors			
Miselis, Heather H.; Zawacki, Stacey; White, Susan; Yinusa-Nyahkoon, Leanne; Mostow, Carol; Furlong, Janice; Mott, Katherine K.; Kumar, Anika; Winter, Michael R.; Berklein, Flora; Jack, Brian	Interprofessional education in the clinical learning environment: a mixed-methods evaluation of a longitudinal experience in the primary care setting	JOURNAL OF INTERPROFESSIONAL CARE	2022	10.1080/13561820.2022.2025768	Boston University; Boston University; Boston University; Boston University; Boston University; Boston University; Boston University; Boston University; Boston University; Boston University; Boston Medical Center	USA	Interprofessional			USA - one state		
Li, Wanmei; Ouyang, Yani; Xu, Jun; Zhang, Pengfei	Implementation of the Student-Centered Team-Based LearningTeaching Method in a Medicinal Chemistry Curriculum	JOURNAL OF CHEMICAL EDUCATION	2022	10.1021/acs.jchemed.1c00978	Hangzhou Normal University; Hangzhou Normal University; Hangzhou Normal University; Hangzhou Normal University	China	Medicine		one institution, many authors			
Aoe, Mai; Nagata, Misa; Ueda, Masahiro; Kushihata, Taro; Kurio, Wasako; Sone, Tomomichi; Yasuhara, Tomohisa	Effect of prior knowledge and peer evaluation ratings on final exam performance in a team-based learning chemistry course	CURRENTS IN PHARMACY TEACHING AND LEARNING	2022	10.1016/j.cptl.2022.07.016	Osaka Ohtani Univ; Wakayama Medical University; Wakayama Medical University; Setsunan University; Setsunan University; Setsunan University; Setsunan University	Japan	Pharmacy			Japan		
Bell, Edward C.	Individual Vs. Team Based Readiness Assurance Testing in Pharmacy Calculations	INTERNATIONAL JOURNAL OF INSTRUCTION	2022	10.29333/iji.2022.15142a	Texas Southern University	USA	Pharmacy	one author				
Malhotra, Ashim; Oh, Song; Jin, Zhuqiu; Feng, Xiaodong	Closing the Integration Gap: A Pilot for Incorporating Foundational Sciences, DEI-Decision Making, Empathy, and Communication for Congestive Heart Failure and Arrhythmia Management by Pharmacy Students	PHARMACY	2022	10.3390/pharmacy10040077	Calif Northstate Univ; Calif Northstate Univ; Calif Northstate Univ; Saint Joseph's University	USA	Pharmacy		one institution, many authors			
Al-Neklawy, Ahmed Farid; Ismail, Amira Salem Alsagheer	Online anatomy team-based learning using blackboard collaborate platform during COVID-19 pandemic	CLINICAL ANATOMY	2022	10.1002/ca.23797	Ain Shams University; Fakeeh College for Medical Sciences; Suez Canal University; Fakeeh College for Medical Sciences	Saudi Arabia+Egypt	Medicine				Egypt+Saudi Arabia	
Roossien, Linda; Boerboom, Tobias B. B.; Spaai, Gerard W. G.; de Vos, Rien	Team-based learning (TBL): Each phase matters! An empirical study to explore the importance of each phase of TBL	MEDICAL TEACHER	2022	10.1080/0142159X.2022.2064736	University of Amsterdam; University of Amsterdam; University of Amsterdam; University of Amsterdam	Netherlands	Medicine		one institution, many authors			
Hassan, Sherif S.; Nausheen, Fauzia; Scali, Frank; Mohsin, Hina; Thomann, Charity	A constructivist approach to teach neuroanatomy lab: Students' perceptions of an active learning environment	SCOTTISH MEDICAL JOURNAL	2022	10.1177/00369330221107101	Cairo University; Cairo University; Cairo University; Cairo University; Cairo University	Egypt	Medicine			Egypt		

Govindarajan, Sumitra; Rajaragupathy, Sujatha	Online team based learning in teaching Biochemistry for first year MBBS students during COVID-19 pandemic	BIOCHEMISTRY AND MOLECULAR BIOLOGY EDUCATION	2022	10.1002/bmb.21598	PSG Inst Med Sci & Res; PSG Inst Med Sci & Res	India	Medicine		one institution, many authors			
Tyebally, Arif; Dong, Chaoyan	An eLearning program to prepare residents for a rotation in pediatric emergency medicine: A qualitative study	HONG KONG JOURNAL OF EMERGENCY MEDICINE	2022	10.1177/10249079211044911	KK Women's & Children's Hospital; Sengkang Gen Hosp	Singapore	Medicine			Singapore		
Wilson, Jennifer A.; Waghel, Rashi C.; Dinkins, Melissa M.	Impact of two methods for assigning groups in a team-based learning self-care course	CURRENTS IN PHARMACY TEACHING AND LEARNING	2022	10.1016/j.cptl.2022.02.005	Wingate University; Wingate University; Wingate University	USA	Pharmacy		one institution, many authors			
Pitts, Merideth (Hoagland); Smith, Nathanael J.; Bates, Jeffrey A.; Stevenson, Rachel; Fohner, Amy M.; Moser, Haylee M.; Pahl, Brenda; Chen, Aleda M. H.	Development and perceptions of an academic success tool for pharmacy students	RESEARCH IN SOCIAL & ADMINISTRATIVE PHARMACY	2022	10.1016/j.sapharm.2021.08.012	Cedarville University; Cedarville University; Cedarville University; Cedarville University; Cedarville University; Cedarville University; Cedarville University; Cedarville University	USA	Pharmacy		one institution, many authors			
Saadaldin, Selma A.; Eldwakhly, Elzahraa; Alaziz, Sundus Naji; Aldegheishem, Alhanoof; El Sawy, Amal M.; Fahmy, Maha M.; Alsamady, Sahar M.; Sawan, Nozha M.; Soliman, Mai	Team-Based Learning in Prosthodontics Courses: Students' Satisfaction	INTERNATIONAL JOURNAL OF DENTISTRY	2022	10.1155/2022/4546381	Western University (University of Western Ontario); Princess Nourah bint Abdulrahman University; Princess Nourah bint Abdulrahman University; Princess Nourah bint Abdulrahman University; Princess Nourah bint Abdulrahman University; Princess Nourah bint Abdulrahman University; Princess Nourah bint Abdulrahman University; Princess Nourah bint Abdulrahman University; Princess Nourah bint Abdulrahman University	Canada +Saudi Arabia	dentistry				Canada+Saudi Arabia	
Anas, Shafeena; Kyrou, Ioannis; Rand-Weaver, Mariann; Karteris, Emmanouil	The effect of online and in-person team-based learning (TBL) on undergraduate endocrinology teaching during COVID-19 pandemic	BMC MEDICAL EDUCATION	2022	10.1186/s12909-022-03173-5	Brunel University; Brunel University; Brunel University; Univ Hosp Coventry & Warwickshire NHS Trust; Coventry University; Aston University; University of Warwick; Harefield Hospital	UK	Medicine			England		
Wu, Wenyi; Pu, Li; Zhang, Endong; Xiong, Siqi; Zhou, Xiaolai; Xia, Xiaobo; Wen, Dan	Application of team-based learning to ophthalmology in China	FRONTIERS IN PUBLIC HEALTH	2022	10.3389/fpubh.2022.922325	Central South University; Central South University; Central South University;Hunan Key Lab Ophthalmol; Hunan Key Lab Ophthalmol; Hunan Key Lab Ophthalmol; Hunan Key Lab Ophthalmol; Hunan Key Lab Ophthalmol; Sun Yat Sen University	China	Medicine			China		
James, Mariel; Baptista, Ana Madeira Teixeira; Barnabas, Deepak; Sadza, Agata; Smith, Susan; Usmani, Omar; John, Chris	Collaborative case-based learning with programmatic team-based assessment: a novel methodology for developing advanced skills in early-years medical students	BMC MEDICAL EDUCATION	2022	10.1186/s12909-022-03111-5	Imperial College London; Imperial College London; Imperial College London; Imperial College London; Imperial College London; Imperial College London; Imperial College London	UK	Medicine		one institution, many authors			
Sharif, Asmaa F.; Kasemy, Zeinab A.; Alquraishi, Nada A.; Alshaikh, Kawther N.; Alfaraj, Anwar H.; Alibrahim, Eman A.; Alfarej, Zainb M.; Alawami, Hawra M.; Elkhamisy, Fatma Alzahraa A.	Inserting an Erroneous Element in the Answer Key of Grouped Team Readiness Assurance Test in Team-Based Learning: A Randomized Controlled Trial in Clinical Toxicology	ADVANCES IN MEDICAL EDUCATION AND PRACTICE	2022	10.2147/AMEP.S374299	Tanta University; Menofia University; Dar Al Uloom University; Dar Al Uloom Univ; Dar Al Uloom Univ; Dar Al Uloom Univ; Dar Al Uloom Univ; King Salman International University; Helwan University; Egyptian Knowledge Bank (EKB); Tanta University	Saudi Arabia+ Egypt	Medicine				Egypt+Saudi Arabia	
Huilaja, Laura; Bur, Eeva; Jokelainen, Jari; Sinikunnpu, Suvi-Paivikki; Kulmala, Petri	The Effectiveness and Student Perceptions of Peer-Conducted Team-Based Learning Compared to Faculty-Led Teaching in Undergraduate Teaching	ADVANCES IN MEDICAL EDUCATION AND PRACTICE	2022	10.2147/AMEP.S358360	University of Oulu; University of Oulu; University of Oulu; University of Oulu; University of Oulu	Findland	Medicine		one institution, many authors			
Seet, Hong An Andrew; Tan, Emmanuel; Rajalingam, Preman	Effect of Seating Arrangement on Class Engagement in Team-based Learning: a Quasi-Experimental Study	MEDICAL SCIENCE EDUCATOR	2022	10.1007/s40670-021-01469-7	Nanyang Technological University; Nanyang Technological University; Nanyang Technological University	Singapore	Medicine		one institution, many authors			
Dharamsi, Miraal S.; Bastian, D. Anthony; Balsiger, Heather A.; Cramer, Joel T.; Belmares, Ricardo	Efficacy of Video-Based Forearm Anatomy Model Instruction for a Virtual Education Environment	JOURNAL OF MEDICAL EDUCATION AND CURRICULAR DEVELOPMENT	2022	10.1177/23821205211063287	Texas Tech University; Texas Tech University; Texas Tech University; HCA Las Palmas Del Sol Healthcare Ctr; University of Texas El Paso	USA	Medicine			USA - one state		yes
Shimizu, Ikuo; Matsuyama, Yasushi; Duvivier, Robbert; van der Vleuten, Cees	Perceived positive social interdependence in online versus face-to-face team-based learning styles of collaborative learning: a randomized, controlled, mixed-methods study	BMC MEDICAL EDUCATION	2022	10.1186/s12909-022-03633-y	Shinshu University; Jichi Medical University; University of Groningen; Maastricht University	Netherlands+Japan	Medicine				Japan+Netherlands	
Feng, Yonghui; Zhao, Bin; Zheng, Jun; Fu, Yajing; Jiang, Yongjun	Online flipped classroom with team-based learning promoted learning activity in a clinical laboratory immunology class:	BMC MEDICAL EDUCATION	2022	10.1186/s12909-022-03917-3	China Medical University; China Medical University; China Medical University; China Medical University; China Medical University	China	Medicine		one institution, many authors			

	response to the COVID-19 pandemic											
Hur, Yera; Yeo, Sanghee; Lee, Keumho	Medical students' self-evaluation of character, and method of character education	BMC MEDICAL EDUCATION	2022	10.1186/s12909-022-03342-6	Hallym University; Kyungpook National University (KNU); Korea University of Technology & Education	South Korea	Medicine			South Korea		
Imran, Muhammad; Halawa, Taher Fawzy; Baig, Mukhtiar; Almanjoumi, Ahmed Mohammed; Badri, Mohammed Mustafa; Alghamdi, Waleed Ahmed	Team-based learning versus interactive lecture in achieving learning outcomes and improving clinical reasoning skills: a randomized crossover study	BMC MEDICAL EDUCATION	2022	10.1186/s12909-022-03411-w	King Abdulaziz University; King Abdulaziz University; King Abdulaziz University; King Abdulaziz University; King Abdulaziz University	Saudi Arabia	Medicine			Saudi Arabia		
Palmer, Russell H.; Moulton, Morgan K.; Stone, Rebecca H.; Lavender, Devin L.; Fulford, Michael; Phillips, Beth Bryles	The impact of synchronous hybrid instruction on students engagement in a pharmacotherapy course	PHARMACY PRACTICE-GRANADA	2022	10.18549/PharmPract.2022.1.2611	University of Georgia; University of Georgia; University of Georgia; University of Georgia; University of Georgia; Dept Vet Affairs	USA	Pharmacy			USA - multiple states		
Lakhtakia, Ritu; Otaki, Farah; Alsuwaidi, Laila; Zary, Nabil	Assessment as Learning in Medical Education: Feasibility and Perceived Impact of Student-Generated Formative Assessments	JMIR MEDICAL EDUCATION	2022	10.2196/35820	Mohammed Bin Rashid Univ Med & Hlth Sci; Mohammed Bin Rashid Univ Med & Hlth Sci; Mohammed Bin Rashid Univ Med & Hlth Sci; Mohammed Bin Rashid Univ Med & Hlth Sci	UAE	Medicine		one institution, many authors			
Kadavakollu, Samuel; Lund, Krista S.; Swamy, Varsha; Kim, William D.; Llenado, Ronald A.; Nunes, Taylor F.; Qureshi, Mahboob; Graneto, John W.; Boyanovsky, Boris B.	Promoting cultural competency and osteopathic medicine awareness among premedical students through a summer premedical rural enrichment program	JOURNAL OF OSTEOPATHIC MEDICINE	2022	10.1515/jom-2022-0002	Kaiser Permanente; California Health Sciences University (CHSU); California Health Sciences University (CHSU); California Health Sciences University (CHSU); California Health Sciences University (CHSU); California Health Sciences University (CHSU); ; California Health Sciences University (CHSU); ; California Health Sciences University (CHSU); ; California Health Sciences University (CHSU)	USA	Medicine			USA - multiple states		
Song, Eunkyoung; Issenberg, S. Barry; Roh, Young Sook	Effects of Prebriefing Using Online Team-Based Learning in Advanced Life Support Education for Nurses	CIN-COMPUTERS INFORMATICS NURSING	2023	10.1097/CIN.0000000000001056	Seoul National University; University of Miami; Chung Ang University; Chung Ang University	USA+South Korea	Nursing				South Korea+USA	
Sanad, Alqarni Aida; El-Sayed, Sahar Hamdy; Bassuni, Enas M.; Ahmed, Kawther Eitayeb	Effect of Team Based Learning on Classroom Engagement, Critical thinking dispositions And Academic Achievement of Nursing Students Enrolled in Principle of Nursing Research Course	JOURNAL OF POPULATION THERAPEUTICS AND CLINICAL PHARMACOLOGY	2023	10.47750/jptcp.2023.30.07.027	King Khalid University; King Khalid University; King Khalid University; King Khalid University; King Khalid University;	Saudi Arabia	Pharmacy		one institution, many authors			
Kweon, Young-Ran; Park, Jungrim	Using the design-thinking method to develop and validate a peer evaluation scale for team-based learning (PES-TBL) for nursing students	NURSE EDUCATION TODAY	2023	10.1016/j.nedt.2023.105849	Chonnam National University; Chonnam National University; Hlth Insurance Review & Assessment Serv	South Korea	Nursing			South Korea		
Murata, Hiroaki; Asakawa, Shoko; Kawamura, Takao; Yamauchi, Hideki; Takahashi, Osamu; Henker, Richard	Efficacy of modified team-based learning in a flipped classroom for an acute-care nursing course: A mixed-methods study	NURSING OPEN	2023	10.1002/nop2.1730	National Defense Medical College - Japan; Keio University; International University of Health & Welfare; Toho University; St. Luke's International Hospital; St. Luke's International Hospital; University of Pittsburgh; National Defense Medical College - Japan	USA+Japan	Nursing				Japan+USA	
Wannasai, Komson; Rottuntikarn, Wisanu; Sae-ung, Atiporn; Limsopatham, Kwankamol; Dankai, Wiyada	Preclinical medical student satisfaction of Team-based learning in Chiang Mai University	ASIA PACIFIC SCHOLAR	2023	10.29060/TAPS.2023-8-4/SC3000	Chiang Mai University; Chiang Mai University; Chiang Mai University; Chiang Mai University; Chiang Mai University	Thailand	Medicine		one institution, many authors			
Guraya, Shaista Salman; Guraya, Salman Yousuf; Doubell, Fiza-Rashid; Mathew, Bincy; Clarke, Eric; Ryan, Aine; Fredericks, Salim; Smyth, Mary; Hand, Sinead; Al-Qallaf, Amal; Kelly, Helen; Harkin, Denis W.	Understanding medical professionalism using express team-based learning; a qualitative case-based study	MEDICAL EDUCATION ONLINE	2023	10.1080/10872981.2023.2235793	Royal College of Surgeons in Ireland; Royal College of Surgeons in Ireland; Royal College of Surgeons in Ireland; Royal College of Surgeons in Ireland; Royal College of Surgeons in Ireland; Royal College of Surgeons in Ireland; Medical University of Bahrain; Medical University of Bahrain; Medical University of Bahrain; Medical University of Bahrain; Medical University of Bahrain; Medical University of Bahrain; University of Sharjah	Bahrain+UAE+Ireland	Medicine				Bahrain+Ireland+UAE	
Shoair, Osama A.; Smith, Winter J.; Aziz, May Abdel H.; Veronin, Michael A.; Glavy, Joseph S.; Pirtle, Shelby J.	Pharmacy students' perceptions and attitudes toward face-to-face vs. virtual team-based learning (TBL) in the didactic curriculum: A mixed-methods study	MEDICAL EDUCATION ONLINE	2023	10.1080/10872981.2023.2226851	University of Texas at Tyler; University of Texas at Tyler; University of Texas at Tyler; University of Texas at Tyler; University of Texas at Tyler; University of Texas at Tyler	USA	Pharmacy		one institution, many authors			
Tsai, Jung-Mei; Chen, Chin-Hsing; Hsu, Chen-Yuan; Liao, Hui-Chuan; Tsai, Li-Yun	Team-based learning complemented by interactive response system: Application of a strategy on the course of human growth and development for	TAIWANESE JOURNAL OF OBSTETRICS & GYNECOLOGY	2023	10.1016/j.tjog.2022.11.018	Da Yeh University; Mackay Medical College; Central Taiwan University Science & Technology; Da Yeh University; China Med Univ Beigang; Central Taiwan University Science & Technology; Taiwan University Science & Technology	Taiwan	Nursing			Taiwan		

	nursing students											
Attia, Reem T.; Mandour, Asmaa A.	Team-based learning-adopted strategy in pharmacy education: pharmacology and medicinal chemistry students' perceptions	FUTURE JOURNAL OF PHARMACEUTICAL SCIENCES	2023	10.1186/s43094-023-00464-6	Future University in Egypt; Future University in Egypt	Egypt	Pharmacy		one institution, many authors			
Mann, Adrienne W.; Cunningham, John; Tumolo, Alexis; King, Christopher	Evaluating a Blended Learning Model for Medical Student ECG Teaching	SOUTHERN MEDICAL JOURNAL	2023	10.14423/SMJ.0000000000001496	Rocky Mt Reg Vet Affairs Med Ctr; Denver Health Medical Center; University of Colorado; University of Colorado; University of Colorado;	USA	Medicine			USA - one state		
Silva, Cianny Ximenes Rodrigues; Belfort, Marcia Guelma Santos	BIOCHEMISTRY USING TEAM-BASED LEARNING (GLA) AND LEARNING PATH: EXPERIENCE REPORT	HUMANIDADES & INOVACAO	2023		Univ Estadual Tocantins Unitins; Univ Estadual Tocantins Unitins	Brazil	Medicine		one institution, many authors			
Rhodes, Kathleen A.; Carr, Kayla; McElwain, Sharon; Stewart, Mary W.	Preparing for Next Generation NCLEX® Through Team-Based Learning: Student Perspectives	NURSING EDUCATION PERSPECTIVES	2023	10.1097/01.NEP.0000000000000904	University of Mississippi; University of Mississippi; University of Mississippi; University of Mississippi	USA	Nursing		one institution, many authors			
Kushida, Simone Sayuri; Troster, Eduardo Juan	Burnout prevalence in medical students attending a team-based learning school	FRONTIERS IN EDUCATION	2023	10.3389/feduc.2023.1091426	Hospital Israelita Albert Einstein; Hospital Israelita Albert Einstein	Brazil	Medicine		one institution, many authors			
Clarke, Antonia J.; Burgess, Annette; Van Diggele, Christie; Bloomfield, Jacqueline; Schneider, Carl	Improving Patient Safety: Engaging Students in Interprofessional Team-Based Learning (TBL)	JOURNAL OF UNIVERSITY TEACHING AND LEARNING PRACTICE	2023		University of Sydney; University of Sydney; University of Sydney; University of Sydney; University of Sydney	Australia	Interprofessional		one institution, many authors			
Troya Altamirano, Carlos Alejandro; Betancourt Rubio, Evelyn Carolina; Ezcurdia Barzaga, Manuel Conrado	CONSIDERATIONS AND OBSTACLES IN THE IMPLEMENTATION OF A TEAM-BASED LEARNING PROGRAM IN THE SUBJECT OF SCIENTIFIC RESEARCH IN THE FIELD OF MEDICINE	REVISTA CONRADO	2023		Univ Reg Autonoma Los Andes; Univ Reg Autonoma Los Andes; Univ Reg Autonoma Los Andes	Ecuador	Medicine		one institution, many authors			
Saitoh, Aya; Yokono, Tomoe; Sumiyoshi, Tomoko; Kawachi, Izumi; Uchiyama, Mieko	A Comparative Study of Face-to-Face and Online Interprofessional Education Models for Nursing Students in Japan: A Cross-Sectional Survey	EDUCATION SCIENCES	2023	10.3390/educsci13090937	Niigata University; Niigata University; Niigata University; Niigata University; Niigata University	Japan	Nursing		one institution, many authors			
Jackson, Lisa; Otaki, Farah	Using team-based learning to optimize undergraduate family medicine clerkship training: mixed methods study	BMC MEDICAL EDUCATION	2023	10.1186/s12909-023-04240-1	Brunel Med Sch; Mohammed Bin Rashid Univ Med & Hlth Sci	UK+UAE	Medicine				UK+UAE	
Lerchenfeldt, Sarah; Kamel-ElSayed, Suzan; Patino, Gustavo; Loftus, Stephen; Thomas, David M. M.	A Qualitative Analysis on the Effectiveness of Peer Feedback in Team-Based Learning	MEDICAL SCIENCE EDUCATOR	2023	10.1007/s40670-023-01813-z	Oakland University; Oakland University; Oakland University; Oakland University; Western Michigan University	USA	Medicine			USA - one state		
Goh, Choon Fu; Ong, Eng Tek	Comparisons of analogical learning and team discussion as interactive in-class activities in flipped classroom of a pharmacy compounding course	INNOVATIONS IN EDUCATION AND TEACHING INTERNATIONAL	2023	10.1080/14703297.2023.2252391	Universiti Sains Malaysia; UCSI University; Universiti Sains Malaysia	Malaysia	Pharmacy			Malaysia		
Iida, Risa; Tanaka, Mai; Torigoe, Minami; Inaba, Sakiko; Wakana, Noriaki; Homma, Kazuhiro; Fukuyama, Naoto	Team-based learning impact: A comparative study of student and faculty facilitators	EDUCATION FOR HEALTH	2023	10.4103/efh.efh_191_23	Tokyo University of Agriculture; Tokyo University of Agriculture; Tokyo University of Agriculture; Tokyo University of Agriculture; Tokyo University of Agriculture; Tokyo University of Agriculture; Tokyo University of Agriculture	Japan	Medicine		one institution, many authors			
Carris, Nicholas W.; Cole, Jaclyn D.; Snyder Franklin, Ann; Sunjic, Katlynd M.	Vancomycin Flight Simulator: A Team-Based Learning Exercise	PHARMACY	2023	10.3390/pharmacy11010013	University of South Florida; University of South Florida; University of South Florida; Southwestern Vermont Med Ctr	USA	Pharmacy			USA - multiple states		
Chang, Mei-Chuan; Yu, Jui-Hung; Hsieh, Jyh-Gang; Wei, Mi-Hsiu; Wang, Ying-Wei	Effectiveness of the refined health literacy course on improving the health literacy competencies of undergraduate nursing students: quantitative and qualitative perspectives	MEDICAL EDUCATION ONLINE	2023	10.1080/10872981.2023.2173042	Tzu Chi University; Tzu Chi University; Hualien Tzu Chi Hospital; Hualien Tzu Chi Hospital; Tzu Chi University; Tzu Chi University; Tzu Chi University	Taiwan	Nursing			Taiwan		
El-Ashkar, Ayman M.; Aboregela, Adel M.; Alam-Eldin, Yosra H.; Metwally, Ashraf S.	Team-based learning as an inspiring tool for teaching Parasitology in the integrated curricula	PARASITOLOGISTS UNITED JOURNAL	2023	10.21608/puj.2023.192898.1200	Ain Shams University; Ain Shams University; Ain Shams University; Ain Shams University; University of Bisha; University of Bisha; Zagazig University	Saudi Arabia+ Egypt	Medicine				Egypt+Saudi Arabia	

Jain, Amit Kumar; Jain, Naveen; Jain, Seema	Perception of Undergraduate Medical Students and Faculty towards Team Based Learning as a Teaching Tool- A Cross-sectional Study	JOURNAL OF CLINICAL AND DIAGNOSTIC RESEARCH	2023	10.7860/JCDR/2023/61590.17714	Rajshree Med Res Inst; Rajshree Med Res Inst; UCMS & GTB Hosp	India	Medicine			India		
Pakhmode, Smita; Chandankhede, Manju; Dashputra, Amruta; Gupta, Madhur; Panbude, Swati; Timalsina, DIlIP R.	Team-based Learning versus Problem-based Learning among First-year Medical Students in Biochemistry: A Quasi-experimental Study	JOURNAL OF CLINICAL AND DIAGNOSTIC RESEARCH	2023	10.7860/JCDR/2023/64904.18754	NKP Salve Med Coll; Datta Meghe Institute of Higher Education & Research; Datta Meghe Institute of Higher Education & Research; NKP Salve Med Coll; NKP Salve Med Coll; Jawaharlal Nehru Medical College Wardha	India	Medicine			India		
Liang, Shuai; Wu, Huiwen; Deng, Shihao; Jun, Li	The application of flipped classroom combined with team-based learning in the orthopedic clinical teaching	MEDICINE	2023	10.1097/MD.0000000000035803	Anhui Medical University; Anhui Medical University; Anhui Medical University; Anhui Medical University	China	Medicine		one institution, many authors			
Mulugeta, Hailemariam; Zemedkun, Abebayehu	Implementation of Team-Based Learning for a Clinical Module of the Ethiopian Undergraduate Anesthesia Curriculum and Students' Perspectives: A Pilot Cross-Sectional Study	ADVANCES IN MEDICAL EDUCATION AND PRACTICE	2023	10.2147/AMEP.S437710	Dilla University; Dilla University	Ethiopia	Medicine		one institution, many authors			
Khalafalla, Farid G.; Eichmann, Kelly K.; VanGarsse, Anne; Ofstad, William	Nutrition and Lifestyle Coaching: An Interprofessional Course for Pharmacy, Medical, and Dietetic Students	CUREUS JOURNAL OF MEDICAL SCIENCE	2023	10.7759/cureus.48302	Touro University California; Clovis Unified Sch Dist; Univ Calif Riverside; West Coast Univ	USA	Medicine			USA - one state		
Richards, Sherese	Student Engagement Using HoloLens Mixed-Reality Technology in Human Anatomy Laboratories for Osteopathic Medical Students: an Instructional Model	MEDICAL SCIENCE EDUCATOR	2023	10.1007/s40670-023-01728-9	California Health Sciences University (CHSU)	USA	Medicine	one author				
Hagumimana, Justin; Skelton, Teresa; Pendergrast, Jacob; Nizeyimana, Francoise; Masaisa, Florence; Kanyamuhunga, Aimable; Gashaija, Christopher; Charge, Sophie; Kapitany, Casey; Morgan, Mary; Meirovich, Harley; Lin, Yulia	Transfusion Camp Rwanda: A prospective feasibility study evaluating the delivery of Transfusion Camp to a multidisciplinary group of postgraduate medical trainees in Rwanda	TRANSFUSION	2023	10.1111/trf.17568	University of Rwanda; University of British Columbia; University of Toronto; University of Toronto; University of Toronto; University of Toronto; University of Toronto; Kigali Univ; University of Rwanda; University of Rwanda; University of Rwanda; Canadian Blood Services; Sunnybrook Health Science Center	Canada +Rwanda	Medicine				Canada+Rwanda	
Roberts, Joel; Zhong, Qing; Linger, Rachel	Integrating Foundational and Clinical Science Remotely by Combining Team-Based Learning and Simulation	MEDICAL SCIENCE EDUCATOR	2023	10.1007/s40670-023-01817-9	Rocky Vista Univ; Rocky Vista Univ; Rocky Vista Univ	USA	Medicine		one institution, many authors			
Cavalcante, Felipe; Fonseca, Cynthia; Luz, Elba; Ramos, Laiz; Silva, Larissa; Soares, Leticia; Lima, Maria Eduarda; Albuquerque, Priscila	Active methods, laboratory experience, and clinical correlations articulated with the learning of Biochemistry: innovating the teaching method at the University of Pernambuco Campus Garanhuns	REVISTA DE ENSINO DE BIOQUIMICA	2023	10.16923/reb.v21i1.1016	Universidade de Pernambuco (UPE); Universidade de Pernambuco (UPE); Universidade de Pernambuco (UPE); Universidade de Pernambuco (UPE); Universidade de Pernambuco (UPE); Universidade de Pernambuco (UPE); Universidade de Pernambuco (UPE); Universidade de Pernambuco (UPE)	Brazil	Medicine		one institution, many authors			
Carr, Kayla; Rhodes, Kathleen A.; Klamm, Melissa M.; McElwain, Sharon	Engaging Nursing Students in Mental Health Concepts Through Multiple Teaching Modalities	JOURNAL OF NURSING EDUCATION	2023	10.3928/01484834-20230315-01	University of Mississippi; University of Mississippi; University of Mississippi; University of Mississippi	USA	Nursing		one institution, many authors			
Abouzeid, Enjy; Sallam, Moataz A.	Teaching by concordance: Individual versus team-based performance	INNOVATIONS IN EDUCATION AND TEACHING INTERNATIONAL	2023	10.1080/14703297.2022.2061568	Suez Canal University; Suez Canal University	Egypt	Medicine		one institution, many authors			
Fujiwara, Yuki; Amano, Izuki; Ishii, Sumiyasu; Kishi, Mikiko; Koibuchi, Noriyuki	Online Physiology Practice with Team-Based Learning During the COVID-19 Pandemic	ADVANCES IN MEDICAL EDUCATION AND PRACTICE	2023	10.2147/AMEP.S415257	Gunma University; Gunma University; Gunma University; Gunma University; Gunma University	Japan	Medicine		one institution, many authors			
Vernon, Veronica P.; Cieri-Hutcherson, Nicole E.; Arellano, Regina; Collins, Olivia; Lodise, Nicole M.	Contraception for transgender and gender diverse individuals in pharmacy education: A cross-sectional survey and select resources	CURRENTS IN PHARMACY TEACHING AND LEARNING	2023	10.1016/j.cptl.2023.07.010	Butler University; University Buffalo; Midwestern University; Nebraska Med; Albany College of Pharmacy & Health Sciences	USA	Pharmacy			USA - multiple states		
Forbes, Karen L.; Foulds, Jessica L.	A Team-based Learning Approach During Pediatric Clerkship to Promote Clinical Reasoning	ACADEMIC PEDIATRICS	2023	10.1016/j.acap.2023.04.002	University of Alberta; University of Alberta	Canada	Medicine		one institution, many authors			
Lingow, Sara; Jeon, Michelle; Richter, Sara K.	Evaluation of Student Peer- and Self-Grading in an Integrated Pharmacotherapy	AMERICAN JOURNAL OF PHARMACEUTICAL EDUCATION	2023	10.1016/j.ajpe.2023.100618	Univ Hlth Sci & Pharm; Univ Hlth Sci & Pharm; Saint Joseph's University	USA	Pharmacy		one institution, many authors			

	Course											
Xiao, Chen-Lin; Ren, Huan; Chen, Hui-Qing; Liu, Wen-Hui; Luo, Zhi-Ying; Li, Wen-Ru; Luo, Jian-Quan	Multidimensional evaluation of teaching strategies for pharmacology based on a comprehensive analysis involving 21,269 students	FRONTIERS IN PHARMACOLOGY	2023	10.3389/fphar.2023.1145456	Central South University; Central South University; Central South University; Central South University; Central South University; Hunan Normal University	China	Pharmacy			China		
Lau, Kai Yuan; Ang, Jessica Yang Huey; Rajalingam, Preman	Very Short Answer Questions in Team-Based Learning: Limited Effect on Peer Elaboration and Memory	MEDICAL SCIENCE EDUCATOR	2023	10.1007/s40670-022-01716-5	Nanyang Technological University; Nanyang Technological University; Nanyang Technological University	Singapore	Medicine		one institution, many authors			
Li, Zhiying; Cai, Xiaoyan; Zhou, Kebing; Qin, Jieying; Zhang, Jiahui; Yang, Qiaohong; Yan, Fengxia	Effects of BOPPPS combined with TBL in surgical nursing for nursing undergraduates: a mixed-method study	BMC NURSING	2023	10.1186/s12912-023-01281-1	Jinan University; Jinan University; Jinan University; Jinan University; Jinan University; Jinan University; Jinan University; Jinan University	China	Nursing		one institution, many authors			
Van Winkle, Lon J.; Rogers, Shane L.; Thornock, Bradley O.; Schwartz, Brian D.; Horst, Alexis; Fisher, Jensen A.; Michels, Nicole	Survey of attitudes toward performing and reflecting on required team service-learning (SASL): psychometric data and reliability/validity for healthcare professions students in preclinical courses	FRONTIERS IN MEDICINE	2023	10.3389/fmed.2023.1282199	Rocky Vista Univ; Rocky Vista Univ; Rocky Vista Univ; Rocky Vista Univ; Rocky Vista Univ; Midwestern University; Edith Cowan University; Rocky Vista Univ	Australia+USA	Medicine				Australia+USA	
Woon, Luke Sy-Cherng; Daud, Tuti Iryani Mohd; Tong, Seng Fah	It kinda helped us to be there: students' perspectives on the use of virtual patient software in psychiatry posting	BMC MEDICAL EDUCATION	2023	10.1186/s12909-023-04834-9	Universiti Kebangsaan Malaysia; Universiti Kebangsaan Malaysia; Universiti Kebangsaan Malaysia	Malaysia	Medicine		one institution, many authors			
Smith Jr, James F.; Sinclair, Mary L.; Madhavan, Kiely M.; Eno, Cassie A.; Piemonte, Nicole M.	Learning How to Learn: An Innovative Medical School Orientation Activity	ACADEMIC MEDICINE	2023	10.1097/ACM.0000000000005211	Creighton University; Creighton University; Creighton University; Creighton University; Creighton University	USA	Medicine		one institution, many authors			
Mizumoto, Junki; Son, Daisuke; Izumiya, Masashi; Horita, Shoko; Eto, Masato	The impact of patients' social backgrounds assessment on nursing care: Qualitative research	JOURNAL OF GENERAL AND FAMILY MEDICINE	2023	10.1002/jgf2.650	University of Tokyo; University of Tokyo; University of Tokyo; Tottori University; Teikyo University; University of Tokyo	Japan	Nursing			Japan		
Aboregela, Adel M.; Sonpol, Hany M. A.; Metwally, Ashraf S.; El-Ashkar, Ayman M.; Hashish, Abdullah A.; Mohammed, Osama A.; Elnahriry, Tarek A.; Senbel, Ahmed; Alghamdi, Mushabab	Medical students' perception and academic performance after team- based and seminar-based learning in human anatomy	JOURNAL OF TAIBAH UNIVERSITY MEDICAL SCIENCES	2023	10.1016/j.jtumed.2022.08.005	University of Bisha; University of Bisha; University of Bisha; University of Bisha; University of Bisha; Zagazig University; Mansoura University; Zagazig University; Ain Shams University; Suez Canal University; Ain Shams University; University of Bisha; University of Bisha; Suez Canal University; University of Bisha; University of Bisha	Saudi Arabia+Egypt	Medicine				Egypt+Saudi Arabia	
Ulfa, Yunefit; Horiuchi, Shigeko; Shishido, Eri; Igarashi, Yukari	Team-based learning in Indonesian midwifery education: Implementation research	JAPAN JOURNAL OF NURSING SCIENCE	2024	10.1111/jjns.12587	St. Luke's International Hospital; St. Luke's International Hospital; St. Luke's International Hospital; Natl Res & Innovat Agcy	Indonesia+Japan	Nursing				Indonesia+Japan	
Hu, Longyi; Li, Siqi; Zhou, Leshan	Effect of Tronclass combined with team-based learning on nursing students' self-directed learning and academic performance: a pretest-posttest study	BMC MEDICAL EDUCATION	2024	10.1186/s12909-024-05741-3	Central South University; Central South University; Central South University	China	Nursing		one institution, many authors			
Ahmed, Elshafa Mohamed; Abdulrahman, Nahla Elradhi; Galgam, Fatima Alzahra Abdul Rahman; Shuib, Sharfeldin Mohammed; Abuanga, Mohammed Jeber elder; Elfaki, Nahid Khalil	Nursing Students' Perception Toward Team-based Learning at Nile University, Sudan	SUDAN JOURNAL OF MEDICAL SCIENCES	2024	10.18502/sjms.v19i1.15787	Najran University; Najran University; Najran University; Int Univ Africa; Int Univ Africa; Int Univ Africa	Saudi Arabia+Sudan	Nursing				Saudi Arabia+Sudan	
Luitjes, Nora L. D.; van der Velden, Gisela J.; Pandit, Rahul	Using Team-Based Learning to Teach Pharmacology within the Medical Curriculum	PHARMACY	2024	10.3390/pharmacy12030091	Utrecht University; Utrecht University; Utrecht University	Netherlands	Pharmacy		one institution, many authors			
Medlinskiene, Kristina; Hill, Suzanne; Tweddell, Simon; Quinn, Gemma	Evaluating team-based learning in a foundation training pathway for trainee pharmacists	INTERNATIONAL JOURNAL OF PHARMACY PRACTICE	2024	10.1093/ijpp/riae006	University of Bradford; University of Bradford; University of Bradford; University of Bradford; Fdn Training Consortium; Hull Univ Teaching Hosp & NHS Trust	UK	Pharmacy			England		
Shoair, Osama A.	Academic performance among pharmacy students using virtual vs. face-to-face team-based learning	ANNALS OF MEDICINE	2024	10.1080/07853890.2024.2349205	University of Texas at Tyler	USA	Pharmacy	one author				
Karaca, Adeviyye; Daloglu, Mustafa; Kilic, Deniz; Sivil, Ramazan; Kesapli, Mustafa; Alimoglu, Mustafa Kemal	Training interprofessional teams in geriatric emergency medicine: A modified team-based learning approach	HELIYON	2024	10.1016/j.heliyon.2024.e25099	University of Health Sciences Turkey; University of Health Sciences Turkey; University of Health Sciences Turkey; University of Health Sciences Turkey; Akdeniz University; Akdeniz University	Turkey	Interprofessional			Turkey		

Aimiya, Yukinori; Mizuno, Tomohiro; Sakakibara, Mikio; Matsumoto, Noriaki; Sugiura, Shin-Ya; Mizokami, Fumihiro; Lee, Jeannie K.; Yamada, Shigeki	Effectiveness of Online Team-based Learning for Pharmacists on How to Conduct Clinical Medication Reviews for Old Patients in Japan: A Randomized Controlled Trial	IN VIVO	2024	10.21873/invivo.13460	Fujita Health University; Fujita Health University; Fujita Health University; Fujita Health University; Sugi Pharm Co Ltd; Sugi Pharm Co LtdYa] Sugi Pharm Co Ltd; Sugi Pharm Co Ltd; University of Arizona; Fujita Health University	USA+Japan	Pharmacy				Japan+USA	
Garb, Madeline; Jenkins, Melissa; Cruz, Elaine	Team-based learning in the internal medicine clerkship didactics	CLINICAL TEACHER	2024	10.1111/tct.13715	Case Western Reserve Univ; Case Western Reserve University; Ohio University; Ohio University	USA	Medicine			USA - one state		
Tuin, Ashley M.; Schechter, Thomas; Eno, Cassie A. H.	The Relationship Between Engagement Time in Case-based Learning and Performance on Preclinical Medical Education Exams	MEDICAL SCIENCE EDUCATOR	2024	10.1007/s40670-024-02112-x	Creighton University; Creighton University; Creighton University	USA	Medicine		one institution, many authors			
Rotgans, Jerome I.; Sterpu, Irene; Herling, Lotta; Nordquist, Jonas; Acharya, Ganesh	Exploring the dynamics of situational interest in team-based learning in undergraduate medical education	BMC MEDICAL EDUCATION	2024	10.1186/s12909-024-05769-5	Karolinska Institutet; Karolinska Institutet; Karolinska University Hospital;Karolinska University Hospital; Karolinska Univ Hosp; Karolinska Univ Hosp; UiT The Arctic University of Tromso; Erasmus University Rotterdam	Sweden+Norway+Netherlands	Medicine				Netherlands+Norway+Sweden	
Sterpu, Irene; Herling, Lotta; Nordquist, Jonas; Moller, Anna; Kallner, Helena Kopp; Engberg, Hedvig; Acharya, Ganesh	The outcomes of team-based learning versus small group interactive learning in the obstetrics and gynecology course for undergraduate students	ACTA OBSTETRICIA ET GYNECOLOGICA SCANDINAVICA	2024	10.1111/aogs.14804	Karolinska Institutet; Karolinska Institutet; Karolinska Institutet; Karolinska Institutet; Karolinska Institutet; Karolinska Institutet; Karolinska Institutet; Karolinska University Hospital; Karolinska University Hospital; Stockholm South Hosp; Danderyd Hosp; UiT Arctic Univ Norway	Sweden+Norway	Medicine				Norway+Sweden	
Wu, Ping; Zhou, Yiman; Lv, Wenjie	Abdominal injuries: Online team-based training of senior medical students is preferred over online class-based training	WORLD JOURNAL OF SURGERY	2024	10.1002/wjs.12245	Shanghai Jiao Tong University; Shanghai Jiao Tong University; Shanghai Jiao Tong University	China	Medicine		one institution, many authors			
Tiako, Max Jordan Nguemeni; Aguilar, Gabriela; Adeyemo, Oluwatosin; Reynolds, Heather; Campbell, Katherine H.; Stanwood, Nancy; Galerneau, France	Developing an interactive reproductive health equity session for pre-clerkship medical students	MEDICAL EDUCATION ONLINE	2024	10.1080/10872981.2024.2364984	Brigham & Women's Hospital; NYU Langone Medical Center; Yale University; Yale University; Yale University; Yale University; Planned Parenthood Southern New England	USA	Medicine			USA - multiple states		
Shen, Jing; Qi, Hongyan; Mei, Ruhuan; Sun, Cencen	A comparative study on the effectiveness of online and in-class team-based learning on student performance and perceptions in virtual simulation experiments	BMC MEDICAL EDUCATION	2024	10.1186/s12909-024-05080-3	Zhejiang University; Zhejiang University; Zhejiang University; Zhejiang University	China	Medicine		one institution, many authors			
Alsahali, Saud; Almutairi, Salman; Almutairi, Salem; Almofadhi, Saleh; Anaam, Mohammed; Alshammari, Mohammed; Abdulsalim, Suhaj; Almogbel, Yasser	Pharmacy Students' Attitudes Toward Distance Learning After the COVID-19 Pandemic: Cross-Sectional Study From Saudi Arabia	JMIR FORMATIVE RESEARCH	2024	10.2196/54500	Qassim University; Qassim University; Qassim University; Qassim University; Qassim University; Qassim University; Qassim University; Qassim University	Saudi Arabia	Pharmacy		one institution, many authors			
Krishnan, Usha; Ravinder, Thyagarajan; Dhason, Therese Mary; Manikesi, Suganthi; Chithsabesan, Devi; Kalpanaraj, Dorairaj	Exploring the determinants of self-directed learning among medical undergraduates: A qualitative study	JOURNAL OF EDUCATION AND HEALTH PROMOTION	2024	10.4103/jehp.jehp_1105_23	Madras Medical College & General Hospital; Kilpauk Med Coll; Kilpauk Med Coll; Kilpauk Med Coll; Kilpauk Med Coll; Kilpauk Med Coll	India	Medicine		one institution, many authors			
Lee, Ching-Tien; Wang, Jiz-Yuh	Interactive audio human organ model combined with team-based learning improves the motivation and performance of nursing students in learning anatomy and physiology	ANATOMICAL SCIENCES EDUCATION	2024	10.1002/ase.2350	Hsin Sheng Jr Coll Med Care & Management; National Taipei University of Nursing & Health Science (NTUNHS); Kaohsiung Medical University	Taiwan	Nursing			Taiwan		
Zane, Hannah; Brunton, Amanda; Carney, Patricia A.; Haney, Elizabeth; Bonura, Erin M.	Team-Based Learning as a Feasible, Acceptable, and Effective Educational Method for Internal Medicine Trainees - A Multi-phase Intervention and Cohort Comparison	JOURNAL OF GENERAL INTERNAL MEDICINE	2024	10.1007/s11606-024-08891-8	Oregon Health & Science University; Oregon Health & Science University; Oregon Health & Science University; Oregon Health & Science University; Oregon Health & Science University	USA	Medicine		one institution, many authors			
Kamel-ElSayed, Suzan; Patino, Gustavo; Lerchenfeldt, Sarah	Self-Directed-Team Learning (SDTL) in Medical Education	MEDICAL SCIENCE EDUCATOR	2024	10.1007/s40670-024-02101-0	Oakland University; Oakland University; Western Michigan University	USA	Medicine			USA - one state		
Hancock, Laura M.	Student Perceptions of Team-Based Learning in an Advanced Inorganic Chemistry Course	JOURNAL OF CHEMICAL EDUCATION	2024	10.1021/acs.jchemed.3c00655	University of Birmingham; Keele University	UK	Medicine	one author				
Seidel, Holmsten Stephanie	Team-Based Learning in the Political Science Classroom: Comparing In-person and Online	JOURNAL OF POLITICAL SCIENCE EDUCATION	2024	10.1080/15512169.2023.2251621	University of Texas System; University of Texas Austin; University of Texas System; University of Texas Austin	USA	Medicine	one author				

	Environments											
Noel, Zachary R.; Kulo, Violet; Cestone, Christina; Jun, Hyun-Jin; Sweet, Michael; Kubitz, Karla A.; Gordes, Karen L.	Ready or Not: A Crossover Study of (Un)graded Individual Readiness Assurance Tests in Team-Based Learning	AMERICAN JOURNAL OF PHARMACEUTICAL EDUCATION	2024	10.1016/j.ajpe.2024.100670	University of North Carolina Chapel Hill; University of Maryland Baltimore; University of Maryland Baltimore; University of Maryland Baltimore; University of Maryland Baltimore; Northeastern University; Towson University	USA	Pharmacy			USA - multiple states		yes
Delage, Clement; Palayer, Maeva; Lerouet, Dominique; Besson, Valerie C.	Pharmacotrophy: a playful tournament for game- and team-based learning in pharmacology education - assessing its impact on students' performance	BMC MEDICAL EDUCATION	2024	10.1186/s12909-024-05157-z	Universite Paris Cite; Universite Paris Cite; Universite Paris Cite; Universite Paris Cite; Hop Lariboisiere Fernand Widal	France	Pharmacy			France		
Toema, Sara M.; Amer, Nihal S.; Jones, Paul C.	The impact of three learning methods on dental students' satisfaction and knowledge acquisition	JOURNAL OF DENTAL EDUCATION	2024	10.1002/jdd.13545	Pennsylvania Commonwealth System of Higher Education (PCSHE); Temple University; Egyptian Knowledge Bank (EKB); National Research Centre (NRC); Pennsylvania Commonwealth System of Higher Education (PCSHE); Temple University	USA+Egypt	dentistry				Egypt+USA	
Badge, Ankit; Chandankhede, Manju; Gajbe, Ujwal; Bankar, Nandkishor J.; Bandre, Gulshan R.	Employment of Small-Group Discussions to Ensure the Effective Delivery of Medical Education	CUREUS JOURNAL OF MEDICAL SCIENCE	2024	10.7759/cureus.52655	Datta Meghe Institute of Higher Education & Research (Deemed to be University); Datta Meghe Medical College; Datta Meghe Institute of Higher Education & Research (Deemed to be University); Datta Meghe Medical College; Datta Meghe Institute of Higher Education & Research (Deemed to be University); Datta Meghe Medical College; Datta Meghe Institute of Higher Education & Research (Deemed to be University); Datta Meghe Institute of Higher Education & Research (Deemed to be University)	India	Medicine		one institution, many authors			
Ernst, Kelsey; Thompson, Amy N.; Vandenberg, Amy	Relative exposure to psychiatric conditions and medications in pharmacy education	CURRENTS IN PHARMACY TEACHING AND LEARNING	2024	10.1016/j.cptl.2023.12.016	University of Michigan; University of Michigan; University of Michigan; Ascens Med Grp	USA	Pharmacy			USA - one state		
Thiyagarajan, Suseela; Saldanha, Prakash R. M.; Govindan, Radhakrishnan; Leena, K. C.; Prathyusha, P., V	Development of Agile Scrum Perception Tool to Evaluate Students' Opinions on Agile Methodology in Nursing Education	INTERNATIONAL JOURNAL OF APPLIED AND BASIC MEDICAL RESEARCH	2024	10.4103/ijabmr.ijabmr_423_23	National Institute of Mental Health & Neurosciences - India; National Institute of Mental Health & Neurosciences - India; National Institute of Mental Health & Neurosciences - India; Yenepoya (Deemed to be University); Yenepoya (Deemed to be University); National Institute of Mental Health & Neurosciences - India	India	Nursing			India		
Wood, Jamie L.; Stringham, Nicole	The UnEssay project as an enriching alternative to practical exams in pre-professional and graduate education	JOURNAL OF BIOLOGICAL EDUCATION	2024	10.1080/00219266.2022.2047098	Duke University; Duke University	USA	Medicine		one institution, many authors			
Song, Xiaomei; Elftman, Michael	Beyond Collaborative Learning: a Comparison of Small Groups in Face-to-Face and Online Settings	MEDICAL SCIENCE EDUCATOR	2024	10.1007/s40670-024-01983-4	Case Western Reserve University; Central Michigan University	USA	Medicine			USA - multiple states		
Wu, Yijun; Zheng, Yue; Feng, Baijie; Yang, Yuqi; Kang, Kai; Zhao, Ailin	Embracing ChatGPT for Medical Education: Exploring Its Impact on Doctors and Medical Students	JMIR MEDICAL EDUCATION	2024	10.2196/52483	Sichuan University; Sichuan University; Sichuan University; Sichuan University; Sichuan University; Sichuan University	China	Medicine		one institution, many authors			
Koc, Demet; Yanginci, Kemal; Yavuz, Melike	Climate change education: exploring knowledge, attitudes, and practices of medical students in a Turkish medical school	INTERNATIONAL JOURNAL OF HEALTH PROMOTION AND EDUCATION	2024	10.1080/14635240.2024.2349884	Acibadem Mehmet Ali Aydinlar Univ Kerem; Bahcesehir University; Bahcesehir University	Turkey	Medicine			Turkey		
Wei, Xuhong; Xu, Ting; Guo, Ruixian; Tan, Zhi; Xin, Wenjun	Physiology education in China: the current situation and changes over the past 3 decades	BMC MEDICAL EDUCATION	2024	10.1186/s12909-024-05395-1	Sun Yat Sen University; Sun Yat Sen University; Sun Yat Sen University; Sun Yat Sen University; Sun Yat Sen University	China	Medicine		one institution, many authors			
Carnevale, Kevin; Saxena, Ritcha; Talmon, Geoffrey A.; Lin, Amy; Padilla, Osvaldo; Kreisle, Regina A.	Pathology teaching in different undergraduate medical curricula within and outside the United States: a pilot study	ACADEMIC PATHOLOGY	2024	10.1016/j.acpath.2023.100102	University of Minnesota Duluth; University of Nebraska Medical Center; University of Illinois Chicago; Texas Tech University Health Science Center; Purdue University; Des Moines Univ	USA	Medicine			USA - multiple states		yes
Eldakhakhny, Basmah; Alamoudi, Aliaa A.; Gad, Hoda; Almoghrabi, Yousef; Shamrani, Taghreed; Daghistani, Hussam; Bima, Abdulhadi; Ajabnoor, Ghada; Alfayez, Fayza; Elsamanoudy, Ayman	Introducing Group Open-Book Exams as a Learning and Assessment Strategy in the Clinical Biochemistry Course for Medical Students	CUREUS JOURNAL OF MEDICAL SCIENCE	2024	10.7759/cureus.51792	King Abdulaziz University; King Abdulaziz University; King Abdulaziz University; King Abdulaziz University; King Abdulaziz University; King Abdulaziz University; King Abdulaziz University; King Abdulaziz University; Alexandria University; Mansoura University	Saudi Arabia+Egypt	Medicine				Egypt+Saudi Arabia	

Salih, Karimeldin M.; Al-Faifi, Jaber; Alamri, Mohannad M.; Mohamed, Osama A.; Khan, Sameer M.; Marakala, Vijaya; Mohammed, Nahid A.; Mohamed, Suaad E.; Hashish, Abdullah A.; Sonpol, Hany M. A.; Khalid, Tarig B. A.; Elhag, Wafa I.; Alameer, Ahmed Y.; Metwally, Ashraf S.; Yahia, Amar I. O.; Ibrahim, Mutasim E.; Taura, Magaji G.; El-Ashkar, Ayman M.	Comparing students' performance in self-directed and directed self-learning in College of Medicine, University of Bisha	JOURNAL OF TAIBAH UNIVERSITY MEDICAL SCIENCES	2024	10.1016/j.jtumed.2024.05.003	University of Bisha; University of Bisha; University of Bisha; University of Bisha; University of Bisha; University of Bisha; University of Bisha; Ain Shams University; University of Bisha; University of Bisha; Univ Gezira; Suez Canal University; Mansoura University; Al Neelain Univ; University of Bisha; Univ Kordofan; University of Bisha; University of Bisha; University of Bisha; University of Bisha; Ain Shams University; University of Bisha; University of Bisha; University of Bisha	Saudi Arabia+ Egypt+Sudan	Medicine				Egypt+Saudi Arabia+Sudan	
Surapaneni, Krishna Mohan	Innovative Self-directed, Problem-oriented, Lifelong learning, Integrated Clinical case Exercise (SPLICE) modules promote critical thinking skills, early clinical exposure, and contextual learning among first professional-year medical students	ADVANCES IN PHYSIOLOGY EDUCATION	2024	10.1152/advan.00211.2023	Panimalar Med Coll Hosp & Res Inst	India	Medicine	one author				
de Vries, Teun J.; Schoenmaker, Ton; Peferoen, Laura A. N.; Krom, Bastiaan P.; Bloemena, Elisabeth	Design and evaluation of an immunology and pathology course that is tailored to today's dentistry students	FRONTIERS IN ORAL HEALTH	2024	10.3389/froh.2024.1386904	University of Amsterdam; University of Amsterdam; Vrije Universiteit Amsterdam; Vrije Universiteit Amsterdam; Vrije Universiteit Amsterdam; Vrije Universiteit Amsterdam; Vrije Universiteit Amsterdam; Vrije Universiteit Amsterdam; University of Amsterdam; University of Amsterdam	Netherlands	Dentistry			Netherlands		
Rodriguez-Martin, Ivan; Condes, Emilia; Sanchez-Gomez, Judit; Azpeleta, Clara; Tutor, Antonio S.; Lesmes, Marta; Gal, Beatriz	Perceptions of co-teaching as a pedagogical approach to integrate basic and clinical sciences	FRONTIERS IN MEDICINE	2024	10.3389/fmed.2024.1383975	Universidad Francisco de Vitoria; European University of Madrid; European University of Madrid; European University of Madrid; European University of Madrid; European University of Madrid; European University of Madrid; San Pablo CEU University; San Pablo CEU University	SPain	Medicine			Spain		
Chu, Ginger; Pitt, Victoria; Cant, Robyn; Johnson, Amanda; Inder, Kerry	Students' evaluation of professional experience placement quality in a pre-registration nursing programme: A cross-sectional survey	NURSE EDUCATION IN PRACTICE	2024	10.1016/j.nepr.2024.103877	University of Newcastle; University of Newcastle;University of Newcastle;University of Newcastle; Federation University Australia; University of Newcastle	Australia	Nursing			Australia		
